# Plasma membrane damage limits replicative lifespan in yeast and induces premature senescence in human fibroblasts

**DOI:** 10.1038/s43587-024-00575-6

**Published:** 2024-02-22

**Authors:** Kojiro Suda, Yohsuke Moriyama, Nurhanani Razali, Yatzu Chiu, Yumiko Masukagami, Koutarou Nishimura, Hunter Barbee, Hiroshi Takase, Shinju Sugiyama, Yuta Yamazaki, Yoshikatsu Sato, Tetsuya Higashiyama, Yoshikazu Johmura, Makoto Nakanishi, Keiko Kono

**Affiliations:** 1 Okinawa Institute of Science and Technology Graduate University, Okinawa, Japan; 2 Department of Hematology-Oncology, Institute of Biomedical Research and Innovation, Foundation for Biomedical Research and Innovation at Kobe, Hyogo, Japan; 3 Core Laboratory, Graduate School of Medical Sciences, Nagoya City University, Nagoya, Japan; 4 Institute of Transformative Bio-Molecules (WPI-ITbM), Nagoya University, Nagoya, Japan; 5 Department of Biological Science, Graduate School of Science, University of Tokyo, Tokyo, Japan; 6 Division of Cancer Cell Biology, Institute of Medical Science, University of Tokyo, Tokyo, Japan

**Keywords:** Senescence, Cell-cycle exit, Ageing

## Abstract

Plasma membrane damage (PMD) occurs in all cell types due to environmental perturbation and cell-autonomous activities. However, cellular outcomes of PMD remain largely unknown except for recovery or death. In this study, using budding yeast and normal human fibroblasts, we found that cellular senescence—stable cell cycle arrest contributing to organismal aging—is the long-term outcome of PMD. Our genetic screening using budding yeast unexpectedly identified a close genetic association between PMD response and replicative lifespan regulations. Furthermore, PMD limits replicative lifespan in budding yeast; upregulation of membrane repair factors ESCRT-III (*SNF7*) and AAA-ATPase (*VPS4*) extends it. In normal human fibroblasts, PMD induces premature senescence via the Ca^2+^–p53 axis but not the major senescence pathway, DNA damage response pathway. Transient upregulation of ESCRT-III (CHMP4B) suppressed PMD-dependent senescence. Together with mRNA sequencing results, our study highlights an underappreciated but ubiquitous senescent cell subtype: PMD-dependent senescent cells.

## Abstract (web summary)

Plasma membrane damage (PMD) can induce cell death or be repaired, whereas other cell fates are not well explored. In this study, the authors found that PMD induces senescence in yeast and human fibroblasts in a manner that is distinct from DNA damage-dependent senescence. They present transcriptomic data suggesting that this PMD response may explain the origins of senescent cells near cutaneous wounds.

## Main

Cells experience a variety of perturbations on the plasma membrane, ranging from physical attack to pathogen invasion^1^. The plasma membrane is also damaged by physiological activities, including muscle contraction^1–3^. There is a growing appreciation that failed plasma membrane damage (PMD) response causes various diseases. For example, mutations in the PMD repair protein dysferlin can lead to one form of muscular dystrophy^4^, and mutations in the PMD response factor lipid scramblase TMEM16F cause Scott syndrome^5,6^.

The PMD response is classified into two simple outcomes of recovery or death. Regarding the recovery response, plasma membrane repair mechanisms, including Ca^2+^-dependent lysosomal fusion to the plasma membrane and the endosomal sorting complexes required for transport (ESCRT) complex-dependent membrane scission, have been extensively studied in multiple eukaryotic systems^1–4,7–11^. In contrast, the PMD-induced cell death response, pyroptosis, has been studied mostly in mammalian cells and in the context of the immune response or cancer treatment^12,13^. Although these studies revealed the molecular mechanisms underlying the survival or death response after PMD in each system, a unified view of PMD response in eukaryotes remains largely elusive, partly because of the lack of a universal PMD induction method. In particular, the PMD-inducing chemicals used in mammalian cells, such as streptolysin O (SLO) or perforin, cannot be easily adopted to cell types with a rigid cell wall, including yeast.

Yeast serves as an excellent genetic tool to comprehensively identify genes required for fundamental cellular processes in eukaryotes. Previously, we demonstrated that budding yeast is equipped with a mechanism for repairing laser-induced PMD^14,15^. Although laser damage is a universal PMD method applicable for both budding yeast and higher eukaryotes, it cannot be easily employed in large-scale analysis.

In the present study, we developed a simple PMD induction method that can be used both in budding yeast and human cultured cells. Using the assay, we performed the genome-wide screening using yeast and found that PMD limits replicative lifespan in budding yeast and induces premature senescence in normal human fibroblasts. Although cellular senescence is induced by various triggers, including DNA damage, telomere shortening and oncogene activation, little was known about PMD-dependent senescence (PMD-Sen). Time-resolved mRNA sequencing (mRNA-seq) results indicate that PMD-dependent senescent cells (PMD-Sen cells) have different gene expression profiles, including the upregulation of wound healing genes. PMD-Sen may explain the origin of senescent cells around cutaneous wounds in vivo^16^.

## Results

### A simple and universal plasma membrane damaging method

**Fig. 1:**
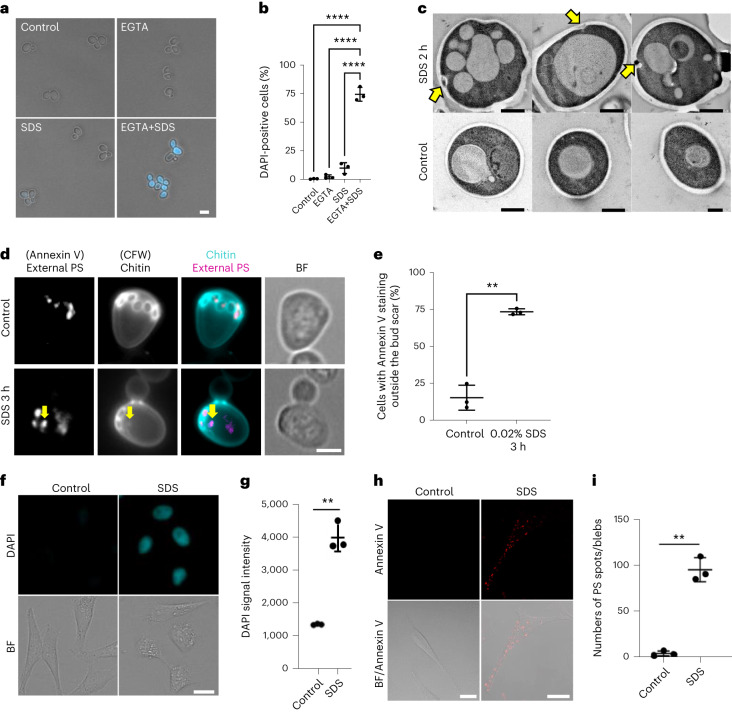
SDS induces PMD in yeast and human cells. **a**, Wild-type yeast cells were cultured in YPD and then incubated with 2 ng ml^−1^ DAPI-containing YPD with or without 20 mM EGTA and 0.02% SDS for 15 min. Scale bar, 5 μm. **b**, Quantification of **a**. Data are presented as mean (horizontal bars) ± s.d. (whiskers) of three independent experiments (*n* > 300 cells per each experiment). ****P < 0.001: control versus EGTA+SDS: <0.0001; EGTA versus EGTA+SDS: <0.0001; EGTA versus EGTA+SDS: <0.0001, by two-tailed unpaired Student’s *t*-test. **c**, Wild-type yeast cells were cultured at 25 °C and then incubated with 0.02% SDS for 2 h. Cells were fixed and observed by TEM. Yellow arrows, plasma membrane and cell wall ingression. Scale bar, 1 μm. **d**, Wild-type yeast cells were cultured in YPD and then switched to YPD containing 0.02% SDS for 3 h. Yeast cells were treated with zymolyase in 1.2 M sorbitol for 90 min and then stained with Annexin V–Alexa Fluor 568 for 20 min. Yellow arrows, Annexin V and Calcofluor white positive spots. **e**, The percentage of cells with Annexin V staining outside of the bud scar was counted. Data are presented as mean (horizontal bars) ± s.e.m. (whiskers) of three independent experiments. *n* > 250 cells per each experiment. **P < 0.01, exact value: 0.005, by two-tailed unpaired Student’s *t*-test. Scale bar, 2 μm. **f**, Representative images of HeLa cells with DAPI signals upon 0.008% SDS treatment for 24 h. The cells were cultured in a DAPI-containing medium with or without 0.008% SDS. Scale bar, 20 μm. **g**, Quantification of DAPI intensity as in **f**. Data are presented as mean (horizontal bars) ± s.d. (whiskers) of three independent experiments. Untreated control and 0.008% SDS treatment were significantly different (**P < 0.01, exact value: 0.0086) using two-sided multiple Welch’s *t*-test with Benjamini, Krieger and Yekutieli correction. See also Extended Data Fig. 2. **h**, Representative images of the HeLa cells with Annexin V–positive spots/blebs upon 0.008% SDS treatment. Cells were cultured in a medium with or without 0.008% SDS for 1 h. Scale bar, 20 μm. BF, bright-field. **i**, Numbers of Annexin V–positive spots/blebs were counted as in **h**. Data are presented as mean (horizontal bars) ± s.d. (whiskers) of three independent experiments (black dots). See also Extended Data Fig. 3a. **P < 0.01, exact value: 0.000292, by two-tailed unpaired Student’s *t*-test. Source data

To reveal the conserved features of the PMD response in eukaryotes, we needed a simple and reliable PMD-inducing method that can be (1) applicable for both budding yeast and human cells and (2) used in large-scale analyses. A candidate chemical was sodium dodecyl sulfate (SDS) because, previously, we showed that wild-type budding yeast cells can grow on a yeast extract peptone dextrose (YPD) plate containing 0.02% SDS, but yeast mutants that are defective in the PMD response fail to grow on it^14,15^. We tested whether SDS breaks cell wall and plasma membrane, which would lead to the penetration of a scarcely membrane-permeable fluorescent chemical, 4′,6-diamidino-2-phenylindole (DAPI). DAPI goes into only 9.8 ± 3.9% of wild-type budding yeast cells, consistent with the fact that wild-type budding yeast can reseal the wound immediately and survive in the presence of SDS. However, in combination with ethyl glycol tetraacetic acid (EGTA), which prevents Ca^2+^-dependent membrane resealing^9^, DAPI penetrated 74.5 ± 4.9% of cells (Fig. 1a,b). These results suggest that SDS breaks the plasma membrane, and the damage is immediately resealed by Ca^2+^-dependent mechanisms.

**Extended Data Fig. 1:**
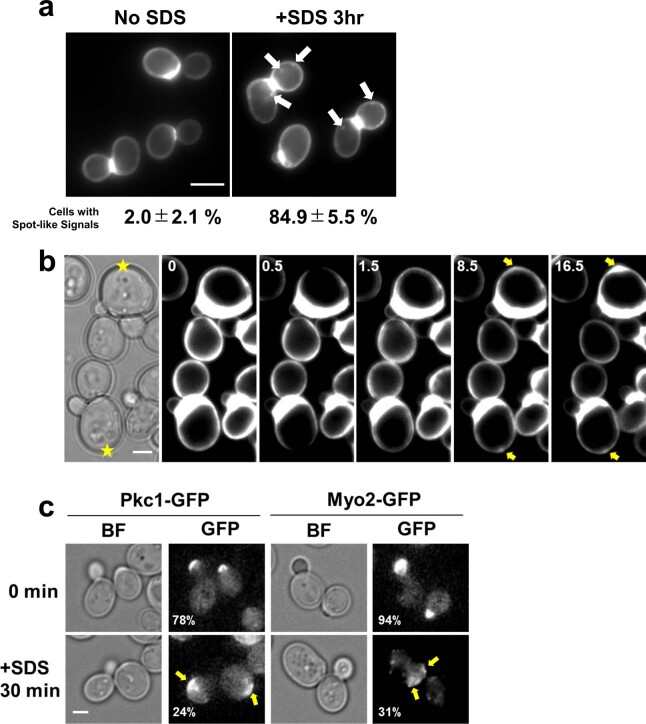
SDS induces local plasma membrane and cell wall damage in budding yeast. **(a)** Wild type yeast cells were cultured in YPD incubated with or without 0.02% SDS for 3 hr, and then stained with 20 μg/ml calcofluor white for 5 min. The values under the images are % cells with spot signals. Mean(SD) of four independent experiments. n > 100 cells/each experiment. *p* < 0.001 by 2-tailed unpaired Student’s *t*-test. Scale bar, 5 μm. **(b)** Wild type yeast cells were cultured at 25˚C under the microscope with 10 μg/ml calcofluor white and then laser damage was induced (yellow stars). Yellow arrows, calcofluor white signal accumulation. The numbers at the upper-left corner indicate time (min). Images were taken at 30 sec intervals. Scale bar, 2 μm. This experiment was independently repeated three times with similar results. This experiment was independently repeated three times with similar results. **(c)** Wild type yeast cells expressing Pkc1-GFP or Myo2-GFP were incubated with YPD containing 0.02% SDS for 30 min. The numbers at the lower-left corners indicate % of cells with a polarized GFP signal at the tip of daughter cells. n > 200.

To investigate whether SDS also damages the cell wall, we observed chitin that staunches the laser-induced cell wall damage^15^. We found local chitin spots in 84.9 ± 5.5% of cells after SDS treatment (Extended Data Fig. 1a, white arrows), analogous to the phenotype after laser damage (Extended Data Fig. 1b, yellow arrows). Consistently, transmission electron microscopy (TEM) images showed that SDS treatment induced local ingression of the cell wall and plasma membrane structure (Fig. 1c, yellow arrows). These results suggest that SDS breaks both cell wall and plasma membrane and that the damage is local, forming individual spots.

Next, phosphatidylserine (PS) externalization was examined because plasma membrane damage leads to local PS externalization in human cells^17^. Unexpectedly, PS was externalized at the bud scars, former cytokinesis sites marked by circular chitin staining in budding yeast, before the SDS treatment (Fig. 1d, upper panels). In addition to the signal at the bud scars, PS-externalized spots increased after SDS treatment, and the PS spots co-localized with chitin spots (Fig. 1d, lower panels, and Fig. 1e). Moreover, two major repair proteins, Pkc1-GFP and Myo2-GFP, were recruited locally, but not globally, to the cortex after SDS treatment (Extended Data Fig. 1c, yellow arrows). Together, these results demonstrate that SDS induces local plasma membrane and cell wall damage in budding yeast.

**Extended Data Fig. 2:**
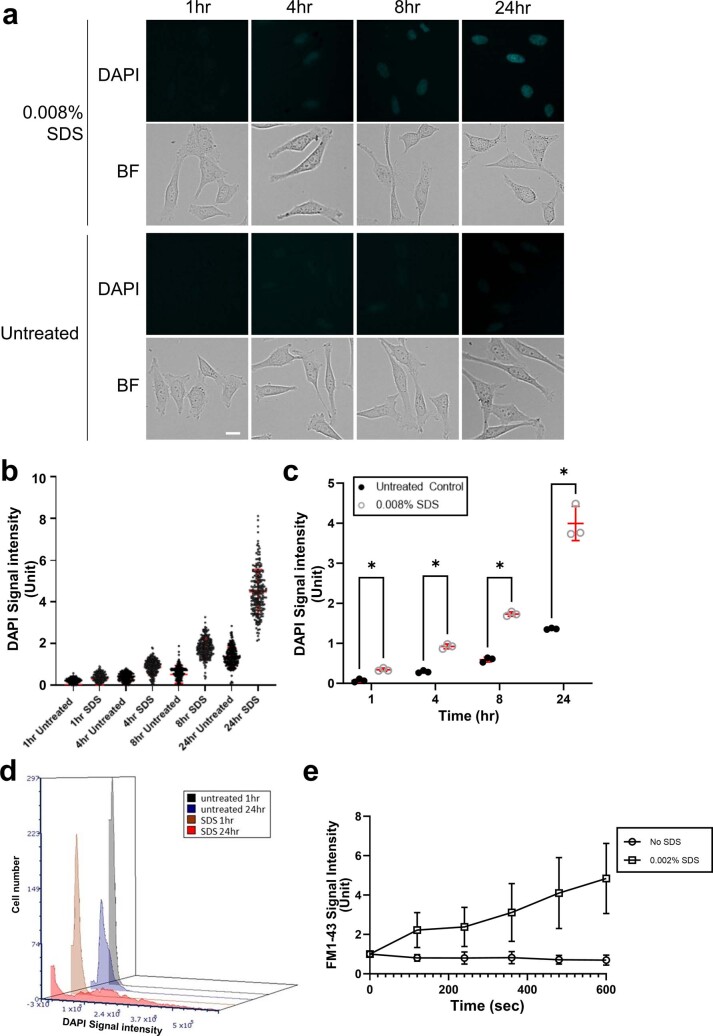
SDS induces plasma membrane damage in human cells. **(a)** Representative images of DAPI penetrated HeLa cells upon 0.008% SDS treatment over time. HeLa cells were cultured in DAPI-containing medium with or without 0.008% SDS. Scale bar, 20 μm. This experiment was independently repeated three times with similar results. **(b)** A representative data set of DAPI penetration assay. DAPI positive cells were counted at indicated time points. Cell number: 1hr Untreated (n = 217), 1hr SDS (n = 208), 4hr Untreated (n = 211), 4hr SDS (n = 219), 8hr Untreated (n = 208), 8hr SDS (n = 212), 24hr Untreated (n = 212), 24hr SDS (n = 216). Data points are shown with Mean(SD). **(c)** Average DAPI intensity of three independent experiments as in (b). Untreated control and 0.008% SDS treatment were significantly different. *P* value: *<0.05, 1hr: 0.001066, 4hr: 0.000079, 8hr: 0.000018, 24hr: 0.000438, using two-sided multiple Welch’s *t*-test with Benjamini, Krieger and Yekutieli correction. Data points are shown with Mean(SD). **(d)** Flow cytometric analyses of penetrated DAPI signals are shown by histogram overlays. WI-38 cells were cultured in DAPI-containing medium with or without SDS (0.0085%). Cell number: untreated 1hr (n = 5029), untreated 24hr (n = 5025), SDS 1hr (n = 5007), SDS 24hr (n = 5192). **(e)** Live-cell imaging was performed using HeLa cells cultured in FM1-43-containing medium (no FBS) with or without 0.002% SDS. The cytosolic FM1-43 signals were quantified (8 cells per each group). n = 10 cells examined over 2 independent experiments. Graphs are shown as Mean(SD). Source data

**Extended Data Fig. 3:**
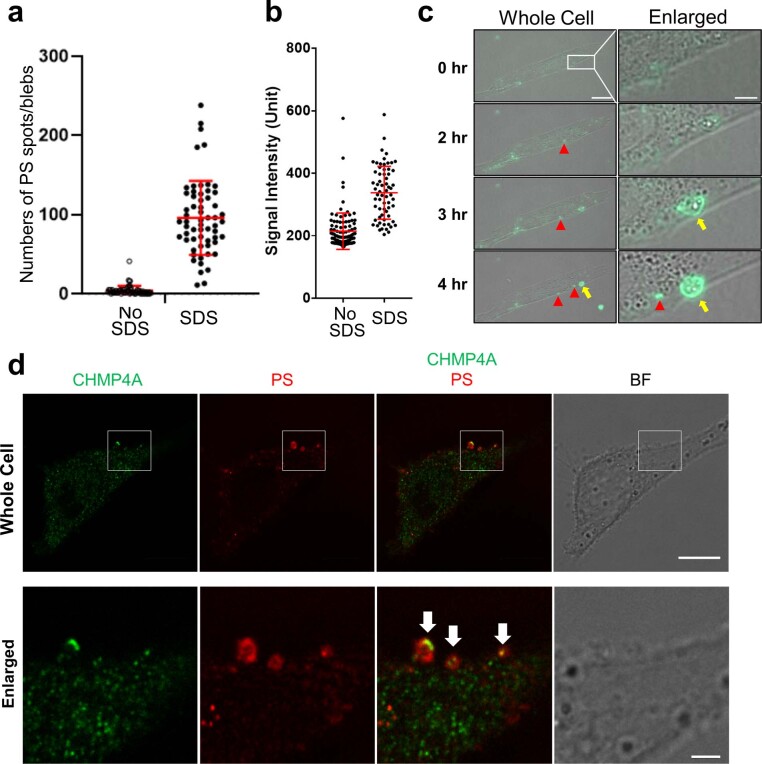
SDS induces local PS externalization that colocalizes with CHMP4A on the plasma membrane of human cells. **(a)** A representative data set of Annexin V spots analyses. The number of Annexin V positive spots per cell was counted. n = 60. Data points are shown with Mean(SD). **(b)** Relative Annexin V signals of entire cells were quantified. Cell number: No SDS (n = 100), SDS 1hr (n = 65). Data points are shown with Mean(SD). **(c)** WI-38 cells were cultured in the medium with 0.008% SDS and pSIVA under the fluorescent microscope. pSIVA binds to externalized PS. Arrow heads, pSIVA positive spots. Yellow arrows, pSIVA positive blebs. The white rectangle region is enlarged in right panels. Scar bar, 20 μm (left), 5 μm (right). Merged images of pSIVA and Bright Field. This experiment was independently repeated two times with similar results. **(d)** HeLa cells were treated with 0.008% SDS for 1 hr. The cells were incubated with Annexin V-Alexa Fluor 647 conjugate, fixed, and stained with CHMP4A antibody. The white rectangle region in upper panels is enlarged in lower panels. Green: CHMP4A, Red: Annexin V, Alexa Fluor 647 conjugate, BF: Bright Field. White arrows indicate the colocalization of green and red signals. Scale bars, 10 μm (upper), 2 μm (lower). This experiment was independently repeated five times with similar results. Source data

To test whether SDS damages the plasma membrane of human cultured cells, we performed the membrane-impermeable fluorescent dye penetration assay. We found that SDS treatment induced the influx of DAPI and FM1-43 into HeLa cells (Fig. 1f,g and Extended Data Fig. 2a–e). In addition, the PS-externalized spots/blebs at the cell periphery increased after SDS treatment in HeLa cells (Fig. 1h,i and Extended Data Fig. 3a,b), analogous to the treatment with other membrane-poring reagents^17^. Live cell imaging confirmed that PS-externalized spots/blebs increased after SDS treatment in WI-38 cells (Extended Data Fig. 3c). Plasma membrane repair protein ESCRT-III (CHMP4A) signals were detected at the PS-externalized spots/blebs (Extended Data Fig. 3d). These results indicate that SDS treatment induces PMD in human cells. Using this simple treatment, we designed a genome-wide screen to identify factors required for plasma membrane repair in budding yeast.

### Identification of the PMD response genes in budding yeast

**Fig. 2:**
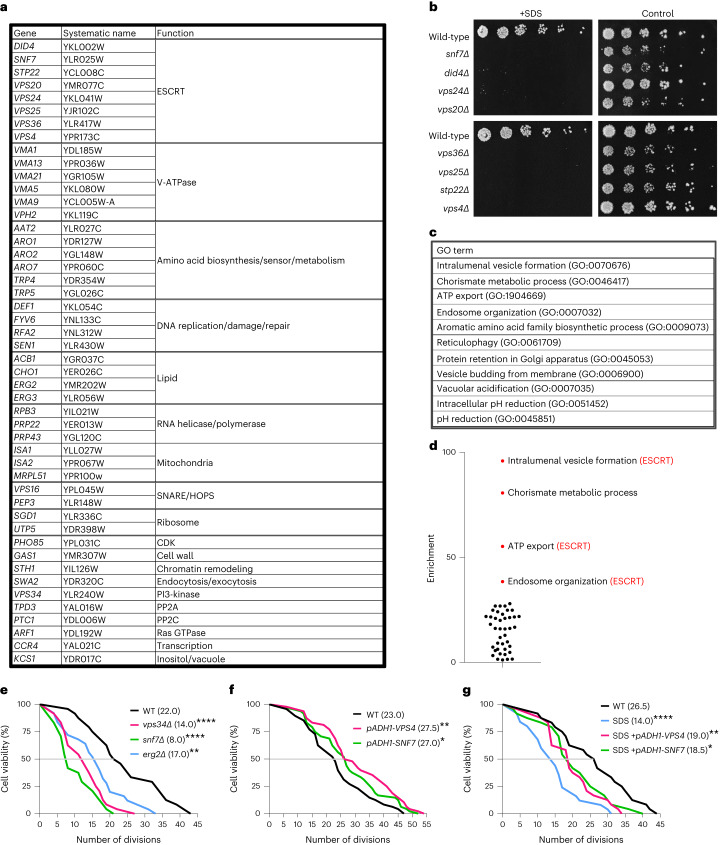
Identification of genes required for PMD response in budding yeast; PMD limits replicative lifespan in budding yeast. **a**, The genes required for the growth in the presence of 0.02% SDS. **b**, ESCRT mutants were spotted on YPD plates (4× dilution series) with or without 0.02% SDS, incubated at 25 °C for 3 d. **c**, Enriched GO terms. See also Supplementary Table 1. **d**, Fold enrichment scores in the GO enrichment analysis were plotted. **e**, Wild-type, *snf7*Δ, *vps34*Δ and *erg2*Δ were subjected to the replicative lifespan measurement. Median values are shown. Cell number: wild-type (*n* = 24), *vps34*Δ (*n* = 24), *snf7*Δ (*n* = 24) and *erg2*Δ (*n* = 25). **f**, Wild-type, *VPS4*-overexpressing or *SNF7*-overexpressing yeast cells were subjected to the replicative lifespan measurement. Median values are shown. Cell number: wild-type (*n* = 48), *VPS4* (*n* = 48), *SNF7* (*n* = 48). **g**, Wild-type, *VPS4*-overexpressing or *SNF7*-overexpressing yeast cells were subjected to the replicative lifespan measurement with or without 0.02% SDS. Median values are shown. Cell number: wild-type (*n* = 24), SDS (*n* = 24), SDS+*VPS4* (*n* = 24) and SDS+*SNF7* (*n* = 25). *P < 0.05, **P < 0.01 and ****P < 0.001, by two-sided Wilcoxon rank-sum test. Exact *P* value: wild-type versus *vps34*Δ: <0.0001, wild-type versus *snf7*Δ: <0.0001 and wild-type versus *erg2*Δ: 0.0029 (**e**); wild-type versus *VPS4*: 0.0087 and wild-type versus *SNF7*: 0.0116 (**f**); wild-type versus SDS: <0.0001, wild-type versus SDS+*VPS4*: 0.0085 and wild-type versus SDS+*SNF7*: 0.0394 (**g**). In **e**–**g**, the median lifespan is indicated as a gray horizontal line. WT, wild-type. Source data

To identify genes essential for the PMD responses, we performed a genome-wide screen using two yeast libraries—non-essential gene deletion library^18^ and DAmP library—in which mRNA levels of essential genes are decreased to 20–50% (ref. ^19^). These two libraries account for 96% of all open reading frames in budding yeast. We identified 48 mutants that were reproducibly sensitive to SDS (Fig. 2a and Supplementary Fig. 1a,b). The screening hits could be manually classified into 19 functional groups. The largest group in the hits was ESCRT with eight genes (Fig. 2b). Gene Ontology (GO) enrichment analysis (http://geneontology.org) revealed that the cellular processes associated with ESCRT were highly enriched (Fig. 2c,d and Supplementary Table 1). Thus, ESCRT’s cellular function is essential for the PMD response in budding yeast, analogous to what was previously shown in higher eukaryotes^1–4,7–11^.

**Extended Data Fig. 4:**
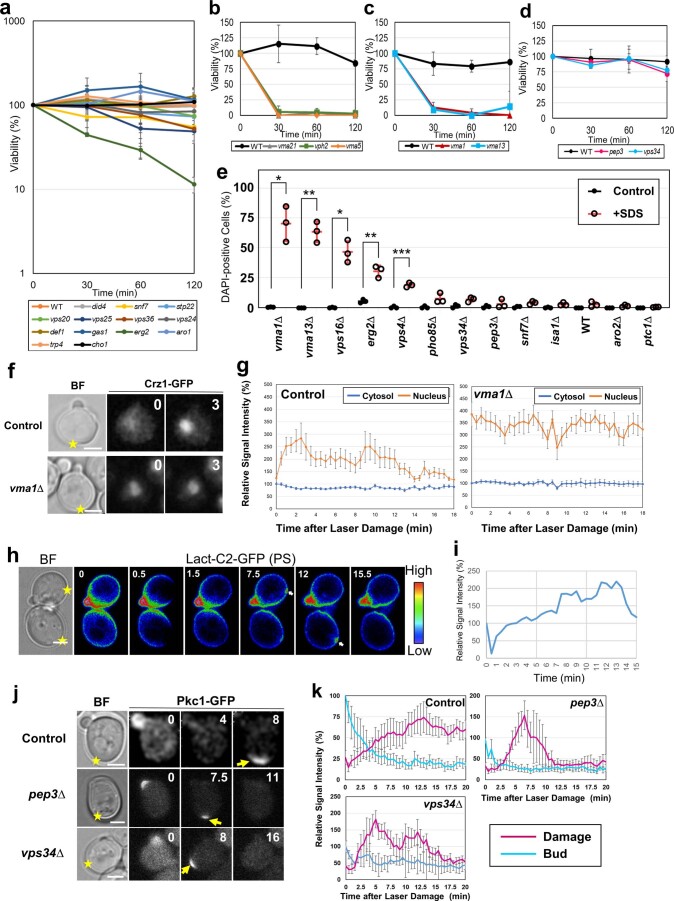
Identification and characterization of factors required for plasma membrane/cell wall damage response in budding yeast. **(a and b)** Yeast gene deletion mutants were cultured in YPD at 25˚C and then incubated with YPD containing 0.02% SDS. Samples were collected at 30 min intervals and spread onto YPD plates. The number of colonies was counted after 2-3 days. Graphs are shown as Mean(SD) of 3 independent experiments. **(c)** Yeast mutants were treated as in (a) except for the usage of YPD (pH 5.0). **(d)** Yeast mutants were treated as in (a). **(e)** Yeast gene deletion mutants were cultured in YPD at 25˚C and then incubated with YPD containing 0.02% SDS for 30 min. Data are shown as Mean(SD) of 3 independent experiments. > 300 cells/sample. For statistical analysis, Control cells vs. +SDS cells were compared. *P* values: *, *p* < 0.05; **, *p* < 0.01; ***, *p* < 0.001, by 2-tailed unpaired Student’s *t*-test. Exact *P* value: *vma1*: 0.0146, *vma13*: 0.0062, *vps16*: 0.0129, *erg2*: 0.0081, *vps4*: 0.0006. **(f)** Wild type (Control) and *vma1∆* expressing Crz1-GFP were cultured under the microscope and subjected to laser damage (yellow stars). Images were taken in 30 sec intervals. The numbers at the upper-right corners indicate the time (min). Scale bar, 2 μm. **(g)** Quantification of (f). Data are shown as Mean(SEM) of 8 independent experiments. **(h)** Wild type yeast expressing Lact-C2-GFP were cultured under the microscope and subjected to the laser damage as in (f). Relative signal intensity is shown in rainbow pseudocolor. The numbers at the left-top corners indicate the time (min). Arrows: the GFP signal accumulation at the laser damage sites. Scale bar, 2 μm. **(i)** Quantification of (h). Mean of 10 independent experiments. **(j)** Wild type (Control), *pep3∆* and *vps34∆* expressing Pkc1-GFP were cultured under the microscope and subjected to laser damage as in (f). Scale bar, 2 μm. **(k)** Quantification of (j). Control (n = 5), pep3 (n = 3), vps34 (n = 4) independent cells were examined. Mean(SEM). Source data

We performed characterization of the screening hits (Extended Data Fig. 4a–k and Supplementary Fig. 2). The details are explained in the Supplementary Text. In short, ESCRT mutants (*did4*Δ, *snf7*Δ, *stp22*Δ, *vps20*Δ, *vps25*Δ, *vps36*Δ and *vps24*Δ) survived for at least 2 h in the SDS-containing medium. In contrast, V-ATPase mutants (*vma21*Δ, *vph2*Δ, *vma5*Δ, *vma1*Δ and *vma13*Δ) lost their viability after 30-min incubation in the medium containing SDS (Extended Data Fig. 4a–d). V-ATPase produces a proton gradient across the vacuolar membrane, enabling Ca^2+^ uptake into the vacuole; the mutants lacking functional V-ATPase show high cytoplasmic Ca^2+^ levels. Because Ca^2+^ influx at the damage site is essential for membrane resealing in higher eukaryotes^6,7^, SDS sensitivity in V-ATPase mutants may be explained by the high cytosolic Ca^2+^ concentration in V-ATPase mutants, preventing membrane resealing.

Together with further characterization described in the Supplementary Text, here we reveal four cellular processes during plasma membrane/cell wall damage response in budding yeast: (1) V-ATPase-dependent prevention of immediate cell death, (2) Crz1 nuclear import, (3) PS recruitment to the damage site and (4) Pep3-dependent and Vps34-dependent retention of Pkc1 at the damage site (Supplementary Fig. 2).

### PMD limits the replicative lifespan of budding yeast

**Extended Data Fig. 5:**
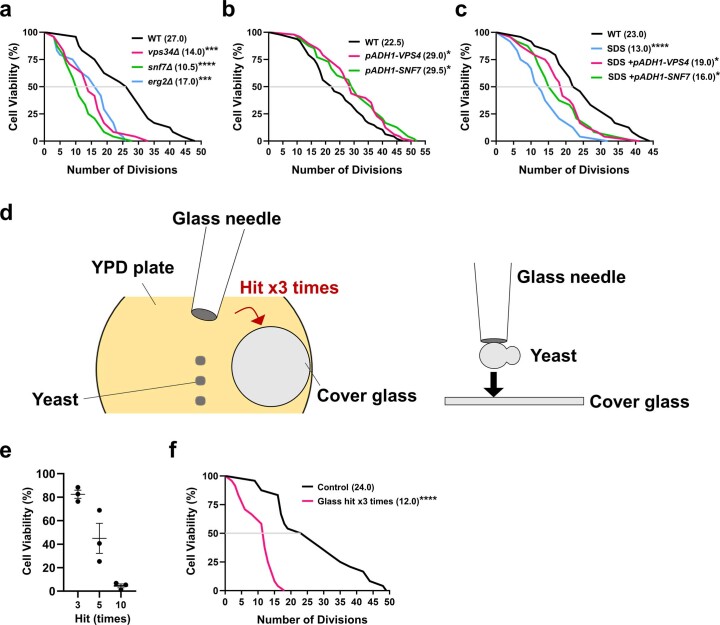
plasma membrane damage limits replicative lifespan in budding yeast. **(a-c)** Yeast replicative life span was measured with another biological replicate, and results were consistent with Fig. 2 (e-g). Cell number: (a) Wild type (n = 24), vps34Δ (n = 24), snf7Δ (n = 24), erg2Δ (n = 24). (b) Wild type (n = 48), VPS4 (n = 48), SNF7 (n = 48). (c) Wild type (n = 24), SDS (n = 24), SDS+VPS4 (n = 24), SDS+SNF7 (n = 24). *P* value: *<0.05, **<0.01, ****<0.001 by two-sided Wilcoxon Rank-sum test. Exact *P* value: (a) WT vs. *vps34*Δ: 0.0005, WT vs. *snf7*Δ: <0.0001, WT vs. *erg2*Δ: 0.0008. (b) WT vs. *VPS4*: 0.0209, WT vs. *SNF7*: 0.0465. (c) WT vs. SDS: <0.0001, WT vs. SDS+*VPS4*: 0.0473, WT vs. SDS+*SNF7*: 0.0158. **(d)** A schematic image of mechanical damage induced by a glass needle and a glass coverslip. **(e)** The viability of the yeast cells was measured. Mean(SD) of 3 biological replicates. n = 40. **(f)** WT yeast cells were subjected to the replicative life span measurement with or without mechanical damage. Median values are shown. Cell number: Wild type (n = 24), Glass (n = 24). *P* value: ****<0.001, exact value: <0.0001, by two-sided Wilcoxon Rank-sum test. Source data

The gene sets required for the cellular/subcellular processes after plasma membrane damage should be enriched in our hits. To identify the reported phenotype enriched for our hits, we performed model organism Phenotype Enrichment Analysis (*mod*PhEA)^20^ (http://evol.nhri.org.tw/phenome2). We found that the genes associated with the phenotype ‘replicative lifespan’ were significantly enriched (Supplementary Tables 2 and 3). Motivated by this finding, we performed a replicative lifespan analysis^21^ using our screening hits. All three mutants tested (*snf7*Δ, *vps34*Δ and *erg2*Δ) showed no growth in the presence of SDS, which is consistent with our screening strategy (Supplementary Fig. 1). *snf7*Δ, *vps34*Δ and *erg2*Δ cells showed markedly shorter replicative lifespan, in the absence of SDS, compared to wild-type cells (Fig. 2e and Extended Data Fig. 5a), further suggesting a link between the PMD responses and replicative lifespan regulation. Consistent with these results, overexpression of the ESCRT activator AAA-ATPase *VPS4* and overexpression of ESCRT-III *SNF7* extended the replicative lifespan (Fig. 2f and Extended Data Fig. 5b). Next, we examined the replicative lifespan of wild-type cells, *VPS4*-overexpressing cells and *SNF7*-overexpressing cells in the presence or absence of SDS. We found that SDS significantly shortened the replicative lifespan of wild-type yeast cells and that overexpression of *VPS4*/*SNF7* partially suppressed it (Fig. 2g and Extended Data Fig. 5c). To test whether the mechanical injury shortens yeast replicative lifespan, we introduced a mechanical stress, where yeast cells were smashed between a glass needle and a glass coverslip. We found that this treatment also shortened the replicative lifespan of wild-type cells (Extended Data Fig. 5d–f). These results suggest that PMD limits the replicative lifespan of budding yeast and that ESCRT’s function is critical for the regulation of replicative lifespan.

### Transient PMD induces senescence in normal human fibroblasts

**Fig. 3:**
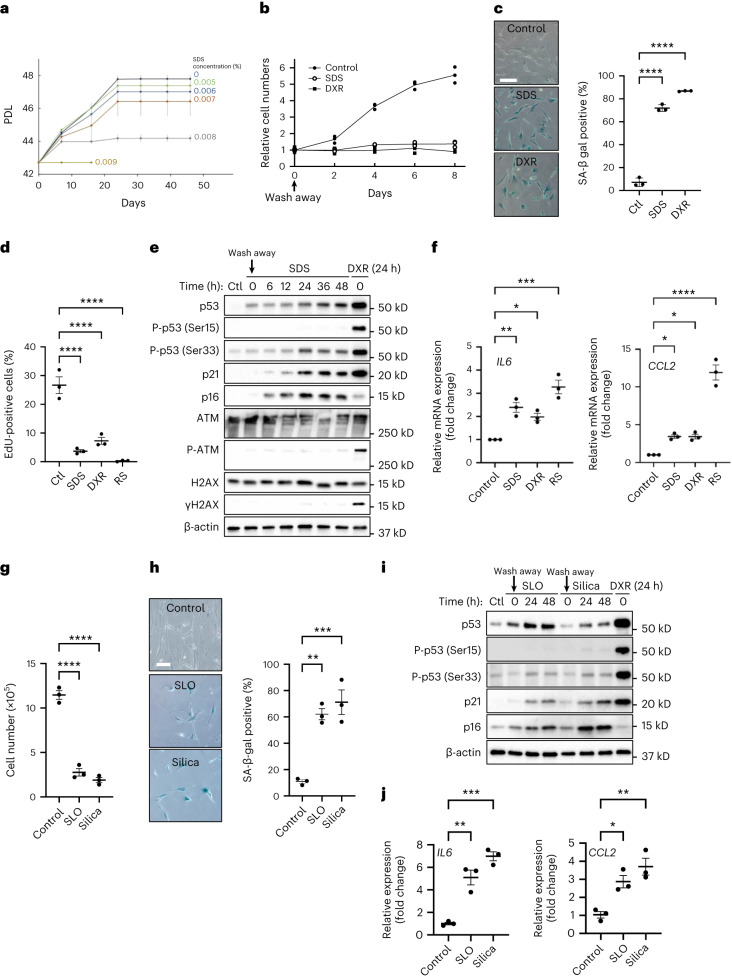
Transient PMD induces premature senescence in normal human fibroblasts. **a**, PDLs of WI-38 cells. WI-38 cells were cultured continuously in the medium containing SDS (0.005–0.009%, indicated on the right). **b**–**f**, WI-38 cells were incubated with 0.007% SDS or 250 nM DXR for 24 h, washed and released into fresh medium. **b**, Relative cell numbers are indicated. **c**, SA-β-gal-positive cells were detected using the cells 10 d after wash away. Scale bar, 200 μm. *n* > 100 cells. ****P < 0.001: control (Ctl) versus SDS: <0.0001 and Ctl versus DXR: <0.0001, by one-way ANOVA with Dunnettʼs test. **d**, WI-38 young cells (Ctl) and senescent cells (SDS, DXR and RS) were labeled with 10 μM EdU for 24 h. EdU–Alexa Fluor 647 signals and Hoechst 33342 signals were obtained, and the ratio of EdU-incorporated cells was calculated. *n* > 200 cells. ****P < 0.001: Ctl versus SDS: <0.0001; Ctl versus DXR: <0.0001; Ctl versus RS: <0.0001, by one-way ANOVA with Dunnettʼs test. **e**, Western blotting using the cell lysates of WI-38 cells treated with SDS or DXR. The cells after 24-h treatment were collected at the indicated times after wash away. **f**, qPCR analysis of SASP genes (*IL6* and *CCL2*) in senescent WI-38 cells. RNA was isolated from WI-38 untreated cells (Ctl), 5 d after SDS or DXR wash away and RS. *P <0.05, **P < 0.01 and ***P < 0.001, by one-way ANOVA with Dunnettʼs test. Exact *P* value: *IL6*_Control versus SDS: 0.0045, *IL6*_Control versus DXR: 0.0309, *IL6*_Control versus RS: 0.0002, *CCL2*_Control versus SDS: 0.0318, *CCL2*_Control versus DXR: 0.033, *CCL2*_Control versus RS: <0.0001. **g**–**j**, WI-38 cells were incubated with 200 ng ml^−1^ SLO, 125 μg ml^−1^ silica (diameter, 0.8 μm) (Silica) or 250 nM DXR for 24 h, washed and released into fresh medium. **g**, Cell number was counted 10 d after wash away. ****P < 0.001: Ctl versus SLO: <0.0001 and Ctl versus Silica: <0.0001, by one-way ANOVA with Dunnettʼs test. **h**, SA-β-gal-positive cells were detected 10 d after wash away. Scale bar, 50 μm. Graphs show quantification of SA-β-gal-positive cells (*n* > 100 cells). **P < 0.01 and ***P < 0.005: Ctl versus SLO: 0.0022 and Ctl versus Silica: 0.0009, by one-way ANOVA with Dunnettʼs test. **i**, Western blotting using the cell lysates of WI-38 cells treated with SLO or Silica for 24 h. Cells were collected at the indicated times after wash away. **j**, qPCR analysis of *IL6* and *CCL2* gene expression. RNA was isolated 5 d after wash away. *P < 0.05, **P < 0.01 and ***P < 0.001, by one-way ANOVA with Dunnettʼs test. Exact *P* value: *IL6*_Control versus SLO: 0.0012, *IL6*_Control versus Silica: 0.0001, *CCL2*_Control versus SLO: 0.0179 and *CCL2*_Control versus Silica: 0.0031. Data in **c**, **d** and **f**–**j** are presented as mean (horizontal bars) ± s.d. (whiskers) of at least three biological replicates. Source data

**Extended Data Fig. 6:**
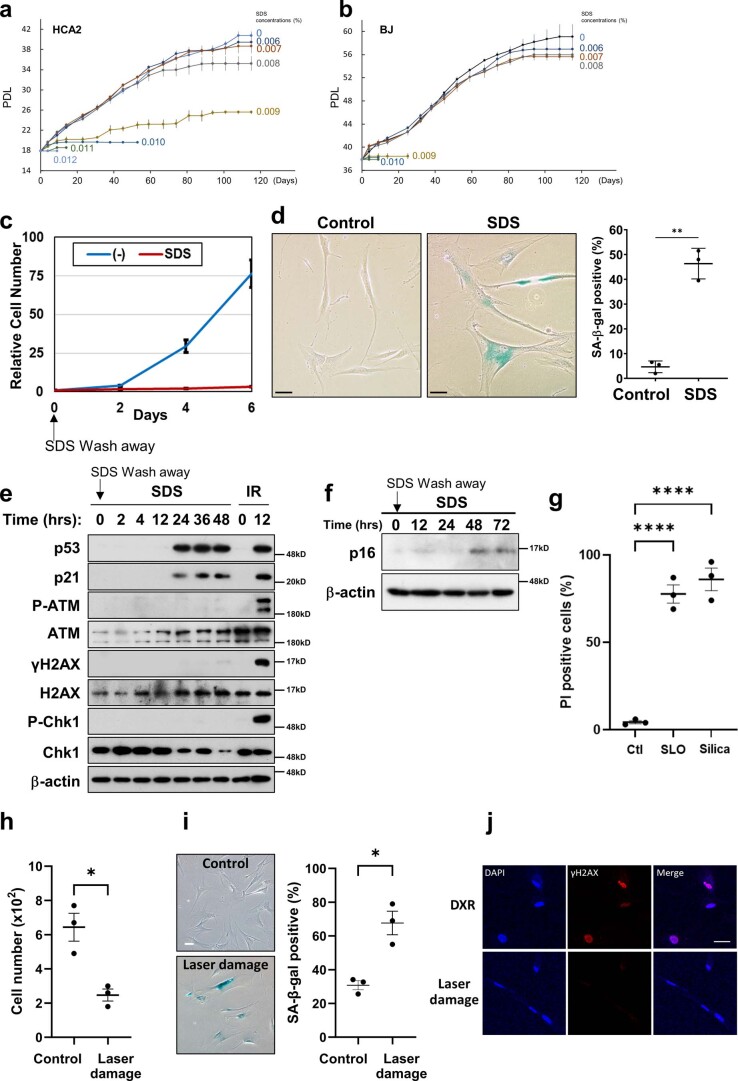
Transient plasma membrane damage induces premature senescence in normal human fibroblasts. **(a and b)** PDL of human normal fibroblasts with continuous SDS treatment. Human normal fibroblasts (a: HCA2, b: BJ) were cultured with or without SDS (0.006–0.012%; indicated on the right). **(c)** Relative cell numbers of HCA2 cells after the transient (24 hours) SDS treatment. More than 1x10^5^ cells were quantified. HCA2 cells were incubated in the medium with (SDS) or without (-) 0.01% SDS for 24 hours, washed, and released into fresh medium. Data are shown as Mean(SD) of 3 independent experiments. **(d)** HCA2 cells were treated with 0.01% SDS for 24 hours, washed, and released into fresh medium. SA-β-gal positive cells were detected using the cells 6 days after SDS wash away. Scale bar, 50 μm. Graphs show quantification of SA-β-gal positive cells (n > 100). Data are shown as Mean(SD) of >3 independent experiments. *P* value: **<0.01, exact value: 0.0032, by 2-tailed unpaired Student’s *t*-test. **(e and f)** Western blotting using cell lysates of HCA2 cells treated with 0.01% SDS for 24 hours or 10 Gy IR (Irradiation, control). This experiment was independently repeated three times with similar results. **(g)** PI penetration assay was performed with 24 hours treatment of 200 ng/ml streptolysin O (SLO), 125 μg/ml silica (φ = 0.8 μm) (Silica). n > 100 cells. *P* value: ****<0.001, Control vs. SLO: <0.0001, Control vs. Silica: <0.0001, by One-way ANNOVA, Dunnett. **(h)** Cell number of WI-38 cells in 35 mm glass bottom dish was counted 10 days after laser irradiation. *P* value: *<0.05, exact value: 0.026, by 2-tailed unpaired Student’s *t*-test. **(i)** SA-β-gal positive cells were detected 16 days after laser irradiation. Scale bar, 50 μm. Graphs show quantification of SA-β-gal positive cells. n > 100 cells. *P* value: *<0.05, exact value: 0.0227, by 2-tailed unpaired Student’s *t*-test. **(j)** WI-38 cells just after laser irradiation or cells at 24 hours after DXR treatment were stained with γH2AX (red) and with DAPI (blue). Scale bar, 50 μm. Graphs are shown as Mean(SD) of 3 biological replicates. Source data

To test the possibility that PMD induces premature senescence in human cells, we performed a long-term culture of normal human fibroblasts (WI-38, HCA2 and BJ) in the presence or absence of PMD. Indeed, cell proliferation was inhibited in an SDS concentration-dependent manner (Fig. 3a, Extended Data Fig. 6a,b and Supplementary Fig. 3).

To minimize side effects, we transiently treated normal human fibroblasts with SDS. WI-38 and HCA2 cells were incubated with SDS-containing media for 24 h, washed with medium and then cultured in fresh medium. We found that cell proliferation was inhibited after the treatment, and the proportion of senescence-associated β-galactosidase (SA-β-gal)-positive cells was increased after 10 d (Fig. 3b,c and Extended Data Fig. 6c,d). 5-Ethynyl-2′-deoxyuridine (EdU) incorporation was attenuated in the cells 16 d after the PMD treatment, indicating that DNA replication halted (Fig. 3d). Consistently, the protein levels of p53 (Fig. 3e and Extended Data Fig. 6e), an essential senescence regulator in mammalian cells^22,23^, and its target, p21, increased in cells treated with SDS as early as 24 h after SDS wash away (Fig. 3e and Extended Data Fig. 6e). p53 is known to be phosphorylated at multiple sites by several different kinases. For example, DNA damage response induces p53 phosphorylation at Ser15 (refs. ^24–26^), and osmotic shock or UV irradiation (IR) induces p53 phosphorylation at Ser33 (refs. ^27,28^). Notably, p53 phosphorylation at Ser33, but not Ser15, increased 24 h after SDS treatment (Fig. 3e). We found that the levels of p16 also increased 48 h after the SDS wash away (Fig. 3e and Extended Data Fig. 6f). The mRNA levels of senescence-associated secretory phenotype (SASP) factors *IL6* and *CCL2* were upregulated in SDS-treated cells, analogous to replicative senescent and DNA damage-treated cells (Fig. 3f). Together, these results suggest that PMD induced by SDS promotes cellular senescence in normal human fibroblasts.

To generalize our finding, we tested other plasma membrane-damaging stimuli, including SLO, silica and laser damage. Penetration assay using a barely membrane-permeable dye, propidium iodide (PI), confirmed successful PMD induction after SLO treatment and silica treatment (Extended Data Fig. 6g). Consistently, SLO treatment and silica treatment induced senescent cell features, including proliferation arrest, increase in the proportion of SA-β-gal-positive cells and upregulation of p53, p21 and p16 protein levels as well as the SASP factors *IL6* and *CCL2* mRNA levels (Fig. 3g–j). Similar to SDS-dependent PMD, p53 phosphorylation at Ser33, but not at Ser15, was increased 24 h after SLO and silica treatment. Moreover, laser damage at the plasma membrane also induced senescent cell features, including proliferation arrest, enlarged cell morphology and increase in the proportion of SA-β-gal-positive cells (Extended Data Fig. 6h,i). After the laser damage, the cells did not show detectable DNA damage marked with γH2AX immunostaining (Extended Data Fig. 6j). Together, these results suggest that PMD triggers premature senescence in normal human fibroblasts.

### PMD-dependent senescence is suppressed by CHMP4B

**Fig. 4:**
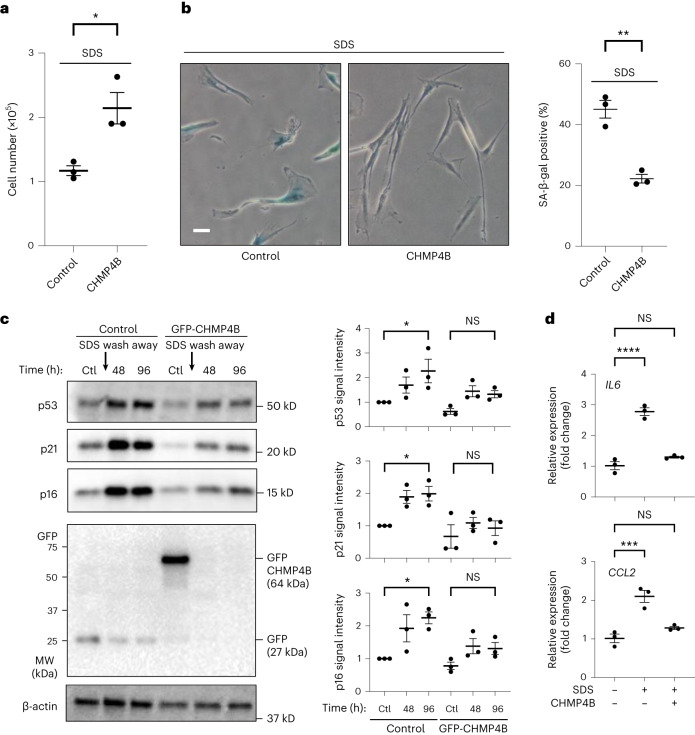
Transient overexpression of ESCRT-III (CHMP4B) suppressed PMD-dependent senescence. **a**–**d**, A control plasmid (GFP) or a plasmid encoding ESCRT-III (GFP-CHMP4B) was overexpressed in WI-38 cells. The cells were treated with 0.007% SDS for 24 h, washed and released into fresh medium. **a**, Cell number in a 6-cm-diameter dish was counted 10 d after SDS wash away. *P < 0.05, exact value: 0.0468, by two-tailed unpaired Student’s *t*-test. **b**, SA-β-gal-positive cells were detected 7 d after SDS wash away. Scale bar, 50 μm. Graphs show quantification of SA-β-gal-positive cells (*n* > 100 cells). **P < 0.01, exact value: 0.0067, by two-tailed unpaired Student’s *t*-test. **c**, Western blotting using the cell lysates of WI-38 cells treated with SDS for 24 h that were collected at the indicated times after wash away. GFP (control) and GFP-CHMP4B expression was confirmed by anti-GFP blot. Relative signal intensities are quantified. *P < 0.05, by one-way ANOVA with Dunnettʼs test. Exact *P* value: p53_Ctl versus 96 h in control (Ctl) cells: 0.0491; p53_Ctl versus 96 h in CHMP4B-expressed cells: 0.4648; p21_Ctl versus 96 h in Ctl cells: 0.0477; p21_Ctl versus 96 h in CHMP4B-expressed cells: 0.5864; p16_Ctl versus 96 h in Ctl cells: 0.0195; p16_Ctl versus 96 h in CHMP4B-expressed cells: 0.5793. **d**, qPCR analysis of *IL6* and *CCL2* gene expressions. RNA was isolated 5 d after wash away. ***P < 0.005 and ****P < 0.001, by one-way ANOVA with Dunnettʼs test. Exact *P* value: *IL6*_Ctl versus SDS in Ctl cells: <0.0001; *IL6*_Ctl versus SDS in CHMP4B-expressed cells: 0.1843; *CCL2*_Ctl versus SDS in Ctl cells: <0.0009; *CCL2*_Ctl versus SDS in CHMP4B-expressed cells: 0.2306. Data in **a**–**d** are presented as mean (horizontal bars) ± s.d. (whiskers) of three biological replicates. NS, not significant. Source data

To test whether upregulation of plasma membrane repair suppresses PMD-Sen, we examined whether transient overexpression of ESCRT-III (CHMP4B) suppresses PMD-Sen. We transfected the plasmid harboring GFP-CHMP4B into WI-38 cells, and the cells were treated with SDS as described above. We found that overexpression of CHMP4B, only transient overexpression during the SDS treatment, bypassed SDS-induced proliferation arrest and decreased the proportion of SA-β-gal-positive cells (Fig. 4a,b). Consistent with these results, SDS-induced upregulation of p53, p21 and p16 protein levels was attenuated in the CHMP4B-expressing cells (Fig. 4c). SASP factor upregulation was suppressed as well (Fig. 4d). Together, these results are consistent with our understanding that PMD induces cellular senescence and that upregulation of plasma membrane repair suppresses it.

### PMD induces senescence via p53 in normal human fibroblasts

**Fig. 5:**
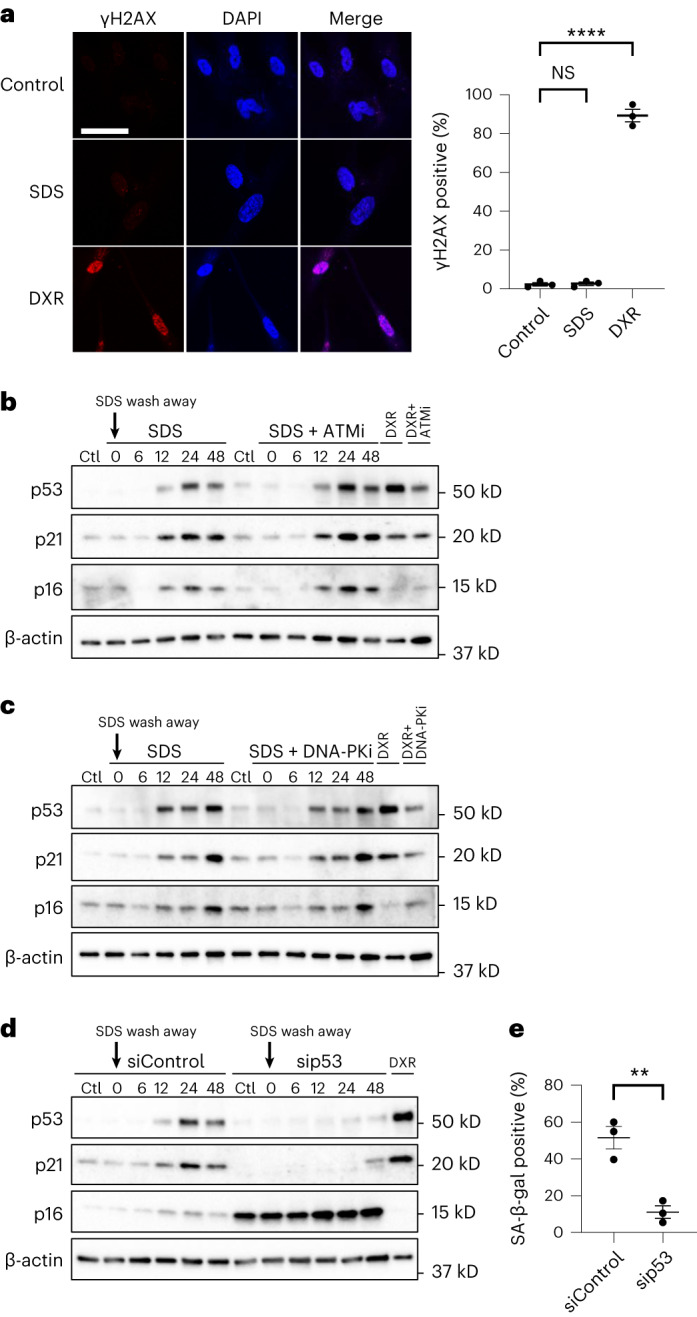
PMD-dependent senescence requires p53. **a**–**e**, WI-38 cells were treated with 0.007% SDS or 250 nM DXR combined with indicated additional treatment for 24 h, washed and released into fresh medium. **a**, γH2AX staining. WI-38 cells at 24 h after SDS or DXR wash away and untreated cells (control) were stained with γH2AX (red) and with DAPI (blue). Scale bar, 50 μm. Graphs show quantification of γH2AX-positive cells (*n* > 100 cells). ****P < 0.001, control versus SDS: 0.9896 and control versus DXR: <0.0001, by one-way ANOVA with Dunnettʼs test. **b**–**d**, Western blotting using cell lysates of WI-38 cells treated with SDS with or without ATM inhibitor KU-55944 (10 μM) (**b**), DNA-PK inhibitor (10 μM) (**c**) and p53 siRNA (**d**). **e**, SA-β-gal-positive cells were counted on 10 d after wash away. WI-38 cells were treated with p53 siRNA (sip53) or control scramble siRNA (siControl). **P < 0.01, exact value: 0.0091, by two-tailed unpaired Student’s *t*-test. Data in **a** and **e** are presented as mean (horizontal bars) ± s.d. (whiskers) of three biological replicates. NS, not significant. Source data

**Extended Data Fig. 7:**
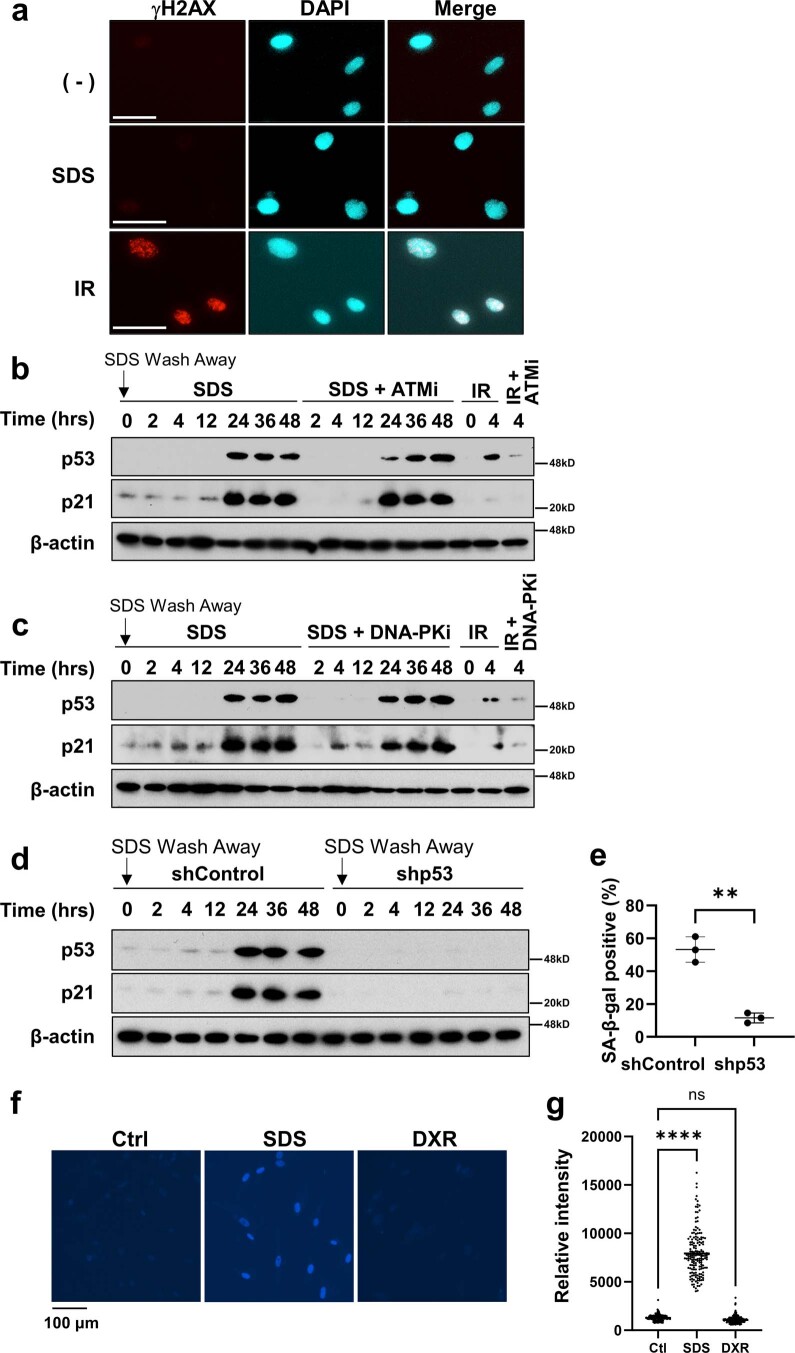
PMD-Sen induction requires p53 and doxorubicin does not induce plasma membrane damage. **(a)** HCA2 cells at 24 hours after SDS wash away (SDS), cells at 4 hours after 10 Gy Irradiation treatment (IR), and untreated cells (−) were stained with γH2AX (red) and with DAPI (blue). Scale bar, 50 μm. This experiment was independently repeated three times with similar results. **(b-d)** Western blotting of p53 and p21. This experiment was independently repeated three times with similar results. **(b and c)** HCA2 cells were treated as in Fig. 3f with or without (b) ATM inhibitor KU-55944 (10 μM) or (c) DNA-PK inhibitor (10 μM). **(d)** Cells expressing doxycycline-inducible control shRNA (shControl) or p53 shRNA (shp53) with doxycycline (1 μg/ml) were treated with 0.01% SDS for 24 hours. After SDS wash away, cells lysates prepared at the indicated times were subjected to immunoblotting using the antibodies indicated. **(e)** SA-β-gal positive cells were counted on 6 days after SDS wash away. n = 200 cells examined over 3 biologically independent experiments. Data are shown as Mean(SD). *P* value: **<0.01, exact value: 0.0056, by 2-tailed unpaired Student’s *t*-test. **(f)** Representative images of DAPI penetration upon 0.008% SDS treatment (SDS) and 250 nM DXR for 24 hr. WI-38 cells were cultured in DAPI-containing medium with or without 0.008% SDS or 250 nM DXR. Scale bar, 20 μm. **(g)** A representative data set of DAPI penetration assay. DAPI positive cells were counted at indicated time points. Cell number: Control (n = 248), SDS (n = 166), DXR (n = 210). ****; *p* < 0.0001, Ctl vs. SDS: <0.0001, Ctl vs. DXR: 0.1928, by one-way ANOVA. ns: not significant. Scale bar, 100 μm. Data are shown as Mean(SEM) Source data

The best-characterized cellular senescence mechanism is the DNA damage response pathway-dependent upregulation of p53–p21, which is activated after many senescence-inducing stimuli, including telomere shortening, DNA damage and oncogene activation^29,30^. We tested whether this pathway is activated after PMD. SDS treatment increased the protein levels of p53 and p21. However, the DNA damage markers γH2AX and phospho-ATM did not increase at least up to 48 h after SDS wash away (Fig. 3e and Extended Data Fig. 6e). Consistent with these results, γH2AX signal was undetectable in the cells at 24 h after the SDS wash away (Fig. 5a and Extended Data Fig. 7a). These results suggest that the DNA damage response pathway is dispensable for PMD-dependent upregulation of p53–p21.

To test this possibility further, WI-38 and HCA2 cells were treated with the inhibitors of ATM and DNA-PKs (Fig. 5b,c and Extended Data Fig. 7b,c). Both inhibitors did not alter the SDS-dependent upregulation of p53 and p21. In contrast, p53 knockdown abolished p21 induction (Fig. 5d and Extended Data Fig. 7d) and significantly decreased the proportion of SA-β-gal-positive cells after SDS wash away (Fig. 5e and Extended Data Fig. 7e). p16 level was increased by p53 knockdown, as previously described^31^. Complementarily, DNA damage-inducing doxorubicin (DXR) treatment did not induce detectable plasma membrane rupture in the DAPI penetration assay (Extended Data Fig. 7f,g). Considering these results, we conclude that PMD induces cellular senescence via p53; however, the upregulation of p53 is regulated by a mechanism independent of the best-characterized senescence pathway: the DNA damage response pathway.

### Ca^2+^ influx mediates PMD-dependent senescence

**Fig. 6:**
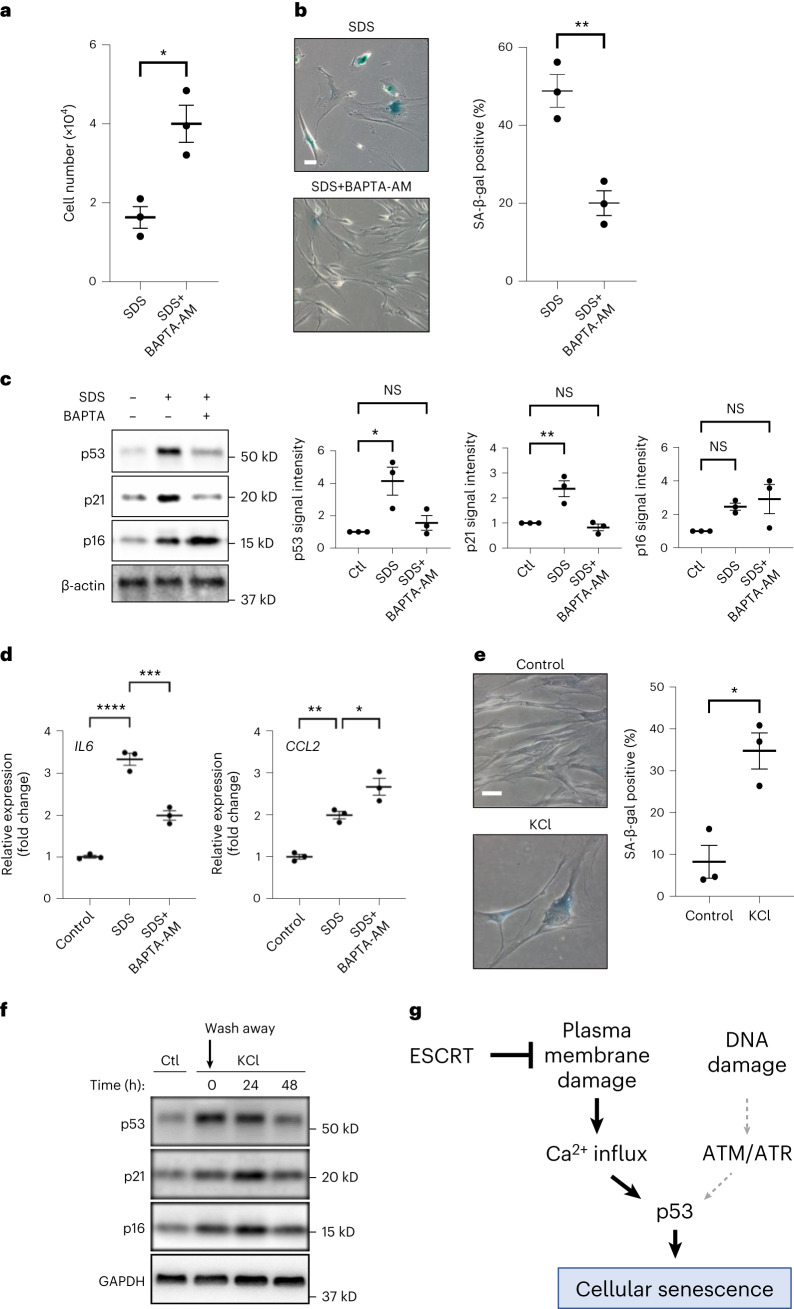
Ca^2+^ influx is necessary to induce PMD-dependent senescence, and KCl-dependent Ca^2+^ influx is sufficient to induce cellular senescence. **a**–**d**, WI-38 cells were treated with 0.007% SDS with or without Ca^2+^ chelator BAPTA-AM (1 μM) for 24 h, washed and released into fresh medium. **a**, Cell number in a 6-cm-diameter dish was counted 10 d after SDS wash away. *P < 0.05, exact value: 0.0193, by two-tailed unpaired Student’s *t*-test. **b**, SA-β-gal-positive cells were detected 10 d after SDS wash away. Scale bar, 50 μm. Graphs show quantification of SA-β-gal-positive cells (*n* > 100 cells). **P < 0.01, exact value: 0.0066, by two-tailed unpaired Student’s *t*-test. **c**, Western blotting. The cells were collected at 6 d after wash away. Relative signal intensities are shown. *P < 0.05 and **P < 0.01, by one-way ANOVA with Dunnettʼs test. Exact *P* value: p53_Ctl versus SDS: 0.0179; p53_Ctl versus SDS+BAPTA-AM: 0.7709; p21_Ctl versus SDS: 0.0067; p21_Ctl versus SDS+BAPTA-AM: 0.8175; p16_Ctl versus SDS: 0.1591; p16_Ctl versus SDS+BAPTA-AM: 0.0696. **d**, qPCR analysis of *IL6* and *CCL2* gene expressions. RNA was isolated 5 d after wash away. *P < 0.05, **P < 0.01, ***P < 0.005 and ****P < 0.001, by one-way ANOVA with Dunnettʼs test. Exact *P* value: *IL6*_Ctl versus SDS: <0.0001, *IL6*_Ctl versus SDS+BAPTA-AM: 0.0003; *CCL2*_Ctl versus SDS: 0.0043; *CCL2*_Ctl versus SDS+BAPTA-AM: 0.0254. **e**,**f**, WI-38 cells were treated with 75 mM KCl for 24 h, washed and released into fresh medium. **e**, SA-β-gal-positive cells were detected using the cells 7 d after KCl wash away. Scale bar, 50 μm. Graphs show quantification of SA-β-gal-positive cells (*n* > 100 cells). *P < 0.05, exact value: 0.0107, by two-tailed unpaired Student’s *t*-test. **f**, Western blotting using cell lysates collected at indicated timepoints. Data in **a**–**e** are presented as mean (horizontal bars) ± s.d. (whiskers) of three biological replicates. **g**, Summary of the PMD-Sen mechanism. Ctl, control; NS, not significant; ATM, ataxia telangiectasia mutated; ATR, ataxia telangiectasia and Rad3-related protein. Source data

**Extended Data Fig. 8:**
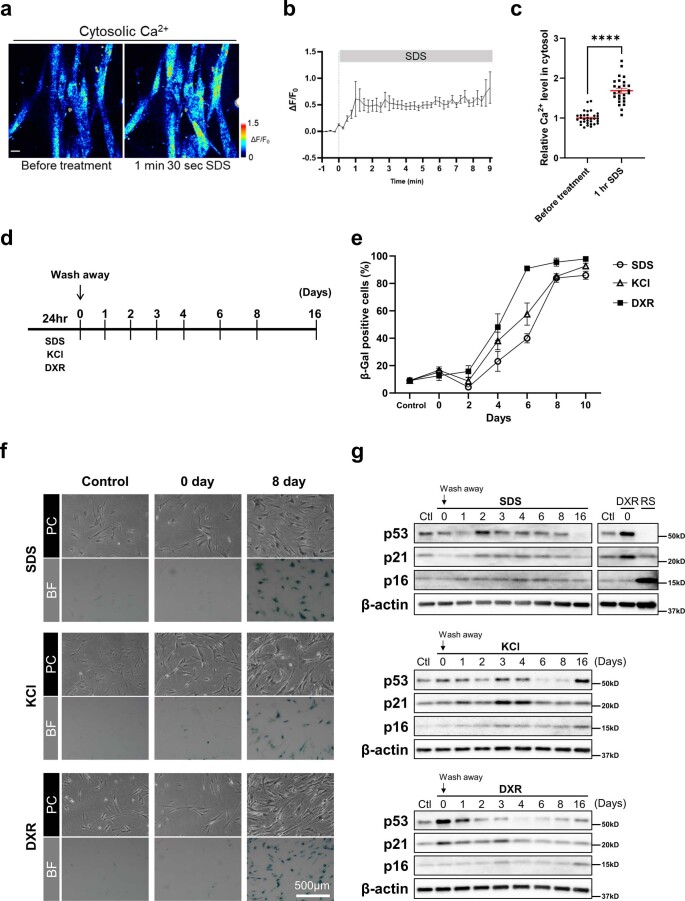
Cytosolic Ca^2+^ levels are increased after SDS treatment and Plasma membrane damage and Ca^2+^-influx induce cellular senescence. **(a)** Representative ratiometric images of cytosolic Ca^2+^ (Cal Red R525/650) in WI-38 cells before or 1.5 min after addition of 0.0095% SDS. Fluorescence intensity was shown as a rainbow pseudocolor. Scale bar, 20 μm. **(b)** Cal Red R525/650 signals in WI-38 cells after addition of 0.0095%. n = 8. **(c)** Relative cytosolic Ca^2+^ levels in cells treated with 0.0095% SDS for one hour (n = 26 cells) compared with untreated cells (n = 32 cells) over 3 independent experiments. *P* value: ****<0.0001, by 2-tailed unpaired Student’s *t*-test. Mean(SEM). **(d)** The experimental design. **(e and f)** SA-β-gal positive cells were detected using WI-38 cells treated with 0.008% SDS, 75 mM KCl, and 250 nM DXR for 24 hours. The cells were washed and stained at the indicated time points. PC, Phase Contrast. Scale bar, 500 μm. n > 80 cells. The precise cell numbers are shown in Statistical Source Data file. 3 biological replicates. Mean(SD) is shown. **(g)** Western blotting using cell lysates of the WI-38 cells used in (e) and (f). This experiment was independently repeated three times with similar results. Source data

Ca^2+^ influx is one of the earliest responses after PMD in all cell types^1,2^, and Ca^2+^ signaling is involved in cellular senescence^32^. To test whether Ca^2+^ influx mediates cellular senescence after PMD, first, we tested cytosolic Ca^2+^ levels after SDS treatment. We found that cytosolic Ca^2+^ levels increased at 1 min after the SDS addition (Extended Data Fig. 8a–c). Next, we examined whether BAPTA-AM, cell-permeable cytosolic Ca^2+^ chelator, suppresses PMD-Sen. We administered BAPTA-AM in combination with SDS treatment and found that BAPTA-AM markedly suppressed senescent cell features, including SDS-induced proliferation arrest, SA-β-gal positivity, p53–p21 upregulation and *IL6* mRNA upregulation (Fig. 6a–d). Intriguingly, SDS-dependent upregulation of p16 and *CCL2* were not suppressed by BAPTA-AM (Fig. 6c,d). These results suggest that PMD-Sen is regulated by at least two pathways: Ca^2+^ influx-dependent and influx-independent pathways. In line with these results, enforced plasma membrane Ca^2+^ channel opening and Ca^2+^ influx by KCl for 24 h was sufficient for increasing the proportion of SA-β-gal-positive cells after KCl wash away (Fig. 6e). The levels of senescent marker proteins p53, p21 and p16 also increased after KCl wash away (Fig. 6f). These results are consistent with our idea that Ca^2+^ influx is required and sufficient for p53 induction during PMD-Sen (Fig. 6g).

### The specific mRNA expression transition in PMD-Sen

To understand the dynamics of gene expression profile changes during PMD-Sen, we performed mRNA-seq using the WI-38 cells collected at each timepoint during senescence progression (day 0 to day 16; day 0 corresponds to the day of the SDS wash away; Extended Data Fig. 8d–g) and compared the result with other senescence subtypes—that is, DNA damage response-dependent senescence (DDR-Sen) by DXR treatment, Ca^2+^-dependent senescence (Ca^2+^-Sen) by KCl treatment and replicative senescence (RS) by repeated passaging. We analyzed the mRNA-seq results using Ingenuity Pathway Analysis (IPA) software, which allows us to use manually curated cell-type-specific databases, enabling reliable prediction of the functions of pathways and genes. First, we identified the pathways significantly activated/inhibited during PMD-Sen, in comparison with DDR-Sen, Ca^2+^-Sen and RS (Supplementary Fig. 4). The enrichment of the top canonical pathways was examined using Fisher’s exact test. This analysis successfully identified 21 top canonical pathways that are activated/inhibited during PMD-Sen whose activation *z*-score values are greater than 2 (activation) or smaller than −2 (inhibition). IPA’s *z*-score indicates a predicted activation or inhibition of a pathway/gene, where a positive *z*-value connotates an overall pathway’s activation and a negative *z*-value connotates an overall pathway’s inhibition.

**Fig. 7:**
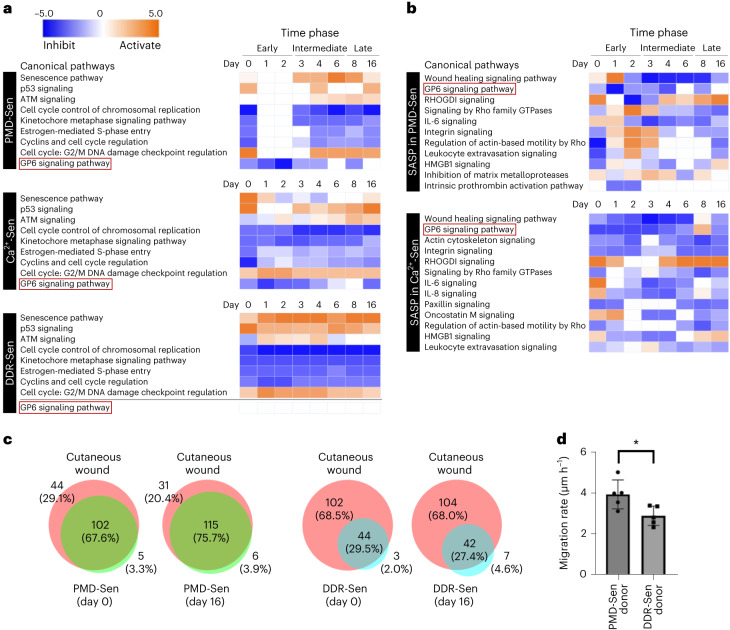
Transcriptional profiling by RNA-seq reveals potential in vivo functions of PMD-dependent senescent cells. **a**, Heat maps generated by IPA comparison analysis show top canonical pathways affected by the differentially expressed mRNAs in PMD-Sen, Ca^2+^-Sen and DDR-Sen. See also Supplementary Fig. 4. PMD-Sen, PMD-dependent senescent cells; Ca^2+^-Sen, Ca^2+^-dependent senescent cells; DDR-Sen, DNA damage response-dependent senescent cells. Orange and blue indicate positive and negative activation *z*-score, respectively; white indicates no activation. Red outlines highlight the GP6 signaling pathway, which is inhibited in PMD-Sen and Ca^2^^+^-Sen but not in DDR-Sen. **b**, Heat maps generated by IPA comparison analysis show top canonical pathways affected by the SASP factors differentially expressed in PMD-Sen and Ca^2+^-Sen. See also Supplementary Fig. 5. **c**, Overlapping canonical pathways in PMD-Sen and cutaneous wounds and in DDR-Sen and cutaneous wounds. RNA-seq results of cutaneous wounds were obtained from the public datasets (GSE141814). See also Extended Data Fig. 9. Day 0 and day 16 are shown. **d**, PMD-Sen cells accelerate wound healing in vitro in a paracrine manner more than DDR-Sen cells do. Young cells (recipient) were co-cultured with PMD-Sen cells (donor) or DDR-Sen cells (donor). Cell-free gaps (500 μm) were made in the layer of young cells (recipient). Cell migration rate was calculated based on the filled cell-free area in 36 h. See also Extended Data Fig. 10. *P < 0.05, exact value: 0.0254, by two-tailed unpaired Student’s *t*-test. Data in **d** are presented as mean (horizontal bars) ± s.d. (whiskers) of five biological replicates. Source data

According to the same cutoff *z*-score values, we also identified 21 top canonical pathways in Ca^2+^-Sen and DDR-Sen. Ten out of 21 pathways were common among PMD-Sen, DDR-Sen and Ca^2+^-Sen (Supplementary Fig. 4, gray boxes, and the bottom-left table). The common features of PMD-Sen, Ca^2+^-Sen and DDR-Sen are the inhibition of cell-cycle-related pathways and the activation of the senescence pathway (Fig. 7a and Supplementary Fig. 4). Although the senescence pathway was activated in all senescence subtypes examined here, we noticed that the kinetics of activation was different. In PMD-Sen, the senescence pathway was activated only on day 3 and later (Fig. 7a). In contrast, the senescence pathway was steadily upregulated in DDR-Sen. These results suggest that the progression of senescence is slower in PMD-Sen than in DDR-Sen.

### Glycoprotein VI signaling pathway inhibition is associated with SASP

Although the inhibition of cell-cycle-related pathways and the activation of the senescence pathway are the common features of all senescent cell subtypes, there are pathways differentially regulated among different senescent cell subtypes. The IPA pathway comparison analyses identified glycoprotein VI (GPVI) signaling pathway that was strongly inhibited in early PMD-Sen and Ca^2+^-Sen but not in DDR-Sen (Fig. 7a and Supplementary Fig. 4). These results raise a possibility that GPVI signaling pathway inhibition is downstream of Ca^2+^ influx because Ca^2+^ influx is one of the earliest common cellular processes after SDS treatment and KCl treatment.

Next, we extracted the SASP factors, based on the SASP Atlas^33^, from the differentially expressed genes identified in our mRNA-seq (Supplementary Table 4). Using these SASP factors, we performed IPA pathway analysis and found that the GPVI signaling pathway was strongly inhibited in early PMD-Sen (Fig. 7b and Supplementary Fig. 5). These results suggest that the downregulation of GPVI signaling pathway is closely associated with the SASP factors.

### Potential link between wound healing and PMD-Sen

**Extended Data Fig. 9:**
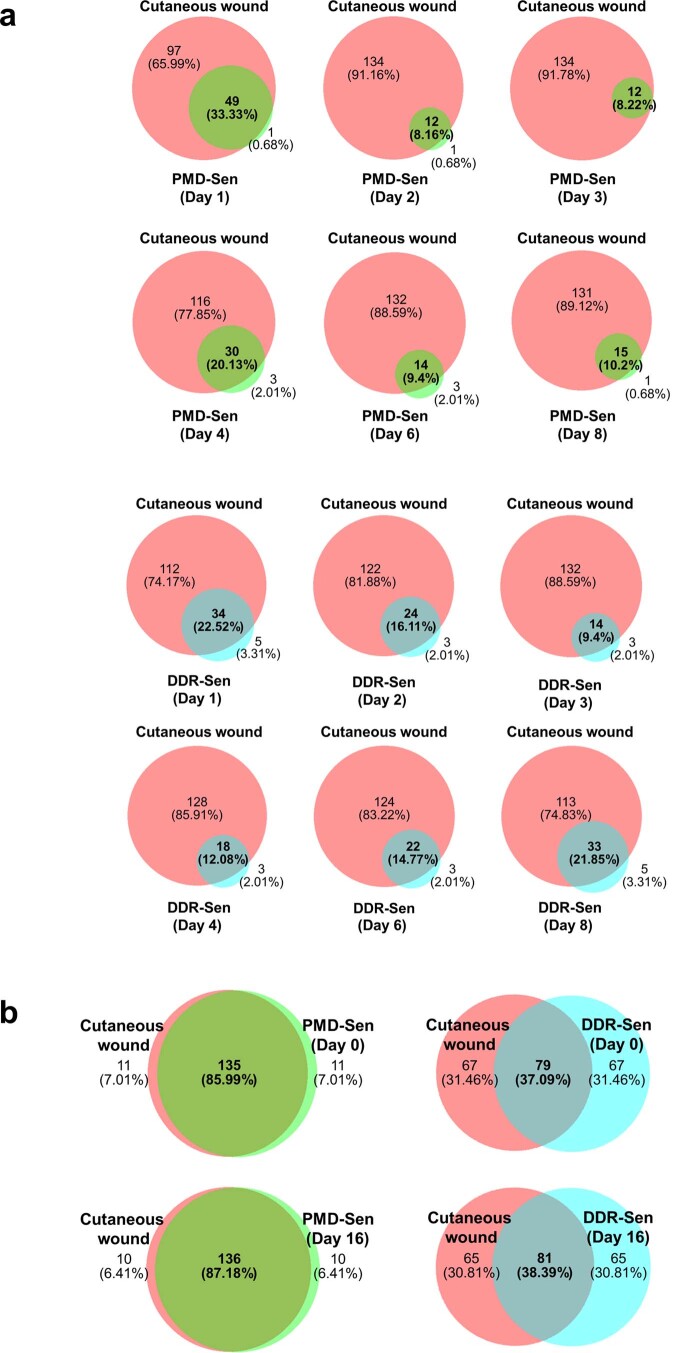
The overlapping pathways between PMD-Sen and cutaneous wounds as well as DDR-Sen and cutaneous wounds. **(a)** Venn diagrams were prepared as in Fig. 7c. Day 1, 2, 3, 4, 6, and 8 are shown. **(b)** Venn diagrams were prepared as in Fig. 7c except for the fact that top 146 pathways were used for each data set regardless of the z-score significance cutoff line.

In the IPA pathway analysis of SASP factors differentially expressed in senescent cells, the transient upregulation of wound healing SASP factors was observed in PMD-Sen (Fig. 7b and Supplementary Fig. 5). Therefore, we speculated that plasma membrane damage could be the in vivo trigger of the senescent cell accumulation around the tissue injury sites. To explore this possibility, we compared a publicly available mRNA-seq dataset derived from cutaneous wound cells^34^ with our mRNA-seq results. Strikingly, we found that the pathways that were significantly changed in cutaneous wound cells substantially overlapped with PMD-Sen cells, particularly on day 0 or day 16 (Fig. 7c, Extended Data Fig. 9a,b and Supplementary Tables 5 and 6). To exclude the possibility that this tendency is an artifact due to fewer number of pathways identified in DDR-Sen, we used the same number of pathways (top 146 pathways) for all datasets, regardless of the *z*-score cutoff line (Extended Data Fig. 9b and Supplementary Table 6). We verified that the result was consistent with the earlier analyses.

**Extended Data Fig. 10:**
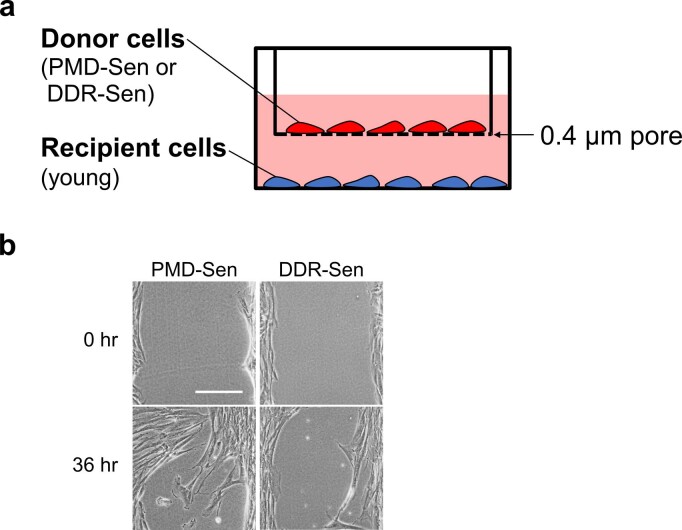
PMD-Sen accelerates wound healing in vitro in a paracrine manner more than DDR-Sen do. **(a)** Non-senescent recipient cells (young) were co-cultured with PMD-Sen cells (donor) or DDR-Sen cells (donor). **(b)** Representative images of recipient cells (young) after 0 and 36 hours after cell-free gap formation are shown. Scale bar, 100 μm. This experiment was independently repeated five times with similar results. Related to Fig. 7d. This experiment was independently repeated five times with similar results.

To investigate whether PMD-Sen cells have functions associated with wound healing in vitro, we examined whether PMD-Sen cells alter the migration rate of young normal fibroblast cells in a paracrine manner. Indeed, the migration assays in a co-culture setup showed that PMD-Sen cells accelerate the migration compared to DDR-Sen cells (Fig. 7d and Extended Data Fig. 10). These results suggest that PMD-Sen cells have the potential to accelerate tissue wound healing in a paracrine manner.

### PMD-Sen and Rep-Sen have PS-externalized spots and blebs

**Fig. 8:**
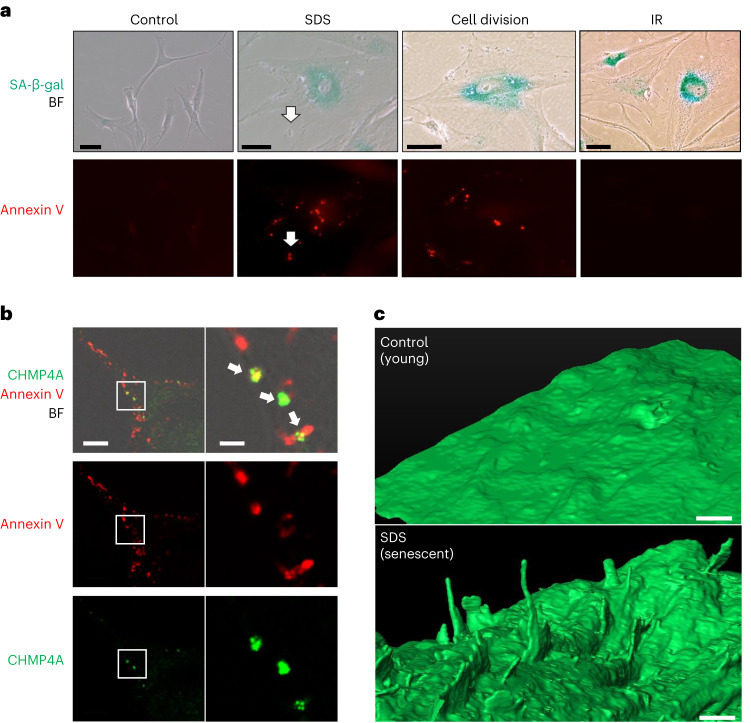
Local PS externalization is associated with PMD-dependent and replicative senescence. **a**, SA-β-gal (green) and Annexin V–Alexa Fluor 488 (red) signals were observed in HCA2 senescent cells induced by three different senescence induction methods (6 d after SDS wash away (SDS) and RS (cell division) and 6 d after IR). Scale bar, 50 μm. White arrows, an Annexin V–positive projection. This experiment was independently repeated three times with similar results. **b**, Rep-Sen WI-38 cells were incubated with Annexin V–Alexa Fluor 647 conjugate, fixed and stained with CHMP4A antibody. The white rectangle region in the left panels is enlarged in the right panels. Green, CHMP4A; red, Annexin V–Alexa Fluor 647 conjugate. BF, bright-field. White arrows, Annexin V and CHMP4A co-localization. Scale bar, left: 10 μm, right: 2 μm. This experiment was independently repeated three times with similar results. **c**, HCA2 cells were treated with 0.01% SDS for 24 h, washed and released into fresh medium. The cells were fixed after 6 d. SDS-treated (SDS, senescent) and untreated (Control, young) cells were analyzed by FIB-SEM. Scale bar, 1 μm. This experiment was independently repeated three times with similar results.

Because PS-externalized spots/blebs were observed after PMD (Fig. 1h,i and Extended Data Fig. 3a–d), we determined whether such spots/blebs exist in senescent cells. Indeed, Annexin V–positive PS-externalized spots/blebs were detected in PMD-Sen cells (Fig. 8a; SDS). Intriguingly, the replicative senescent cells (Rep-Sen cells), but not IR-treated DDR-Sen cells, had the Annexin V spots/blebs (Fig. 8a; cell division and IR). Analogous to the PMD-Sen cells, ESCRT-III (CHMP4A) signals were detected at 21.5 ± 5.0% of PS-externalizing spots/blebs in Rep-Sen cells (Fig. 8b). Some PS-externalizing spots co-localize with projection-like structures in the bright-field image (Fig. 8a, white arrows); therefore, we performed a focused ion beam scanning electron microscope (FIB-SEM) analysis. We found that untreated young normal human fibroblasts (HCA2) had smooth plasma membranes (Fig. 8c, upper). In contrast, PMD-Sen cells had projections (Fig. 8c, lower). The heights of the projections were diverse (280 nm to 2.5 μm). The widths of the projections were relatively uniform (100–130 nm). Together, these results demonstrate that PS-externalizing projections are the common feature of PMD-Sen cells and Rep-Sen cells but not of DDR-Sen cells.

To investigate whether the accumulation of PS-externalizing spots/blebs plays a causative role in cellular senescence induction, we observed the PS-externalizing spots/blebs after ESCRT-III (CHMP4B) overexpression. However, CHMP4B suppressed major cellular senescence features but did not significantly suppress the formation of PS-externalizing spots/blebs (Supplementary Fig. 6). These results suggest that the formation of PS-externalizing spots/blebs is not a cause of cellular senescence but a consequence of PMD.

## Discussion

Here we report that PMD limits replicative lifespan in budding yeast and induces stress-dependent premature senescence in normal human fibroblasts. We developed a simple and universal method to induce PMD and designed a systematic genome-wide screen using budding yeast. Based on the screen, we found that PMD limits replicative lifespan in budding yeast and that overexpression of *VPS4* and *SNF7* extends it. In normal human fibroblasts, PMD induces premature senescence mediated by Ca^2+^ and p53, and overexpression of CHMP4B suppresses it. The transition of mRNA expression profiles is different between PMD-Sen cells and DDR-Sen cells; PMD-Sen cells accelerate wound healing in vitro in a paracrine manner more than DDR-Sen cells. Our work proposes an underappreciated subtype of senescent cells: PMD-Sen cells.

### ESCRT extends replicative lifespan in budding yeast

ESCRT is involved in various cellular processes, including cytokinesis, plasma membrane repair, nuclear membrane repair, multivesicular body formation, vacuolar membrane repair, autophagosome formation and microautophagy of the endoplasmic reticulum^10,35,36^. In the present study, we found ESCRT in our screen for PMD response factors. Previously, ESCRT’s involvement in replicative lifespan was reported by Yang et al.^37^. Yang et al. found that ESCRT mutants *did2*Δ and *vps24*Δ have a long replicative lifespan. Intriguingly, they also reported that another ESCRT mutant, *snf7*Δ, has a short replicative lifespan. These results strongly suggest a close and complex relationship between ESCRT functions and replicative lifespan regulation. Consistent with the findings of Yang et al., we show here that ESCRT is important for budding yeast replicative lifespan regulation based on two lines of evidence: (1) a yeast mutant lacking an ESCRT-III component (*snf7*Δ) showed a short replicative lifespan (Fig. 2e and Extended Data Fig. 5a), and (2) *VPS4* overexpression and *SNF7* overexpression extended the replicative lifespan of budding yeast (Fig. 2f and Extended Data Fig. 5b).

ESCRT can contribute to the replicative lifespan of budding yeast by at least two scenarios that are not mutually exclusive. First, ESCRT-dependent plasma membrane repair may prevent cell lysis and extend the replicative lifespan through the ESCRT’s function in plasma membrane repair as established in human cells^10,35^ and recently suggested in yeast^38^. Second, ESCRT-dependent vacuolar membrane repair may extend the replicative lifespan of budding yeast. This explanation also makes sense because vacuolar acidification defects limit the replicative lifespan in budding yeast^39,40^.

ESCRT is also involved in nuclear envelope repair^10^. However, we found that SDS treatment did not induce marked nuclear envelope deformation (Fig. 1f and Extended Data Fig. 2a), and ESCRT did not accumulate at the nuclear membrane after SDS treatment (Extended Data Fig. 3d, green). Therefore, it is unlikely that ESCRT contributes to the replicative lifespan via the nuclear envelope repair. Our study demonstrates that the PMD response shares many regulators, including ESCRT, with replicative lifespan regulation and that incomplete PMD response explains a mechanism underlying short replicative lifespan in budding yeast.

We demonstrate here that transient overexpression of ESCRT-III (CHMP4B) can suppress PMD-dependent acute cellular senescence in normal human fibroblasts (Fig. 4). However, we did not investigate the contribution of ESCRT to replicative lifespan in normal human fibroblasts. Further studies should be conducted to address whether ESCRT is involved in replicative lifespan regulation in higher eukaryotes.

### Conserved stress-dependent cell cycle arrest mechanisms

Budding yeast has been used as a model of replicative lifespan regulation in eukaryotic cells. In telomere biology, at least three features are common between budding yeast and human cells: (1) the telomerase deficiency induces senescence in budding yeast^41^; (2) telomere shortening causes senescence via permanent activation of DNA damage checkpoints in both budding yeast and human cells^42^; and (3) telomere shortening-dependent DNA damage checkpoints can be bypassed due to adaptation in both human cells and budding yeast^43,44^. Thus, yeast can serve as a model to study basic senescence mechanisms associated with cell cycle regulation. However, there are limitations. For example, yeast does not have the key senescence regulators, such as p53 and SASPs. Thus, yeast can serve as a model to study senescence-triggering cell cycle checkpoints but not all aspects of cellular senescence mechanisms.

### Molecular mechanisms underlying PMD-dependent senescence

We previously reported that PMD induces cyclin-dependent kinase (CDK) inhibitor Sic1 upregulation in budding yeast^14^. Analogous to budding yeast, here we show that PMD promotes upregulation of the two CDK inhibitors, p21 and p16, in normal human fibroblasts (Fig. 3e and Extended Data Fig. 6e,f).

The next question is how PMD triggers the CDK inhibitor upregulation. We demonstrate that p53 was essential for the upregulation of p21 in normal human fibroblasts (Fig. 5d), but the DNA damage response pathway was dispensable for it (Fig. 5b,c). Consistent with these results, we found that p53 is phosphorylated at Ser33 but not at Ser15 (Fig. 3e). p53-Ser33 can be phosphorylated by several kinases, including p38 MAPK, Cdk5/7/9 and GSK3β^45–48^. The kinase responsible for PMD-dependent phosphorylation of p53-Ser33 should be identified in the future.

Another group demonstrated that high cytosolic Ca^2+^ levels induce mitochondrial impairment, leading to upregulation of p53–p21 via cAMP-responsive element-binding protein (CREB) in the mice fibroblast-like cell line L929 (ref. ^49^). Consistently, we show here that (1) cytosolic Ca^2+^ increases after PMD^1–3^ in the human normal fibroblasts WI-38 (Extended Data Fig. 8a–c), (2) BAPTA-AM, cell-permeable Ca^2+^ chelator, attenuates PMD-Sen, and (3) enforced Ca^2+^ influx by KCl was sufficient for inducing various senescence features (Fig. 6a–f). Therefore, Ca^2+^ influx after PMD may mediate the upregulation of the p53–p21 axis in normal human fibroblasts. The absence of p53 in budding yeast suggests that the molecular details are not identical in budding yeast and human cells. Nonetheless, the upregulation of CDK inhibitors is a common mechanism promoting the PMD-dependent cell cycle arrest in both systems.

### Transcriptomic profiling of the senescent cell subtypes

In this study, we revealed the time-resolved transcriptomic profiles of PMD-Sen cells in comparison with other senescent cell subtypes. We found that, although the transient PMD treatment significantly altered the transcriptomic profile (on day 0), most of the upregulated/downregulated pathways returned to the basal status (similar to the before-the-treatment sample) on 1–2 d after the treatment (Fig. 7). This ‘gap’ period delayed the senescence progression after PMD compared to the senescence progression after DNA damage. In the PMD-Sen cells, the senescence pathway was activated only on the third day after the treatment and later, suggesting that the cellular processes on 0–2 d are unique to the PMD-Sen cells.

### Potential in vivo function of the PMD-Sen cells

Senescent cells accumulate around the wound—the tissue injury site—in vivo (mice and humans)^16,50^. This was shown by the increase of p21-positive, p16-positive or SA-β-gal-positive cells in response to wounding^50–53^. Those senescent cells contribute to optimal wound healing via SASP; the SASP mediates senescent cell clearance and tissue regeneration^16,54^. However, the origin of the senescent cells near the wound remained unclear. In the present study, we found that PMD induces the transient upregulation of wound healing SASP factors (Fig. 7b and Supplementary Fig. 5). Furthermore, we found that the pathways that were significantly changed in cutaneous wounded cells in mice skin were overlapped with PMD-Sen cells compared to DDR-Sen cells (Fig. 7c and Extended Data Fig. 9), and co-culturing with PMD-Sen cells accelerated migration speed of non-senescent cells in a paracrine manner (Fig. 7d). Together, these results are consistent with our idea that the PMD-Sen cells may contribute to tissue wound healing in vivo. This possibility needs to be further examined in future studies.

### Local PS externalization in PMD-Sen and Rep-Sen cells

In our study, we observed PS-externalizing spots/blebs in PMD-Sen and Rep-Sen cells, but they were absent from DDR-Sen cells (Fig. 8a). Although PS externalization after PMD was previously reported^17^, the origin of the PS-externalizing spots/blebs in the Rep-Sen cells remains unknown. Plasma membrane repair and cytokinesis share the membrane resealing machinery, including ESCRT^3,10^. Therefore, repeated cytokinesis could induce the accumulation of the PS-externalizing spots/blebs during RS. This idea is consistent with our finding that PS was externalized at the bud scars, former cytokinesis sites, in budding yeast (Fig. 1d). Alternatively, the lipid scramblases^55^ could be activated in Rep-Sen cells. In either case, our results demonstrate that the accumulation of PS-externalizing spots/blebs is a feature of PMD-Sen and Rep-Sen cells.

To address whether the formation of PS-externalizing spots/blebs is a cause of cellular senescence, we observed externalized PS signals after overexpression of the membrane repair factor ESCRT-III (CHMP4B). This experiment was based on our assumption that if PS-externalizing spots/blebs cause cellular senescence, overexpression of membrane repair factor may remove them and bypass cellular senescence. Contrary to our initial assumption, we found that CHMP4B overexpression suppressed several features of cellular senescence, although it did not suppress the formation of PS-externalizing spots/blebs (Supplementary Fig. 6). These results suggest that the formation of PS-externalizing spots/blebs is not the cause of cellular senescence but the phenotype associated with two senescent cell subtypes, PMD-Sen cells and Rep-Sen cells. The remaining question was how CHMP4B was able to bypass cellular senescence without removing the PS-externalizing spots/blebs. One explanation could be that CHMP4B overexpression accelerates membrane resealing without removing PS-externalizing spots/blebs. Rapid membrane resealing may be sufficient to suppress drastic Ca^2+^ influx and subsequent cellular senescence. The mechanism underlying CHMP4B-dependent suppression of senescence induction should be further investigated in the future.

Overall, our results highlight an underappreciated subtype of senescent cells and raise many new questions to be addressed. Recent studies demonstrated that senolytic drugs clearing senescent cells ameliorate age-associated disorders^56,57^. Our study, therefore, may provide one potential explanation for the in vivo origin of senescent cells and serve as a basis for further studies aimed at developing new therapeutic strategies for PMD-associated diseases, including muscular dystrophy and Scott syndrome, as well as organismal aging.

## Methods

### Yeast media, strains and genetic manipulations

Standard procedures were employed for DNA manipulations as well as for *Escherichia coli* and *Saccharomyces cerevisiae* handling. YPD was used in most experiments. SD was used for live cell imaging. Yeast culture was performed at 25 °C unless otherwise indicated. The *S. cerevisiae* strains used in this study are listed in Supplementary Table 7.

### Penetration assay

For the penetration assay in yeast cells, an overnight culture in YPD was refreshed and incubated for an additional 2–6 h until the optical density at 600 nm (OD_600_) reached 0.1–0.3. For Fig. 1a,b, the media were switched to YPD+20 mM EGTA, YPD+0.02% SDS, YPD+20 mM EGTA+0.02% SDS and control YPD for 15 min. All these media contained 2 ng ml^−1^ DAPI. DAPI-positive cells were counted under Celldiscoverer 7 (Zeiss, ZEN 2.6). For the penetration assay in human cells, DMEM containing DAPI, PI or FM1-43 was incubated with or without PMD stimuli.

### Fluorescence imaging and image analysis in yeast

The laser damage assay was performed as described previously^14^ with modifications. In brief, laser damage was induced using a laser scanning confocal microscope (LSM 780 NLO (Zeiss, ZEN 2.3)) equipped with a ×63 objective; a 405-nm, 488-nm and 561-nm laser; and a standard PMT and GaAsP detector. The region of interest (ROI) for laser IR with a 405-nm laser was set as approximately 0.5 µm in diameter. The laser power of the 405-nm laser was approximately 50% to induce local plasma membrane damage without cell lysis. A yeast culture grown overnight was refreshed and incubated for an additional 2–6 h until the OD_600_ reached 0.1–0.3. Cells were then spotted onto an agarose bed (SD medium+1.2% agarose) on glass slides. Signal quantification was performed using Fiji/ImageJ software^58^.

### Electron microscopy of yeast

Yeast cells sandwiched between copper grids were subjected to quick freezing using an isopentane/propane mixture of cold chemicals. The copper grid was diverged under liquid nitrogen gas atmosphere, soaked in 2% osmium/acetone solution and then stored at −80 °C for 2 d. The samples were placed at −20 °C for 3 h, at 4 °C for 1 h and then at room temperature. The samples were washed with acetone and subsequently with propylene oxide and then embedded in epoxy resin (Quetol 651). Observation was performed under a JEM-1011J (JEOL) or a JEM-1400Plus (JEOL) electron microscope.

### Yeast genome-wide screen

Non-essential gene deletion library^18^ and DAmP library in which mRNA levels of essential genes are decreased to 20–50% (ref. ^19^) were used in the yeast genome-wide screen. SDS sensitivity was determined using freshly prepared YPD+0.02% SDS plates. Cells grown on YPD plates for 3 d were pin-plated onto the SDS-containing plates and incubated at 30 °C for 3 d. The mutants identified in the first screening (249 mutants) were then freshly grown from −80 °C stock. Single colonies were isolated, streaked on YPD and YPD+0.02% SDS plates and incubated at 30 °C for 3–5 d. The incubation time was optimized for each strain. We obtained 109 mutants from the library, which were lethal on YPD+0.02% SDS plates. Subsequently, we independently constructed 119 mutants using our wild-type (BY23849) as a parental strain. At least three independent colonies were constructed per each mutant. Single colonies were isolated, streaked on YPD and YPD+0.02% SDS plates and incubated at 25 °C for 3–5 d (optimized for each strain). In the end, we obtained 48 mutants as confirmed hits, which were reproducibly lethal specifically on YPD+0.02% SDS plates.

### Replicative lifespan analysis in yeast

Replicative lifespan analysis was performed as previously described^21^. In brief, newly formed daughter cells were separated after every cell division by a glass needle for tetrad analysis under the microscope. During the night, the plates were incubated at 12 °C. For the mechanical stress assay using a glass needle, a 12-mm-diameter cover glass was attached to the YPD plate, and mechanical stress was induced by hitting the cell on the glass needle against the cover glass (Extended Data Fig. 5d). The cells were then returned to the YPD plate, and the number of newly budded daughter cells was counted.

### Co-staining of Annexin V–Alexa Fluor 568 and Calcofluor white in yeast

Externalized PS detection in yeast was performed as previously described^59^ with some modifications. Yeast cells were cultured in YPD for overnight, diluted to 1/10 in fresh YPD and then incubated for an additional 2–3 h. Cells were collected and washed once with Sorbitol buffer (35 mM potassium phosphate, pH 6.8, 0.5 mM MgCl_2_, 1.2 M sorbitol). Cells were incubated in 98 μl of Sorbitol buffer + 2 μl of 2.5 mg ml^−1^ Zymolyase 100T (Seikagaku) for 90 min (wild-type) or 60 min (*pTEF1-VPS4*) at room temperature with gentle shaking. Cells were then washed once with Sorbitol buffer, resuspended in 19 μl of Annexin V binding buffer + 1 μl of Annexin V–Alexa Fluor 568 and incubated for 20 min at room temperature with gentle shaking. Supernatant was removed, and cells were suspended in 9.8 μl of Annexin V binding buffer + 0.2 μl of Calcofluor white stain (Sigma-Aldrich, Fluka). Images were acquired using an AXIO Observer.Z1 (Zeiss, ZEN 2.3).

### Cell culture methods for human cells

Cell culture methods, senescence induction by IR and SA-β-gal staining were performed as previously described^22,60^ with some modifications. In brief, cells were fixed for 3 min in 2% paraformaldehyde (PFA) and incubated for approximately 16 h at 37 °C with a staining solution containing 1 mg ml^−1^ X-gal, 40 mM citric acid in sodium phosphate buffer (pH 6.0), 5 mM K_4_[Fe(CN)_6_]3H_2_O, 5 mM K_3_[Fe(CN)_6_], 150 mM NaCl and 2 mM MgCl_2_. Immunoblotting was performed following a standard protocol. Antibodies used in this study are listed in Supplementary Table 8, and antibody validation was performed by the distributors. Their webpage links are provided in the Reporting Summary. Images of blotted membranes were obtained by ChemiDoc Touch MP (Bio-Rad). HeLa cells and normal human fibroblasts (HCA2, WI-38 or BJ) were used (HeLa cell line, endocervical adenocarcinoma, female, from RIKEN BRC, cat. no. RCB0007, RRID: CVCL_0030; WI-38 cells, fibroblast, female, PDL36.6 from RIKEN BRC, cat. no. RCB0702, RRID: CVCL_0579; WI-38 cells, fibroblast, female, PDL32.2 from JCRB, cat. no. IFO50075, RRID: CVCL_0579; and BJ cells, fibroblast, male, PDL24.0 from the American Type Culture Collection, cat. no. CRL-2522, RRID: CVCL_3653). HCA2 cells are normal human neonatal foreskin cells kindly gifted by J. Campisi (Buck Institute for Research on Aging) to M.N.^60^ (male, PDL16.0). ATM inhibitor (KU-55933, Sigma-Aldrich), DNA-PK inhibitor (NU7026, Sigma-Aldrich) and cytosolic Ca^2+^ chelator (BAPTA-AM, Alomone Labs) were added to the medium at a final concentration of 10 μM, 10 μM and 1 μM, respectively. For Fig. 3a and Extended Data Fig. 6a,b, cells were grown in the medium with or without SDS (0.005–0.012%), and passage was performed every 3–4 d. After every passage, the cells were grown in the medium without SDS for 16 h and then switched to the SDS-containing medium. For SDS-dependent senescence induction, cells were treated with the medium containing SDS (0.007–0.01%: optimized for cell strains/experimental conditions; Supplementary Fig. 3) for 24 h, washed twice with medium and covered with the fresh medium and then incubated for additional days as indicated in the figure. The SDS concentration was optimized for each experiment, depending on cell type and FBS lot. For reference, see Supplementary Fig. 8. SDS treatment shorter than 24 h did not efficiently induce cellular senescence, even when the concentration of SDS was increased. During the incubation, the medium was changed every 2 d. In Supplementary Fig. 3, the concentrations of SDS for inducing cellular senescence were determined by identifying the lethal concentration for each cell type, and then non-lethal concentrations, which do not induce cell death, were used. Each cell type was cultured in 10% FBS containing medium (DMEM, high glucose) with 0–0.015% SDS for 5 d, and the concentration of non-lethal, sublethal and lethal was determined. For SLO and silica treatment (Fig. 3f–i), WI-38 cells were treated with 200 ng ml^−1^ SLO and 125 μg ml^−1^ silica for 24 h, washed and released into a fresh medium. For laser damage-dependent senescence induction (Extended Data Fig. 6h–j), laser damage assay was performed as described above with modification. Laser damage was induced using a laser scanning confocal microscope, A1R (Nikon NIS-Elements 6.0), equipped with a ×60 objective; a 405-nm, 488-nm and 561-nm laser; and a standard PMT and GaAsP detector. The laser power of the 405-nm laser was 100%. Laser irradiated only at the periphery of the WI-38 cell without inducing detectable DNA damage. For KCl treatment (Fig. 6e,f), WI-38 cells were treated with 75 mM KCl for 24 h, washed and released into a fresh medium. For DXR treatment, the cells were incubated with 250 nM DXR (Cayman Chemical) for 24 h, washed and released into a fresh medium. For replicative senescence, the cells were split every 3–4 d until the cells stop proliferation. For knockdown experiments, WI-38 cells were transfected with siRNAs by Lipofectamine RNAiMAX Transfection Reagent (Thermo Fisher Scientific) or transduced with shRNA mediated by lentivirus. Target sequences are as follows. p53 siRNA (hs.Ri.TP53.13.3, Integrated DNA Technologies): GAGGUUGGCUCUGACUGUACCACCA; p53 shRNA: ACTCCAGTGGTAATCTACT. Annexin V–Alexa Fluor 488 (Thermo Fisher Scientific) staining was performed following the manufacturer’s instructions. Images were acquired using a BZ-9000 all-in-one fluorescence microscope (Keyence) or an LSM 780 confocal microscope (Zeiss). For time-lapse imaging (Extended Data Fig. 3c), WI-38 cells were incubated with the medium containing 0.008% SDS and 1:5,000 diluted pSIVA-IANBD (Novus Biological). Images were acquired by Celldiscoverer 7 (Zeiss, ZEN 2.6).

### CHMP4B expression in WI-38 cells

A control plasmid (GFP) or GFP-CHMP4B was overexpressed in WI-38 cells by 4D-Nucleofector (Lonza, SE cell line solution, program EO-114) according to the manufacturer’s instructions. One million cells were nucleofected with 0.5 μg of DNA of either GFP or GFP-CHMP4B.

### Co-staining of Annexin V and CHMP4A in human cells

Externalized PS was stained with Annexin V–Alexa Fluor 647 conjugate. After the PFA fixation, cells were treated with 0.1% saponin and 5% BSA for 40 min at room temperature. Subsequently, cells were incubated with primary and secondary antibodies. Images were taken with an SP8 confocal microscope (Leica LAS-X).

### Flow cytometer analysis

WI-38 cells were incubated in 100 ng ml^−1^ DAPI-containing DMEM with or without SDS. After washing away, cells were fixed with 4% PFA and resuspended in flow cytometry buffer (1× PBS with 2% FBS). Flow cytometric analysis was performed by Amnis ImageStream × Mk II (Millipore), and data were processed with FCS Express (De Novo Software). Cell aggregates and debris were removed by standard gating strategy.

### Sample preparation for FIB-SEM

Normal human fibroblast cells (HCA2) were fixed with a 3% glutaraldehyde solution in 100 mM cacodylate buffer (pH 7.4) for 1 h at 4 °C. The cells were then washed with 7.5% sucrose, and then en bloc staining was performed following a procedure from National Center for Microscopy and Imaging Research (NCMIR) methods for three-dimensional (3D) electron microscopy (EM) (https://ncmir.ucsd.edu/sbem-protocol). Epon 812-embedded specimens were glued onto an aluminum FIB-SEM rivet with conductive epoxy resin. Next, 15 × 15-μm (1,000 × 1,000 pixels) images were acquired by an MI4000L (Hitachi High-Tech) at 20-nm intervals. More than 200 images were collected to reconstitute a single cell.

### Stack alignment, segmentation and 3D representation

The image stacks were automatically aligned by Fiji/ImageJ software. Plasma membrane structure segmentation and 3D image reconstitution were performed manually with AMIRA version 5.6 software (FEI Visualization Science Group).

### Ca^2+^ imaging

WI-38 cells were plated on glass-bottom dishes. Cells were loaded with a cytosolic Ca^2+^ indicator (5 μM Cal Red R525/650, AAT Bioquest) and imaged using an SP8 confocal microscope (Leica LAS-X). Cytosolic Ca^2+^ dynamics were tracked with three stack images around the focal plane. Fluorescence intensity (Ex 492 nm/Em 500–550 nm and Ex 492 nm/Em 625–675 nm) was recorded every 15 s. For quantification, z-stack images were prepared by Z projection tools (sum slices), and the whole area of each cell was selected by ROI selection tools (Fiji/ImageJ). The average fluorescence ratio (500–550 nm / 625–675 nm) was measured and calculated as *F* value. Δ*F* values were calculated from (*F* − *F*_0_) where *F*_0_ values were defined by averaging four frames before stimulation.

### RNA extraction and qRT–PCR analyses

Cells were lysed in TRIzol. RNA was extracted and purified using an RNA Clean and Concentrator-5 Kit (Zymo Research) following the manufacturer’s instructions. Complementary DNA (cDNA) was synthesized using SuperScript IV VILO Master Mix (Thermo Fisher Scientific). The expression of target genes was determined using QuantStudio 1 Real-Time PCR system (Thermo Fisher Scientific). PCR was performed using PowerTrack SYBR Green Master Mix (Thermo Fisher Scientific) with primer pairs as listed in Supplementary Table 9. The data were statistically analyzed using SPSS version 21.0 software (IBM). One-way ANOVA and Tukey–Kramer test were used to check the significant changes of gene expression. NormFinder software was used to determine the stability values of the reference gene. Changes in gene expression, expressed as fold change, were calculated using the ΔΔCt method, where *ACTB* was used as a reference gene for normalizing the expression. The nested scatter plot was generated to show the fold changes of each replicate (GraphPad Prism).

### EdU staining

For RS, WI-38 cells were passaged until they lost the ability to proliferate and became fully senescent around population doubling level (PDL) 52. For the PMD-Sen and DDR-Sen, young WI-38 cells were treated with SDS or DXR containing medium for 24 h. A Click-iT Plus EdU Cell Proliferation Kit for Imaging, Alexa Fluor 647 dye (Thermo Fisher Scientific) was used for the detection of DNA synthesis. Cells were labeled with 10 μM EdU for 24 h and then treated according to the manufacturer’s instructions. Images were obtained with AXIO Observer 7 (Zeiss, ZEN 3.1).

### mRNA-seq

All samples were sequenced by a NovaSeq 6000 (Illumina). Paired-end cDNA libraries were sequenced with a 2 × 150-bp configuration. Each sample was sequenced at a depth of more than 20 million paired-end reads. RNA sequencing (RNA-seq) datasets have been deposited in the National Center for Biotechnology Information (NCBI) Gene Expression Omnibus (GEO) database with accession number GSE222400.

### Pathway and biological functions analyses using IPA software

IPA software was used to predict networks that are affected by the differentially expressed genes. Details of the identified genes, their quantitative expression values and *P* values were imported into IPA software. The ‘Core Analysis’ function included in IPA software was used to interpret the data in the context of biological processes, pathways and networks^61^. Both upregulated and downregulated genes were defined as value parameters for the analysis. Each gene identifier was mapped to its corresponding protein object and was overlaid onto a global molecular network developed from information contained in the Ingenuity Knowledge Base. A network of proteins was then algorithmically generated based on their connectivity. Right-tailed Fisher’s exact test was used to calculate a *P* value to indicate the probability of each biological function being assigned to the network by chance. IPA comparison analysis was performed in accordance with our established comparison analyses^62^. A heat map was generated based on the activation *z*-scores. The public datasets for the cutaneous wound (Fig. 7c) were adopted from regenerative samples (GSM4213633) in GSE141814 (ref. ^34^). The overlap of the pathways was visualized by the open access application BioVenn^63^.

### Co-culture and migration assays

To create cell-free gaps, ibidi two-chamber inserts were placed in six-well plates, and WI-38 cells were seeded into these chambers. For co-culturing, PMD-Sen and DDR-Sen cells were seeded onto transwell inserts (Extended Data Fig. 10a). The membrane of the transwell inserts had 0.4-μm pores, facilitating the transfer of soluble proteins and small extracellular vesicles. Images were captured at 0 h and 36 h after cell-free gap establishment. The migration rate (μm^2^ per hour) was determined as previously described^64^. In brief, the gap area of each timepoint was calculated as a function of time to derive the cell migration rate.

### Statistics and reproducibility

Microsoft Excel 365 version 1711 or later, GraphPad Prism 9, IPA Fall 2021 or later and SPSS version 21.0 software were used for data presentation and statistical analysis. No explicit sample size calculation was performed. We followed community norms, and sample sizes are similar to those reported in previous publications^15,22^. Exact sample sizes / number of independent experiments are given in the corresponding figure legends, extended data figure legends, supplementary figure legends and supplementary data. For biochemical and spectrometric experiments, each experiment was independently repeated multiple times. In principle, at least three independent experiments were performed for each assay to verify the reproducibility of the results. mRNA-seq was performed with two independent samples. No data were excluded from analysis. All cell culture experiments were randomly assigned to experimental conditions. For microscopic images, the image fields of immunofluorescence and TEM observations and SA-β-gal staining were randomly acquired. Data distribution was assumed to be normal, but this was not formally tested; therefore, data distributions are visualized in each figure. Data collection and analysis were not performed blinded to the conditions of the experiments. The statistical test used to determine the significance of differences is indicated in the figure legends.

### Reporting summary

Further information on research design is available in the Nature Portfolio Reporting Summary linked to this article.

### Supplementary information

Supplementary Information Supplementary Text, Figs. 1–6 and Tables 1–3.

Reporting Summary

Supplementary Table 4 SASP factors identified in the significant genes expressed in PMD-Sen, Ca^2+^-Sen and DDR-Sen.

Supplementary Table 5 Overlapped pathways between cutaneous wound and PMD-Sen/DDR-Sen in the significantly differentially regulated pathways.

Supplementary Table 6 Overlapped pathways between cutaneous wound and PMD-Sen/DDR-Sen in the top 146 pathways regardless of its *z*-score.

Supplementary Table 7 Yeast strains used in this study.

Supplementary Table 8 Antibodies used in this study.

Supplementary Table 9 Primer sequences used for qPCR analyses.

Supplementary Data 1 Statistical source data for supplementary figures.

### Source data

Source Data Figs. 3–6 and Extended Data Figs. 6–8 Unprocessed blots for Figs. 3–6 and Extended Data Figs. 6–8.

Source Data Figs. 1–7 and Extended Data Figs. 2–8 Statistical source data.

## Data Availability

The RNA-seq dataset generated and analyzed during the current study is available in the NCBI GEO repository (www.ncbi.nlm.nih.gov/geo/query/acc.cgi?acc=GSE222400). Source data are provided with this paper. The rest of the data generated or analyzed during this study are included in the published article and its supplementary information files. Externally generated data can be obtained from the following resources: www.ncbi.nlm.nih.gov/geo/query/acc.cgi?acc=GSE141814 and www.saspatlas.com.
