# Supplementary material for: Plasma membrane damage limits replicative lifespan in yeast and induces premature senescence in human fibroblasts
Source: Nat Aging. 2024 Feb 22;4(3):319–35. doi: 10.1038/s43587-024-00575-6 (PMC10950784; doi:10.1038/s43587-024-00575-6)
Supplement: Supplementary file 1 — Supplementary Text, Figs. 1–6 and Tables 1–3. [file 43587_2024_575_MOESM1_ESM.pdf]

nature aging

Article

https://doi.org/10.1038/s43587-024-00575-6

Plasma membrane damage limits replicative lifespan in yeast and induces premature senescence in human fibroblasts

In the format provided by the authors and unedited

Supplementary Text

To characterize the screening hits, the budding yeast gene deletion mutants that failed to grow on SDS-containing YPD plates, we examined cell viability after SDS treatment. We found that ESCRT mutants (*did4*Δ, *snf7*Δ, *stp22*Δ, *vps20*Δ, *vps25*Δ, *vps36*Δ, and *vps24*Δ) survived in the course of the two-hour SDS treatment, whereas V-ATPase mutants (*vma21*Δ, *vph2*Δ, *vma5*Δ, *vma1*Δ, and *vma13*Δ) lost their viability within 30 min (Extended Data Fig. 4a-d).

V-ATPase produces a proton gradient across the vacuolar membrane, enabling Ca^{2+} uptake into the vacuole; the mutants lucking functional V-ATPase show high cytoplasmic Ca^{2+} levels[65]. Since Ca^{2+} influx at the damage site is essential for membrane resealing in higher eukaryotes[6,7], SDS sensitivity in V-ATPase mutants may be explained by the high cytosolic Ca^{2+} concentration in V-ATPase mutants, preventing membrane resealing. Indeed, in our DAPI penetration assay (30 min incubation in YPD+SDS and quick wash with YPD, followed by 5 min incubation with DAPI), V-ATPase mutants (*vma1*Δ and *vma13*Δ) showed high DAPI-positivity (*vma1*Δ: 72.2±14.6, *vma13*Δ: 63.0±8.6; Extended Data Fig. 4e) analogous to SDS and EGTA-treated wild type (Fig. 1a and b). These results raise a possibility that Ca^{2+} influx-dependent membrane resealing is impaired in V-ATPase mutants.

To test the possibility that Ca^{2+} homeostasis is defective in V-ATPase mutants, we monitored subcellular localization of Crz1-GFP, a Ca^{2+}-responsive transcription factor that enters nucleus after various stimuli[66]. After the laser-induced cell wall and plasma membrane damage, Crz1-GFP entered the nucleus within 30 sec (Extended Data Fig. 4f and g). In a V-ATPase mutant *vma1*Δ, even before the laser damage Crz1-GFP signals at the nucleus was comparable to the peak levels of control cells and did not increase after the laser damage. These results support our interpretation that Ca^{2+}-influx detection is

impaired in *vma1*Δ.

Since *cho1*Δ, defective in phospholipid phosphatidylserine (PS) synthesis, was a screening hit, we examined whether PS is involved in plasma membrane/cell wall repair processes after the laser damage. Wild type yeast cells harboring a plasmid of Lact-C2-GFP, a PS marker, were subjected to the laser damage assay. We found that Lact-C2-GFP signal gradually accumulated at the damage site and peaked after ~13 min (Extended Data Fig. 4h and i), consistent with the idea that PS is involved in the plasma membrane/cell wall repair processes.

We next focused on *pep3*Δ and *vps34*Δ. These mutants shared three common phenotypes: 1) the cell viability did not decrease after 2hr SDS treatment (Extended Data Fig. 4d), 2) the cells did not show DAPI penetration after 30 min SDS treatment (Extended Data Fig. 4e), and 3) in the laser damage experiment, a major repair protein Pkc1-GFP failed to be retained at the laser damage site (Extended Data Fig. 4j and k). These results suggest that Pep3 and Vps34 are required for the retention of Pkc1 but not for plasma membrane resealing immediately after the damage or initial Pkc1 recruitment to the damage site.

In summary, here we revealed four cellular processes during plasma membrane/cell wall damage response in budding yeast: 1) V-ATPase-dependent prevention of immediate cell death, 2) Crz1 nuclear import, 3) PS recruitment to the damage site, and 4) Pep3 and Vps34-dependent retention of Pkc1 (Supplementary Fig. 2).

Supplementary Reference

65 Ohya, Y. *et al.* Calcium-sensitive cls mutants of Saccharomyces cerevisiae showing a Pet-phenotype are ascribable to defects of vacuolar membrane H (+)-ATPase activity. *Journal of Biological Chemistry* **266**, 13971-13977 (1991).

66 Stathopoulos-Gerontides, A., Guo, J. J. & Cyert, M. S. Yeast calcineurin regulates nuclear localization of the Crz1p transcription factor through dephosphorylation. *Genes & development* **13**, 798-803 (1999).

Supplemental Figures

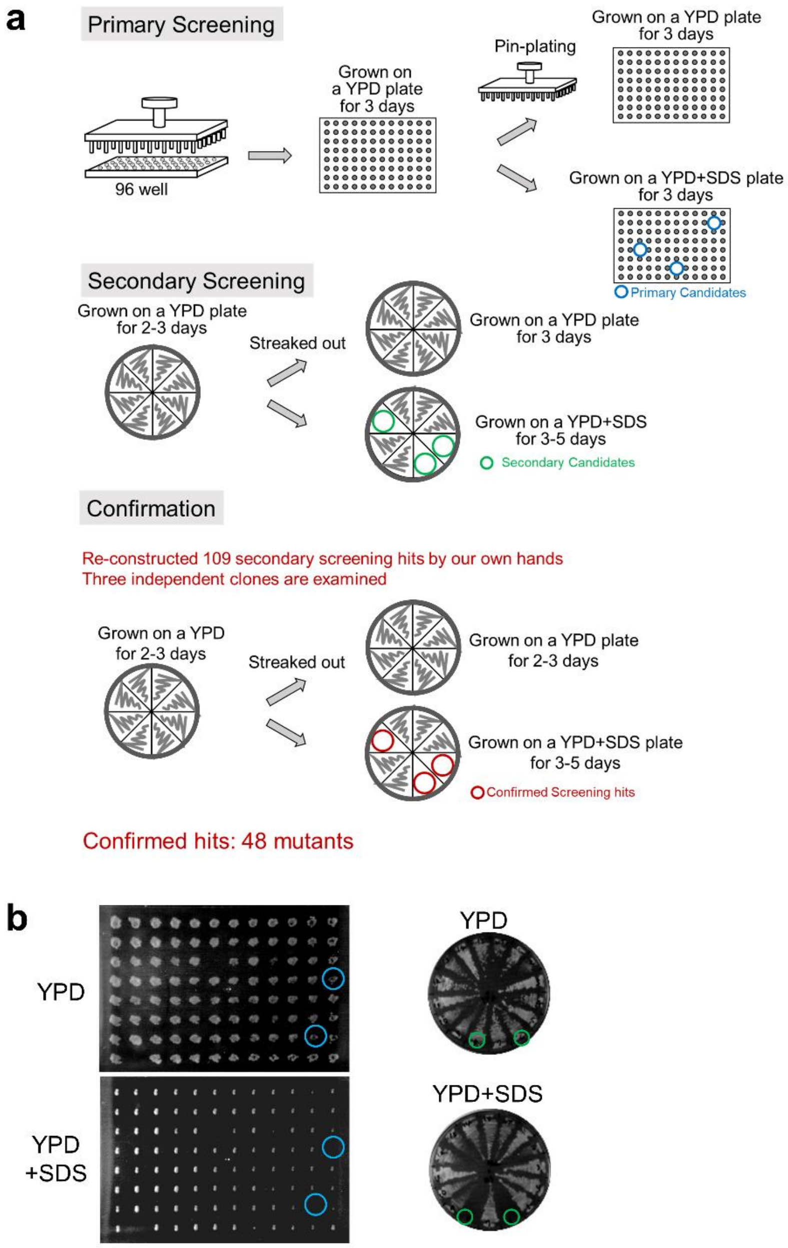

Supplementary Fig. 1. Summary of the screening method

(a) Schematic drawing of the screening method. Blue circle, primary hits (249 mutants); green circle, secondary hits (109 mutants); red circle, confirmed hits (48 mutants). **(b)** Example images of the plates used in the screening.

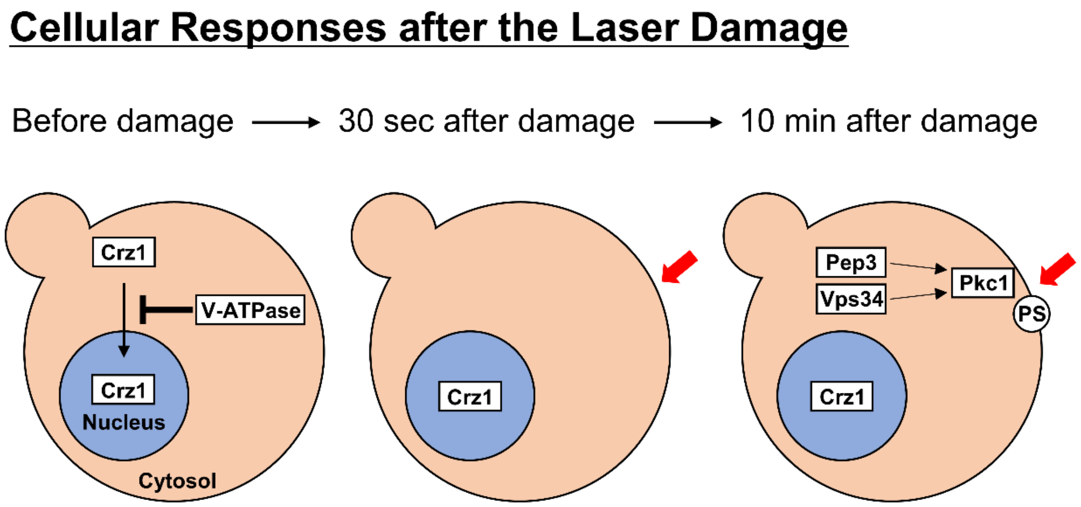

Supplementary Fig. 2. Summary of laser damage responses in budding yeast

Cellular responses after laser damage. Red arrows indicate the laser damage site.

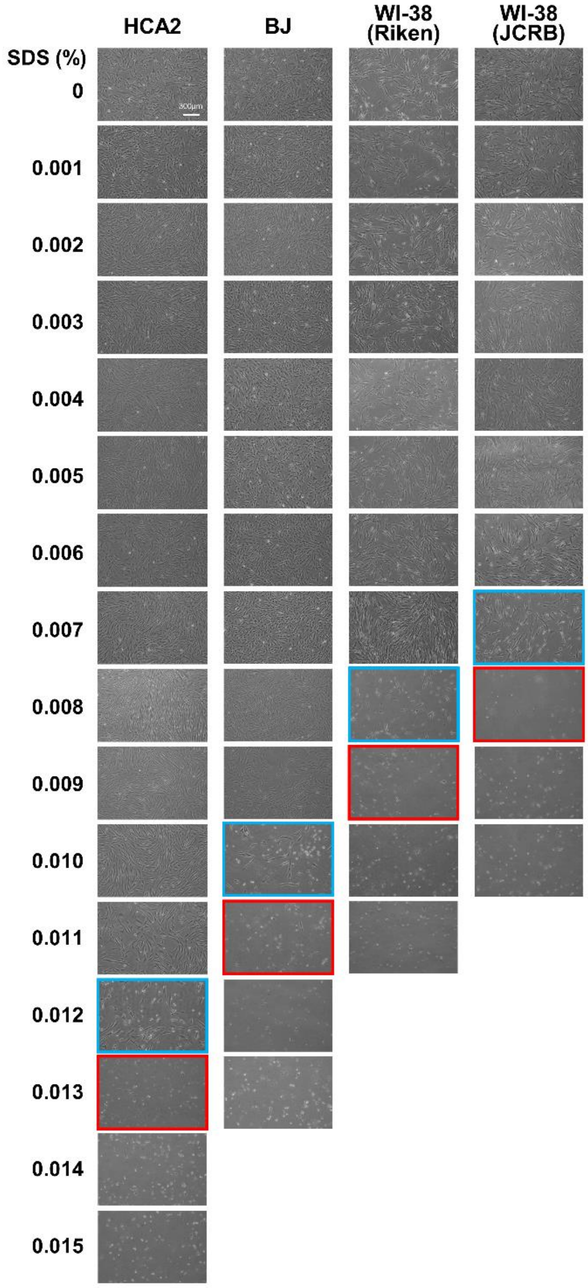

Supplementary Fig. 3. Identification of the optimal SDS concentration for different types of human normal fibroblasts

The four different types of human normal fibroblasts (HCA2, BJ, WI-38 distributed from RIKEN, and WI-38 from JCRB: Japanese Collection of Research Bioresources Cell Bank) were cultured with the different SDS concentrations. The blue boxes indicate sub-lethal SDS concentrations at day 5, and the red boxes are lethal SDS concentrations, which depend on the cell type and distributes. Based on these results, we used the upper limit of

concentration that does not induce cell death after 24 hours of SDS treatment. This experiment was independently repeated three times with similar results.

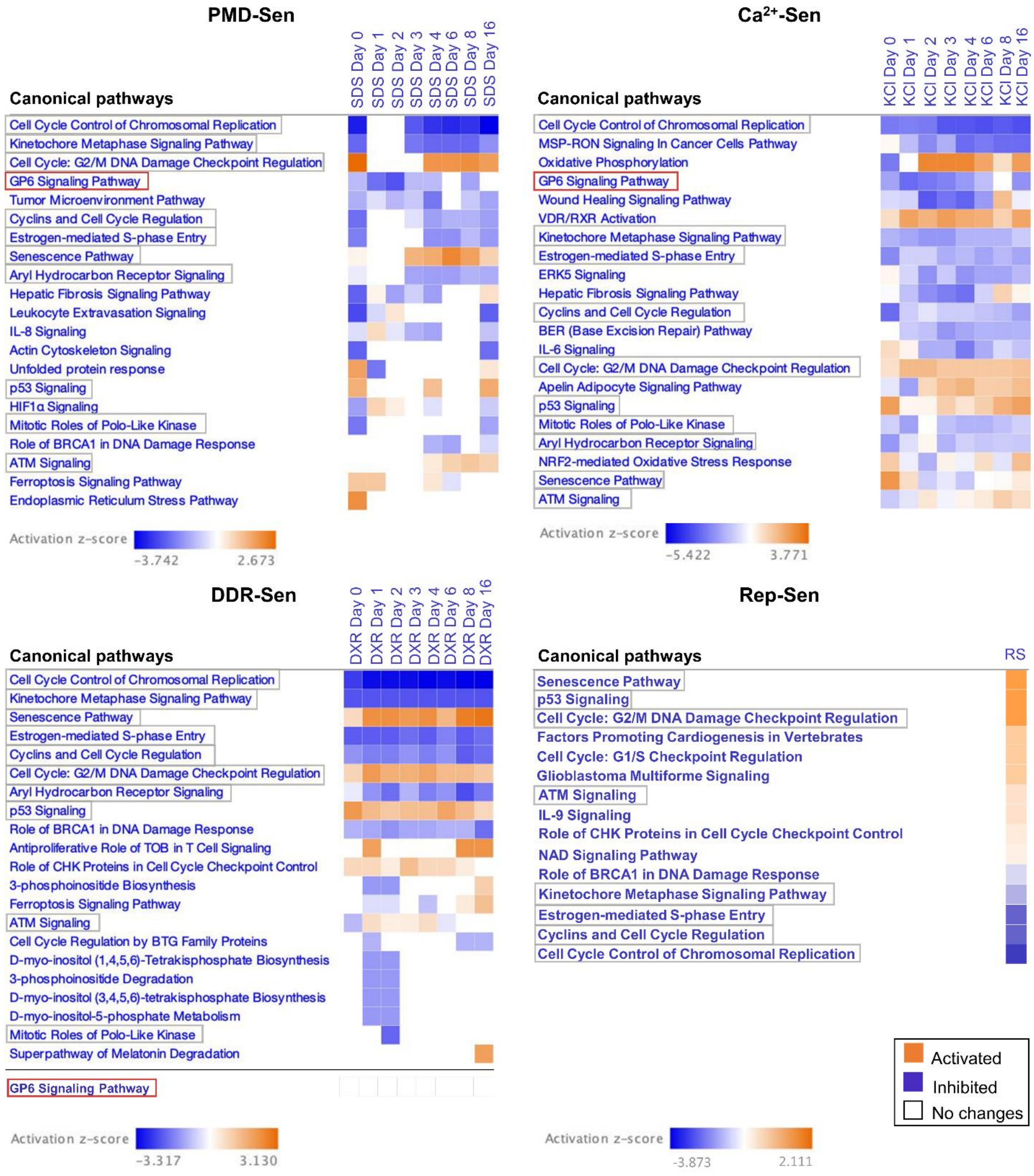

Common pathways in RS, PMD-Sen, Ca^{2+}Sen and DDR-Sen
1) Cell cycle control of chromosomal replication
2) Kinetochore metaphase signaling pathway
3) Cell cycle: G2/M DNA damage checkpoint regulation
4) Estrogen-mediated S-phase entry
5) Cyclins and cell cycle regulation
6) Senescence pathway
7) p53 signaling
8) ATM signaling

Common pathway in PMD-Sen and Ca^{2+}-Sen
1) GP6 signaling pathway

Supplementary Fig. 4. Top canonical pathways in PMD-Sen, Ca^{2+}-Sen, DDR-Sen, and Rep-Sen

Heat maps generated by IPA comparison analysis show top canonical pathways affected by the differentially expressed mRNAs in PMD-Sen, Ca^{2+}-Sen, DDR-Sen, and Rep-Sen. Orange and blue indicate positive and negative activation z-scores, respectively, while white indicates no significant activation z-scores. Related to Figure 7a.

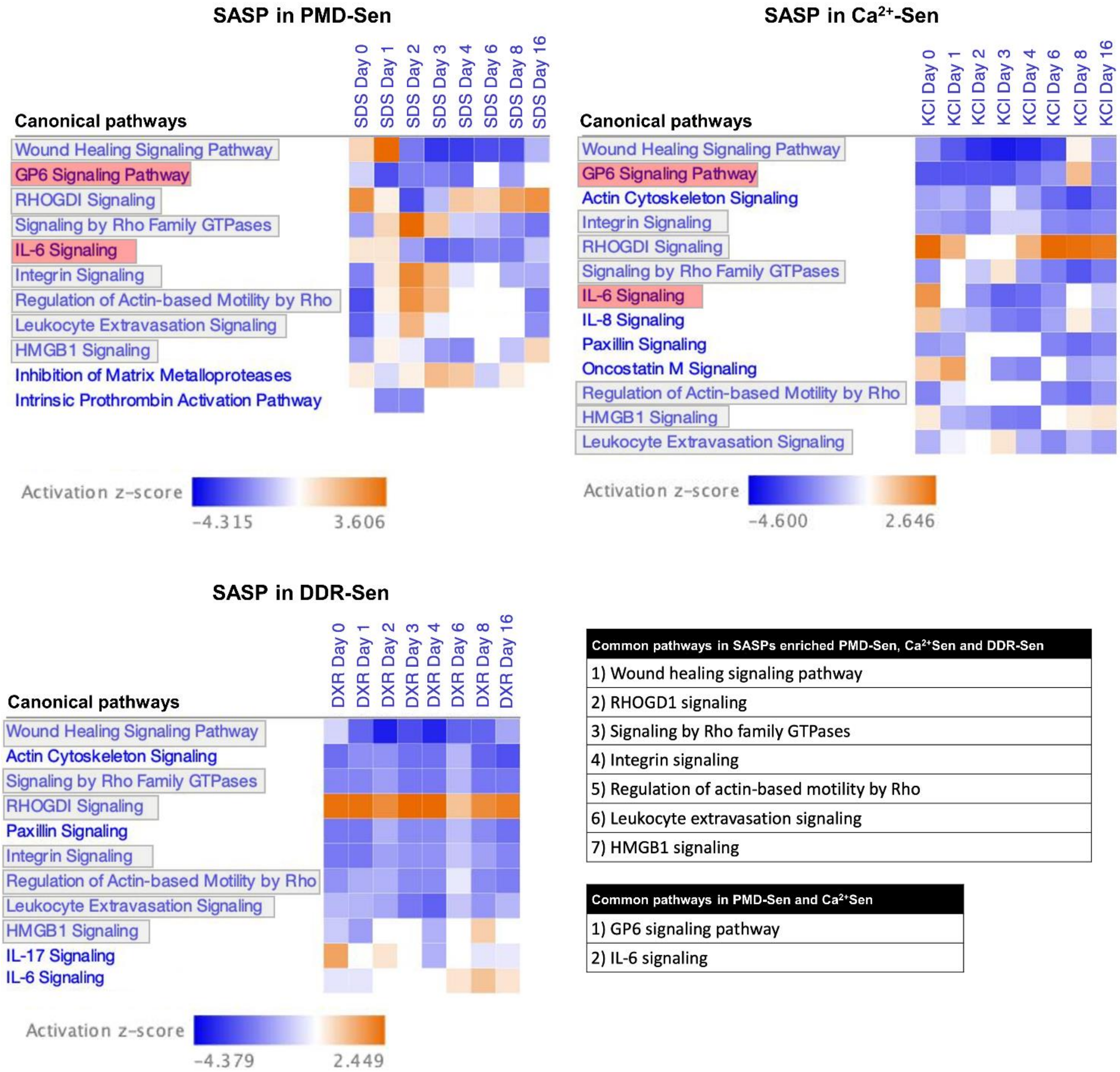

Common pathways in SASPs enriched PMD-Sen, Ca^{2+}Sen and DDR-Sen
1) Wound healing signaling pathway
2) RHOGD1 signaling
3) Signaling by Rho family GTPases
4) Integrin signaling
5) Regulation of actin-based motility by Rho
6) Leukocyte extravasation signaling
7) HMGB1 signaling

Common pathways in PMD-Sen and Ca^{2+}Sen
1) GP6 signaling pathway
2) IL-6 signaling

Supplementary Fig. 5. Top canonical pathways associated with SASP factors in PMD-Sen, Ca^{2+}-Sen, DDR-Sen

Heat maps generated by IPA comparison analysis show top canonical pathways affected by the SASP factors differentially expressed in PMD-Sen, Ca^{2+}-Sen, and DDR-Sen. Orange and blue indicate positive and negative activation *z*-scores, respectively, while white indicates no significant activation *z*-scores. Related to Figure 7b.

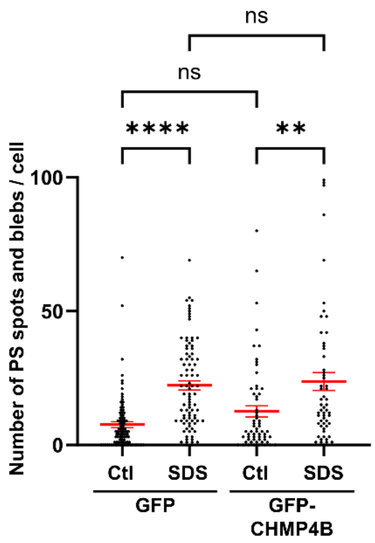

Supplementary Fig. 6. CHMP4B overexpression does not suppress the formation of PS externalizing spots/blebs after SDS treatment

WI-38 cells were treated as in Fig. 4. PS externalizing spots/blebs were stained with Annexin V-Alexa Fluor 674 before and after SDS treatment for 1 hour. Cell number: GFP-expressed untreated cells (n=91), GFP-expressed SDS-treated cells (n=89), GFP-CHMP4B-expressed untreated cells (n=61), GFP-CHMP4B-expressed SDS-treated cells (n=57). *p* value: **<0.01, ****<0.001 by One-way ANNOVA, Dunnett. Exact *p* value: Ctl vs. SDS in Control cells: <0.0001, Ctl vs. SDS in CHMP4B-expressed cells: 0.0023, Ctl in Control cells vs. Ctl in CHMP4B-expressed cells: 0.2981, SDS in Control cells vs. SDS in CHMP4B-expressed cells: 0.9571. Mean(SD).

Supplementary Table

Analysis Type:	PANTHER Overrepresentation Test (Released 20200728)						
Annotation Version and Release Date:	GO Ontology database DOI: 10.5281/zenodo.4081749 Released 2020-10-09						
Analyzed List:	Screening Hits (Saccharomyces cerevisiae)						
Reference List:	Saccharomyces cerevisiae (all genes in database)						
Test Type:	FISHER						
Correction:	FDR						
GO biological process complete	Saccharomyces cerevisiae - REFLIST (6049)	Number	expected	over/under	fold Enrichment	raw P-value	FDR
intralumenal vesicle formation (GO:0070676)	7	5	0.05	+	96.02	1.33E-08	2.33E-05
chorismate metabolic process (GO:0046417)	5	3	0.04	+	80.65	2.05E-05	3.37E-03
ATP export (GO:1904669)	17	7	0.13	+	55.35	2.32E-10	6.09E-07
endosome organization (GO:0007032)	21	6	0.16	+	38.41	3.03E-08	3.98E-05
aromatic amino acid family biosynthetic process (GO:0009073)	24	5	0.18	+	28	1.82E-06	6.37E-04
reticulophagy (GO:0061709)	15	3	0.11	+	26.88	2.84E-04	3.56E-02
protein retention in Golgi apparatus (GO:0045053)	15	3	0.11	+	26.88	2.84E-04	3.47E-02
vesicle budding from membrane (GO:0006900)	25	5	0.19	+	26.88	2.17E-06	7.13E-04
vacuolar acidification (GO:0007035)	27	5	0.2	+	24.89	3.03E-06	8.39E-04
intracellular pH reduction (GO:0051452)	27	5	0.2	+	24.89	3.03E-06	7.97E-04
pH reduction (GO:0045851)	27	5	0.2	+	24.89	3.03E-06	7.59E-04
ubiquitin-dependent protein catabolic process via the multivesicular body sortin	39	7	0.29	+	24.13	3.18E-08	3.34E-05
ATP transport (GO:0015867)	41	7	0.31	+	22.95	4.32E-08	3.79E-05
purine ribonucleotide transport (GO:0015868)	43	7	0.32	+	21.88	5.80E-08	4.36E-05
adenine nucleotide transport (GO:0051503)	43	7	0.32	+	21.88	5.80E-08	3.81E-05
regulation of cellular pH (GO:0030641)	31	5	0.23	+	21.68	5.56E-06	1.27E-03
regulation of intracellular pH (GO:0051453)	31	5	0.23	+	21.68	5.56E-06	1.22E-03
purine nucleotide transport (GO:0015865)	44	7	0.33	+	21.39	6.69E-08	3.91E-05
regulation of pH (GO:0006885)	32	5	0.24	+	21	6.39E-06	1.34E-03
late endosome to vacuole transport (GO:0045324)	64	10	0.48	+	21	8.47E-11	4.45E-07
nucleotide transport (GO:0006862)	47	7	0.35	+	20.02	1.01E-07	5.29E-05
aromatic amino acid family metabolic process (GO:0009072)	37	5	0.28	+	18.17	1.21E-05	2.28E-03
cellular monovalent inorganic cation homeostasis (GO:0030004)	40	5	0.3	+	16.8	1.71E-05	2.91E-03
endosome transport via multivesicular body sorting pathway (GO:0032509)	58	7	0.43	+	16.22	3.72E-07	1.78E-04
multivesicular body sorting pathway (GO:0071985)	58	7	0.43	+	16.22	3.72E-07	1.63E-04
monovalent inorganic cation homeostasis (GO:0055067)	42	5	0.31	+	16	2.13E-05	3.39E-03
carbohydrate derivative transport (GO:1901264)	59	7	0.44	+	15.95	4.14E-07	1.68E-04
late endosome to vacuole transport via multivesicular body sorting pathway (GO	52	5	0.39	+	12.93	5.50E-05	8.26E-03
organophosphate ester transport (GO:0015748)	79	7	0.59	+	11.91	2.56E-06	7.92E-04
endomembrane system organization (GO:0010256)	96	7	0.71	+	9.8	8.60E-06	1.74E-03
endosomal transport (GO:0016197)	117	8	0.87	+	9.19	2.86E-06	8.34E-04
vesicle organization (GO:0016050)	91	6	0.68	+	8.86	6.87E-05	1.00E-02
macroautophagy (GO:0016236)	105	6	0.78	+	7.68	1.46E-04	1.91E-02
vacuolar transport (GO:0007034)	184	10	1.37	+	7.31	1.02E-06	3.85E-04
organic acid biosynthetic process (GO:0016053)	193	9	1.44	+	6.27	1.27E-05	2.30E-03
carboxylic acid biosynthetic process (GO:0046394)	193	9	1.44	+	6.27	1.27E-05	2.23E-03
organic anion transport (GO:0015711)	166	7	1.23	+	5.67	2.40E-04	3.08E-02
small molecule biosynthetic process (GO:0044283)	336	12	2.5	+	4.8	5.16E-06	1.23E-03
vesicle-mediated transport (GO:0016192)	426	13	3.17	+	4.1	1.02E-05	1.98E-03
ion transport (GO:0006811)	390	11	2.9	+	3.79	1.15E-04	1.59E-02
organic cyclic compound biosynthetic process (GO:1901362)	611	15	4.55	+	3.3	2.31E-05	3.57E-03
primary metabolic process (GO:0044238)	3065	36	22.8	+	1.58	6.91E-05	9.82E-03
cellular metabolic process (GO:0044237)	3298	37	24.53	+	1.51	1.32E-04	1.78E-02
organic substance metabolic process (GO:0071704)	3254	36	24.21	+	1.49	4.25E-04	4.96E-02
cellular process (GO:0009987)	5030	45	37.42	+	1.2	4.23E-04	5.06E-02

Supplementary Table 1. Summary of GO enrichment analysis results

Phenotype Name	Phenotype ID	% of genes with t	% of genes with the term	Fisher's Exact Test P-value	BH FDR corrected P-value	Bonferroni corrected P-value
■ development	APO:0000023	78.72% (37/47)	49.88% (3071/6157)	0.00009303	0.0004956	0.00055818
├ lifespan	APO:0000030	57.45% (27/47)	25.5% (1570/6157)	0.000004522	0.00001306	0.000117572
│├ chronological lifespan	APO:0000316	53.19% (25/47)	19.96% (1229/6157)	5.081E-07	0.000003133	1.87997E-05
│└ replicative lifespan	APO:0000317	23.4% (11/47)	7.76% (478/6157)	0.0008334	0.002	0.0308358
├ sexual cycle	APO:0000031	44.68% (21/47)	19.78% (1218/6157)	0.0001293	0.0003362	0.0033618
│└ sporulation	APO:0000041	42.55% (20/47)	16.49% (1015/6157)	0.00002422	0.00008961	0.00089614
│ └ sporulation efficiency	APO:0000044	25.53% (12/47)	6.74% (415/6157)	0.00005523	0.0001305	0.00143598
└ budding	APO:0000024	19.15% (9/47)	6.45% (397/6157)	0.003	0.006	0.078
└ budding pattern	APO:0000200	17.02% (8/47)	2.94% (181/6157)	0.00007258	0.0002066	0.00268546
■ metabolism and growth	APO:0000094	100.0% (47/47)	81.65% (5027/6157)	0.0001652	0.0004956	0.0009912
├ protein/peptide distribution	APO:0000209	51.06% (24/47)	13.11% (807/6157)	6.181E-10	1.607E-08	1.60706E-08
├ vegetative growth	APO:0000106	89.36% (42/47)	50.64% (3118/6157)	2.747E-08	2.381E-07	7.1422E-07
├ nutrient utilization	APO:0000096	82.98% (39/47)	43.07% (2652/6157)	4.264E-08	2.772E-07	1.10864E-06
│├ utilization of carbon source	APO:0000098	74.47% (35/47)	27.04% (1665/6157)	2.209E-11	3.348E-10	8.1733E-10
││└ respiratory metabolism	APO:0000102	65.96% (31/47)	19.94% (1228/6157)	1.229E-11	4.565E-11	3.1954E-10
││ └ respiratory growth	APO:0000309	61.7% (29/47)	18.26% (1124/6157)	6.732E-11	2.356E-10	4.7124E-10
│├ auxotrophy	APO:0000097	46.81% (22/47)	9.11% (561/6157)	2.715E-11	3.348E-10	1.00455E-09
│├ utilization of nitrogen source	APO:0000099	46.81% (22/47)	21.68% (1335/6157)	0.0001328	0.000351	0.0049136
│└ utilization of iron source	APO:0000157	6.38% (3/47)	0.58% (36/6157)	0.003	0.005	0.111
├ chemical compound accumulation	APO:0000095	78.72% (37/47)	41.74% (2570/6157)	3.361E-07	0.000001748	8.7386E-06
├ chemical compound excretion	APO:0000222	34.04% (16/47)	8.07% (497/6157)	5.085E-07	0.000001962	0.000013221
├ protein/peptide modification	APO:0000131	34.04% (16/47)	8.87% (546/6157)	0.000001743	0.000005665	0.000045318
├ protein/peptide accumulation	APO:0000149	36.17% (17/47)	15.53% (956/6157)	0.0007856	0.002	0.0204256
└ RNA accumulation	APO:0000224	21.28% (10/47)	7.78% (479/6157)	0.003	0.006	0.078
■ morphology	APO:0000049	80.85% (38/47)	64.17% (3951/6157)	0.021	0.042	0.126
├ cellular morphology	APO:0000050	76.6% (36/47)	56.46% (3476/6157)	0.005	0.009	0.13
│├ cell shape	APO:0000051	14.89% (7/47)	2.6% (160/6157)	0.0002291	0.0005298	0.0084767
│├ subcellular morphology	APO:0000226	72.34% (34/47)	51.53% (3173/6157)	0.005	0.008	0.185
││├ lipid particle morphology	APO:0000242	14.89% (7/47)	2.86% (176/6157)	0.0004013	0.0008026	0.0104338
││└ endomembrane system morphology	APO:0000303	68.09% (32/47)	45.18% (2782/6157)	0.002	0.004	0.052
││ └ vacuolar morphology	APO:0000059	68.09% (32/47)	42.63% (2625/6157)	0.0005526	0.001	0.0038682
│└ cell size	APO:0000052	19.15% (9/47)	9.79% (603/6157)	0.045	0.067	1
└ culture appearance	APO:0000158	31.91% (15/47)	20.09% (1237/6157)	0.065	0.099	1
└ biofilm formation	APO:0000159	25.53% (12/47)	7.84% (483/6157)	0.0002286	0.0005298	0.0084582
■ cellular processes	APO:0000066	100.0% (47/47)	94.1% (5794/6157)	0.113	0.17	0.678
├ intracellular transport	APO:0000073	53.19% (25/47)	17.04% (1049/6157)	2.132E-08	2.381E-07	5.5432E-07
│├ endocytosis	APO:0000075	34.04% (16/47)	7.13% (439/6157)	9.69E-08	7.171E-07	3.5853E-06
│├ protein transport	APO:0000129	29.79% (14/47)	6.74% (415/6157)	0.000001907	0.00001008	0.000070559
││├ vacuolar transport	APO:0000079	23.4% (11/47)	1.06% (65/6157)	5.41E-12	2.344E-11	1.4066E-10
││└ protein secretion	APO:0000078	10.64% (5/47)	2.27% (140/6157)	0.005	0.009	0.13
│└ autophagy	APO:0000074	19.15% (9/47)	3.77% (232/6157)	0.00006487	0.0002	0.00240019
│ └ mitophagy	APO:0000240	14.89% (7/47)	2.55% (157/6157)	0.0002048	0.0004437	0.0053248
├ chromosome/plasmid maintenance	APO:0000143	63.83% (30/47)	27.97% (1722/6157)	5.282E-07	0.000001962	1.37332E-05
│├ telomere length	APO:0000144	36.17% (17/47)	5.7% (351/6157)	5.145E-10	4.759E-09	1.90365E-08
│└ transposable element transposition	APO:0000047	23.4% (11/47)	7.42% (457/6157)	0.0005755	0.001	0.0212935
└ stress resistance	APO:0000080	100.0% (47/47)	91.9% (5658/6157)	0.03	0.052	0.78
├ temperature sensitive growth	APO:0000092	80.85% (38/47)	30.13% (1855/6157)	1.192E-12	4.41E-11	4.4104E-11
│└ heat sensitivity	APO:0000147	80.85% (38/47)	28.75% (1770/6157)	2.421E-13	1.574E-12	6.2946E-12
├ toxin resistance	APO:0000215	57.45% (27/47)	25.55% (1573/6157)	0.000004646	0.00002149	0.000171902
│└ killer toxin resistance	APO:0000081	19.15% (9/47)	9.24% (569/6157)	0.037	0.057	0.962
├ resistance to enzymatic treatment	APO:0000193	25.53% (12/47)	5.73% (353/6157)	0.00001148	0.0000472	0.00042476
├ thermotolerance	APO:0000093	40.43% (19/47)	16.05% (988/6157)	0.00006129	0.0002	0.00226773
│└ innate thermotolerance	APO:0000334	40.43% (19/47)	15.67% (965/6157)	0.00004414	0.0001148	0.00114764
├ freeze-thaw resistance	APO:0000241	10.64% (5/47)	1.25% (77/6157)	0.0003541	0.0007707	0.0131017
├ desiccation resistance	APO:0000326	36.17% (17/47)	16.0% (985/6157)	0.000908	0.002	0.033596
├ radiation resistance	APO:0000084	19.15% (9/47)	6.33% (390/6157)	0.003	0.005	0.111
│└ UV resistance	APO:0000085	14.89% (7/47)	5.64% (347/6157)	0.016	0.026	0.416
└ resistance to chemicals	APO:0000087	100.0% (47/47)	87.07% (5361/6157)	0.003	0.005	0.111
├ alkaline pH resistance	APO:0000202	42.55% (20/47)	2.6% (160/6157)	3.062E-19	7.961E-18	7.9612E-18
├ metal resistance	APO:0000090	61.7% (29/47)	12.05% (742/6157)	1.692E-15	2.2E-14	4.3992E-14
├ ionic stress resistance	APO:0000205	34.04% (16/47)	2.66% (164/6157)	8.88E-14	7.696E-13	2.3088E-12
├ acid pH resistance	APO:0000201	42.55% (20/47)	6.4% (394/6157)	3.572E-12	1.857E-11	9.2872E-11
├ osmotic stress resistance	APO:0000082	42.55% (20/47)	7.71% (475/6157)	9.6E-11	3.12E-10	2.496E-09
│└ hyperosmotic stress resistance	APO:0000204	42.55% (20/47)	7.28% (448/6157)	3.448E-11	2.356E-10	2.4136E-10
└ oxidative stress resistance	APO:0000083	46.81% (22/47)	18.55% (1142/6157)	0.00001025	0.00002961	0.0002665

Supplementary Table 2. Summary of *mod*PhEA analysis results

The genes and gene functions associated with "replicative lifespan" are highlighted in blue.

Gene	Systematic Name	Function
DID4	YKL002W	ESCRT
SNF7	YLR025W	
STP22	YCL008C	
VPS20	YMR077C	
VPS24	YKL041W	
VPS25	YJR102C	
VPS36	YLR417W	
VPS4	YPR173C	
VMA1	YDL185W	V-ATPase
VMA13	YPR036W	
VMA21	YGR105W	
VMA5	YKL080W	
VMA9	YCL005W-A	
VPH2	YKL119C	
AAT2	YLR027C	Amino acid biosynthesis/sensor/metabolism
ARO1	YDR127W	
ARO2	YGL148W	
ARO7	YPR060C	
TRP4	YDR354W	
TRP5	YGL026C	
DEF1	YKL054C	DNA replication/damage/repair
FYV6	YNL133C	
RFA2	YNL312W	
SEN1	YLR430W	
ACB1	YGR037C	Lipid
CHO1	YER026C	
ERG2	YMR202W	
ERG3	YLR056W	
RPB3	YIL021W	RNA helicase/polymerase
PRP22	YER013W	
PRP43	YGL120C	
ISA1	YLL027W	Mitochondria
ISA2	YPR067W	
MRPL51	YPR100w	
VPS16	YPL045W	SNARE/HOPS
PEP3	YLR148W	
SGD1	YLR336C	Ribosome
UTP5	YDR398W	
PHO85	YPL031C	CDK
GAS1	YMR307W	Cell wall
STH1	YIL126W	Chromatin remodeling
SWA2	YDR320C	Endocytosis/Exocytosis
VPS34	YLR240W	PI3-kinase
TPD3	YAL016W	PP2A
PTC1	YDL006W	PP2C
ARF1	YDL192W	Ras GTPase
CCR4	YAL021C	Transcription
KCS1	YDR017C	Inositol/Vacuole

Supplementary Table 3. 18 out of 48 screening hits are reported to have altered replicative lifespan

Supplementary Table 4. SASP factors identified in the significant genes expressed in PMD-Sen, Ca^{2+}-Sen, and DDR-Sen

Supplementary Table 5. Overlapped pathways between cutaneous wound and PMD-Sen/DDR-Sen in the significantly differentially regulated pathways

Supplementary Table 6. Overlapped pathways between cutaneous wound and PMD-Sen/DDR-Sen in the top 146 pathways regardless of its *z*-score

Supplementary Table 7. Yeast strains used in this study

Supplementary Table 8. Antibodies used in this study

Supplementary Table 9. Primer sequences used for qPCR analyses

*Supplementary Table 4-9 were provided in the .xlsx format.
