# Supplementary material for: Plasma membrane damage limits replicative lifespan in yeast and induces premature senescence in human fibroblasts
Source: Nat Aging. 2024 Feb 22;4(3):319–35. doi: 10.1038/s43587-024-00575-6 (PMC10950784; doi:10.1038/s43587-024-00575-6)
Supplement: Supplementary file 2 — Reporting Summary [file 43587_2024_575_MOESM2_ESM.pdf]

nature portfolio

Corresponding author(s): Keiko Kono

Last updated by author(s): Jan 19, 2024

Reporting Summary

Nature Portfolio wishes to improve the reproducibility of the work that we publish. This form provides structure for consistency and transparency in reporting. For further information on Nature Portfolio policies, see our Editorial Policies and the Editorial Policy Checklist.

Statistics

For all statistical analyses, confirm that the following items are present in the figure legend, table legend, main text, or Methods section.

n/a	Confirmed	
☐	☒	The exact sample size (*n*) for each experimental group/condition, given as a discrete number and unit of measurement
☐	☒	A statement on whether measurements were taken from distinct samples or whether the same sample was measured repeatedly
☐	☒	The statistical test(s) used AND whether they are one- or two-sided<br>*Only common tests should be described solely by name; describe more complex techniques in the Methods section.*
☒	☐	A description of all covariates tested
☐	☒	A description of any assumptions or corrections, such as tests of normality and adjustment for multiple comparisons
☐	☒	A full description of the statistical parameters including central tendency (e.g. means) or other basic estimates (e.g. regression coefficient) AND variation (e.g. standard deviation) or associated estimates of uncertainty (e.g. confidence intervals)
☐	☒	For null hypothesis testing, the test statistic (e.g. *F*, *t*, *r*) with confidence intervals, effect sizes, degrees of freedom and *P* value noted<br>*Give P values as exact values whenever suitable.*
☒	☐	For Bayesian analysis, information on the choice of priors and Markov chain Monte Carlo settings
☒	☐	For hierarchical and complex designs, identification of the appropriate level for tests and full reporting of outcomes
☒	☐	Estimates of effect sizes (e.g. Cohen's *d*, Pearson's *r*), indicating how they were calculated

Our web collection on statistics for biologists contains articles on many of the points above.

Software and code

Policy information about availability of computer code

Data collection	[Light microscopic observation]<br>Celldiscoverer 7 (Zeiss ZEN2.6), LSM 780 NLO (Zeiss ZEN2.3), AxioObserver7 (Zeiss ZEN 3.1). BZ-9000 (Keyence), Axio Observer z1 (Zeiss ZEN2.3), SP8 (Leica LAS X), A1R (Nikon NIS-Elements ver6.0), DMiL (Leica) equipped with EOS kiss X4 (Cannon).<br><br>[Electron microscopic observation]<br>JEM-1011J (JEOL), JEM-1400Plus (JEOL) and MI4000L (Hitachi High-Tech)<br><br>[Western blotting]<br>ChemiDoc Touch MP (Bio-Rad)<br><br>[RNA sequencing]<br>Novaseq6000 (Illumina) for 2x 150bp yielding at least 20 M reads per sample<br><br>[Imaging flowcytometry]<br>Amnis Imagestream x Mk II (Millipore) and FCS express (De novo software).<br><br>[Gene expression analysis]<br>QuantStudio 1 (Thermo Fischer Scientific)
Data analysis	[Light microscopic data]<br>Fiji/ImageJ v1.5 or later

[Electron microscopic data]
Fiji/ImageJ and AMIRA v5.6 (FEI Visualization Science Group)

[Western blotting]
BioRad ImageLab 6.0

[RNA sequencing]
Trim_galore, HISAT2 to the human GRCh38 reference genome, htseq-count, DEseq2, Ingenuity Pathway Analysis (IPA) (Ingenuity Systems)

[Statistical data analysis]
GraphPad Prism9, SPSS software version 21.0 (IBM Corp.)

For manuscripts utilizing custom algorithms or software that are central to the research but not yet described in published literature, software must be made available to editors and reviewers. We strongly encourage code deposition in a community repository (e.g. GitHub). See the Nature Portfolio guidelines for submitting code & software for further information.

Data

Policy information about availability of data

All manuscripts must include a data availability statement. This statement should provide the following information, where applicable:

- Accession codes, unique identifiers, or web links for publicly available datasets
- A description of any restrictions on data availability
- For clinical datasets or third party data, please ensure that the statement adheres to our policy

The RNAseq dataset generated and analyzed during the current study is available in the NCBI GEO repository, https://www.ncbi.nlm.nih.gov/geo/query/acc.cgi?acc=GSE222400

The rest of the data generated or analyzed during this study are included in this published article (and its supplementary information files).

Externally generated data can be obtained from the following resources:
https://www.ncbi.nlm.nih.gov/geo/query/acc.cgi?acc=GSE141814
http://www.saspatlas.com/

Research involving human participants, their data, or biological material

Policy information about studies with human participants or human data. See also policy information about sex, gender (identity/presentation), and sexual orientation and race, ethnicity and racism.

Reporting on sex and gender	N/A
Reporting on race, ethnicity, or other socially relevant groupings	N/A
Population characteristics	N/A
Recruitment	N/A
Ethics oversight	N/A

Note that full information on the approval of the study protocol must also be provided in the manuscript.

Field-specific reporting

Please select the one below that is the best fit for your research. If you are not sure, read the appropriate sections before making your selection.

☒ Life sciences ☐ Behavioural & social sciences ☐ Ecological, evolutionary & environmental sciences

For a reference copy of the document with all sections, see nature.com/documents/nr-reporting-summary-flat.pdf

Life sciences study design

All studies must disclose on these points even when the disclosure is negative.

Sample size	No explicit sample size calculation was performed. We followed community norms and the sample sizes are similar to those reported in previous publications (Ref. 15, 22). Exact sample sizes are indicated in the corresponding figure legends and supplementary figure legends. In Biochemical and spectrometric experiments, each experiment was independently repeated multiple times.

Data exclusions	No data were excluded from the analysis.
Replication	In principle, at least three independent experiments for each assay were performed to verify the reproducibility of the findings. mRNA-seq was performed with two independent samples. Exact sample sizes / number of independent experiments are indicated in the corresponding figure legends, supplementary figure legends and supplementary data.
Randomization	All cell culture experiments were randomly assigned to experimental conditions. For microscopic images, the image fields of immunofluorescence and TEM observations, SA-β-gal staining were randomly acquired.
Blinding	The majority of experiments were not blinded because the same investigators performed the experiments and analyzed the data. In addition, blinding during data collection was not possible for the majority of these studies because the experimental conditions caused significant phenotypic changes between the groups (untreated vs. treated (senescent), fluorescent marker differences, etc.). For RNA-seq, we anonymized the data at the time of data collection and only grouped and analyzed the data blindly.

Reporting for specific materials, systems and methods

We require information from authors about some types of materials, experimental systems and methods used in many studies. Here, indicate whether each material, system or method listed is relevant to your study. If you are not sure if a list item applies to your research, read the appropriate section before selecting a response.

Materials & experimental systems

n/a	Involved in the study	
☐	☒	Antibodies
☐	☒	Eukaryotic cell lines
☒	☐	Palaeontology and archaeology
☒	☐	Animals and other organisms
☒	☐	Clinical data
☒	☐	Dual use research of concern
☒	☐	Plants

Methods

n/a	Involved in the study	
☒	☐	ChIP-seq
☐	☒	Flow cytometry
☒	☐	MRI-based neuroimaging

Antibodies

Antibodies used

Rabbit Anti-CHMP4A HPA068473 Sigma-Aldrich (Merck) PRID: AB_2685994, 1:1000
Mouse Anti-p53 [DO-1] sc-126 Santa Cruz Biotechnologies PRID: AB_628082, 1:1000
Rabbit Anti-Phospho-p53 (Ser15) 9284 Cell Signaling Technology PRID: AB_331464, 1:1000
Rabbit Anti-Phospho-p53 (Ser33) 2526 Cell Signaling Technology PRID: AB_331509, 1:1000
Mouse Anti-p21 Waf1/Cip1 [F-5] sc-6246 Santa Cruz Biotechnologies PRID: AB_628073, 1:1000
Rabbit Anti-p21 [EPR362] ab109520 Abcam PRID: AB_10860537, 1:5000
Mouse Anti-p16 (JC8) sc-56330 Santa Cruz Biotechnologies PRID: AB_785018, 1:200
Rabbit Anti-CDKN2A/p16INK4a [EPR1473] ab108349 Abcam PRID: AB_10858268, 1:1000
Rabbit Anti-Phospho-ATM (S1981) AF1655 R&D Systems PRID: AB_2062935 1:200
Rabbit Anti-ATM (phospho S1981) [EP1890Y] ab81292 Abcam PRID: AB_1640207, 1:15000
Mouse Anti-ATM [2C1(1A1)] ab78 Abcam PRID: AB_306089, 1:1000
Mouse Anti-Phospho-H2A.X(Ser139) [JBW301] 05-636 Sigma-Aldrich (Merck) PRID: AB_309864, 1:1000
Mouse Anti-p-Histone H2A.X (Ser 139) sc-517348 Santa Cruz Biotechnologies PRID: AB_2783871, 1:1000
Rabbit Anti-H2A.X ab11175 Abcam PRID: AB_297814, 1:2000
Rabbit Anti-Histone H2A.X (D17A3) 7631 Cell Signaling Technology PRID: AB_10860771, 1:1000
Rabbit Anti-Phospho-Chk1(Ser345) [133D3] 2348 Cell Signaling Technology PRID: AB_331212, 1:2000
Mouse Anti-Chk1 [DCS310] C9358 Sigma-Aldrich (Merck) PRID: AB_259159, 1:1000
Rabbit Anti-GFP A-6455 Thermo Fisher Scientific PRID: AB_221570, 1:1000
Rabbit Anti-GAPDH (D16H11) 5174 Cell Signaling Technology PRID: AB_10622025, 1:5000
Mouse Anti-beta Actin antibody [AC-15] ab6276 Abcam PRID: AB_2223210, 1:5000
Goat Anti-Rabbit IgG (H+L) Alexa Fluor Plus 488 A32731 Thermo Fisher Scientific PRID: AB_2633280, 1:200
Goat Anti-Rabbit IgG (H) Alexa Fluor 647 A55055 Thermo Fisher Scientific PRID: AB_2921063, 1:250
Sheep Anti-mouse HRP IgG NA9310 Cytiva PRID: AB_772193, 1:5000
Donkey Anti-Rabit HRP IgG NA9340 Cytiva PRID: AB_772191, 1:5000
Goat Anti-rabbit IgG, HRP-linked 7074 Cell Signaling Technology PRID: AB_2099233, 1:5000
Horse Anti-mouse IgG, HRP-linked 7076 Cell Signaling Technology PRID: AB_330924, 1:5000
hFAB Rhodamine Anti-Actin #12004164 Bio-Rad PRID: AB_2861334, 1:2000

Validation

The validation of the antibodies was performed by the indicated manufacturers and supported by the publication indicated on the manufacturer's website. Some of the antibodies were additionally validated by us using the corresponding siRNAs or overexpression of GFP-tagged protein by Western blotting / immunofluorescence staining,

CHMP4A (Sigma HPA068473)
(IHC https://www.sigmaaldrich.com/JP/ja/product/sigma/hpa068473)
This antibody was further validated using siRNAs and overexpression of GFP-tagged protein by Western blotting and fluorescent

immunostaining

p53 [DO-1] (Santa Cruz sc-126)
(WB, IP, IF, IHC, FCM https://www.scbt.com/p/p53-antibody-do-1)
This antibody was further validated using siRNAs by Western blotting.

Phospho-p53 (Ser15) (CST 9284)
(WB, IP https://www.cellsignal.jp/products/primary-antibodies/phospho-p53-ser15-antibody/9284)

Phospho-p53 (Ser33) (CST 2526)
(WB, IHC https://www.cellsignal.jp/products/primary-antibodies/phospho-p53-ser33-antibody/2526)

p21 Waf1/Cip1 [F-5] (Santa Cruz sc-6246)
(WB, IP, IF, IHC,FCM https://www.scbt.com/p/p21-antibody-f-5)
This antibody was further validated by overexpression of GFP-tagged protein by Western blotting.

p21 [EPR362] (Abcam ab109520)
(WB, IF, IHC, FCM https://www.abcam.com/en-mx/products/primary-antibodies/p21-antibody-epr362-ab109520)
This antibody was further validated by overexpression of GFP-tagged protein by Western blotting.

p16 (JC8) (Santa Cruz sc-56330)
(WB, IP, IF, IHC, ELISA https://www.scbt.com/p/p16-antibody-jc8)

CDKN2A/p16INK4a [EPR1473] (Abcam ab108349)
(WB, IP, IHC https://www.abcam.com/en-ee/products/primary-antibodies/cdkn2a-p16ink4a-antibody-epr1473-c-terminal-ab108349)
We further validated this antibody by overexpressing GFP-tagged protein by Western blotting and confirmed that this antibody reacts only with p16 and not with p15.

Phospho-ATM (S1981) (R&D AF1655)
(WB https://www.rndsystems.com/products/human-mouse-rat-phospho-atm-s1981-antibody_af1655)

ATM (phospho S1981) [EP1890Y] (Abcam ab81292)
(WB, IP, IHC, FCM https://www.abcam.com/en-at/products/primary-antibodies/atm-phospho-s1981-antibody-ep1890y-ab81292)

ATM [2C1(1A1)] (Abcam Ab78)
(WB, IP, IHC, FCM https://www.abcam.com/en-za/products/primary-antibodies/atm-antibody-2c1-1a1-bsa-and-azide-free-ab78)

Phospho-H2A.X(Ser139) [JBW301] (Sigma 05-636)
(WB, IP, IF, IHC https://www.emdmillipore.com/US/en/product/Anti-phospho-Histone-H2A.X-Ser139-Antibody-clone-JBW301,MM_NF-05-636?bd=1)

p-Histone H2A.X (Ser 139) (Santa Cruz sc-517348)
(WB, IF https://www.scbt.com/p/p-histone-h2a-x-antibody-ser-139)

H2A.X (Abcam ab11175)
(WB, IHC https://www.abcam.com/products/primary-antibodies/histone-h2ax-antibody-ab11175.html)

Histone H2A.X (D17A3) (CST 7631)
(WB, IP, IF, IHC, FCM https://www.cellsignal.jp/products/primary-antibodies/histone-h2a-x-d17a3-xp-rabbit-mab/7631)

Phospho-Chk1(Ser345) [133D3] (CST 2348)
(WB, IF, FCM https://www.cellsignal.jp/products/primary-antibodies/phospho-chk1-ser345-133d3-rabbit-mab/2348)

Chk1 [DCS310] (Sigma C9358)
(WB, IP, IHC https://www.sigmaaldrich.com/US/en/product/sigma/c9358)

GFP (Thermo Fisher A-6455)
(WB, IP, IF, IHC, FCM, ELSA https://www.thermofisher.com/antibody/product/GFP-Antibody-Polyclonal/A-6455)

GAPDH (D16H11) (CST 5174)
(WB, IF, IHC https://www.cellsignal.com/products/primary-antibodies/gapdh-d16h11-xp-rabbit-mab/5174?language=en)

beta Actin [AC-15] (Abcam ab6276)
(WB, IF https://www.abcam.com/en-at/products/primary-antibodies/beta-actin-antibody-ac-15-ab6276)

Rabbit IgG (H+L) Alexa Fluor Plus 488 (Thermo Fisher A32731)
(WB, IF https://www.thermofisher.com/antibody/product/Goat-anti-Rabbit-IgG-H-L-Highly-Cross-Adsorbed-Secondary-Antibody-Polyclonal/A32731)

Rabbit IgG (H) Alexa Fluor 647 (Thermo Fisher A55055)
(WB, IF, FCM https://www.thermofisher.com/antibody/product/Goat-anti-Rabbit-IgG-Heavy-chain-Secondary-Antibody-Recombinant-Polyclonal/A55055)

Mouse HRP IgG (Cytiva NA9310)
(WB https://www.cytivalifesciences.com/en/us/shop/protein-analysis/blotting-and-detection/blotting-standards-and-reagents/

amersham-ecl-hrp-conjugated-antibodies-p-06260)

Rabit HRP IgG (Cytiva NA9340)
(WB https://www.cytivalifesciences.com/en/us/shop/protein-analysis/blotting-and-detection/blotting-standards-and-reagents/amersham-ecl-hrp-conjugated-antibodies-p-06260)

Rabbit IgG, HRP-linked (CST 7074)
(WB https://www.cellsignal.com/products/secondary-antibodies/anti-rabbit-igg-hrp-linked-antibody/7074?country=USA)

Mouse IgG, HRP-linked (CST 7076)
(WB https://www.cellsignal.com/products/secondary-antibodies/anti-mouse-igg-hrp-linked-antibody/7076)

Rhodamine Anti-Actin (Bio-Rad #12004164)
(WB https://www.bio-rad.com/en-jp/sku/12004164-hfab-rhodamine-anti-actin-primary-antibody-40-ul?ID=12004164)

Eukaryotic cell lines

Policy information about cell lines and Sex and Gender in Research

Cell line source(s)

HeLa cell line, endocervical adenocarcinoma, female, from RIKEN BRC Cat# RCB0007, RRID:CVCL_0030
WI-38 cells, fibroblast, female, PDL36.6 from RIKEN BRC Cat# RCB0702, RRID:CVCL_0579
WI-38 cells, fibroblast, female, PDL32.2 from JCRB cell bank Cat# IFO50075, RRID:CVCL_0579
BJ cells, fibroblast, male, PDL24.0 from ATCC, Cat# CRL-2522, RRID:CVCL_3653
HCA2, fibroblast we previously described in DOI: 10.1073/pnas.92.10.4352, male, PDL16.0. This cell is not commercially available.

Authentication

HeLa cell line (RIKEN: morphology, PCR assays with species-specific primers, Karyotyping), WI-38 cells (RIKEN: morphology, STR profiling, Isozyme analysis for the Identification of species, Karyotyping), WI-38 cells (JCRB: Isozyme analysis for the Identification of species) and BJ cells (ATCC: STR profiling) were authenticated by the supplier.
HCA2 cells were authenticated by the authors in DOI: 10.1073/pnas.92.10.4352, and no further authentication was performed in the laboratory.

Mycoplasma contamination

All cells have been confirmed mycoplasma negative by the distributor. In addition, all cells are periodically stained by Hoechst 33342 to confirm that they are mycoplasma negative.

Commonly misidentified lines
(See ICLAC register)

No misidentified lines were used in this study.

Plants

Seed stocks

Report on the source of all seed stocks or other plant material used. If applicable, state the seed stock centre and catalogue number. If plant specimens were collected from the field, describe the collection location, date and sampling procedures.

Novel plant genotypes

Describe the methods by which all novel plant genotypes were produced. This includes those generated by transgenic approaches, gene editing, chemical/radiation-based mutagenesis and hybridization. For transgenic lines, describe the transformation method, the number of independent lines analyzed and the generation upon which experiments were performed. For gene-edited lines, describe the editor used, the endogenous sequence targeted for editing, the targeting guide RNA sequence (if applicable) and how the editor was applied.

Authentication

Describe any authentication procedures for each seed stock used or novel genotype generated. Describe any experiments used to assess the effect of a mutation and, where applicable, how potential secondary effects (e.g. second site T-DNA insertions, mosiacism, off-target gene editing) were examined.

Flow Cytometry

Plots

Confirm that:

☒ The axis labels state the marker and fluorochrome used (e.g. CD4-FITC).

☒ The axis scales are clearly visible. Include numbers along axes only for bottom left plot of group (a 'group' is an analysis of identical markers).

☐ All plots are contour plots with outliers or pseudocolor plots.

☐ A numerical value for number of cells or percentage (with statistics) is provided.

Methodology

Sample preparation

WI-38 cells cultured in DAPI containing media with or without SDS. Cells were fixed with 4% PFA before resuspended in flow cytometry buffer.

Instrument

ImageStream x MKII (Millipore)

Software	FCS express (De novo software)
Cell population abundance	Samples were not sorted in this study
Gating strategy	We did not gate any data.

☐ Tick this box to confirm that a figure exemplifying the gating strategy is provided in the Supplementary Information.
