# Supplementary material for: Plasma membrane damage limits replicative lifespan in yeast and induces premature senescence in human fibroblasts
Source: Nat Aging. 2024 Feb 22;4(3):319–35. doi: 10.1038/s43587-024-00575-6 (PMC10950784; doi:10.1038/s43587-024-00575-6)

Unprocessed source data of western Blots

Figure 3e

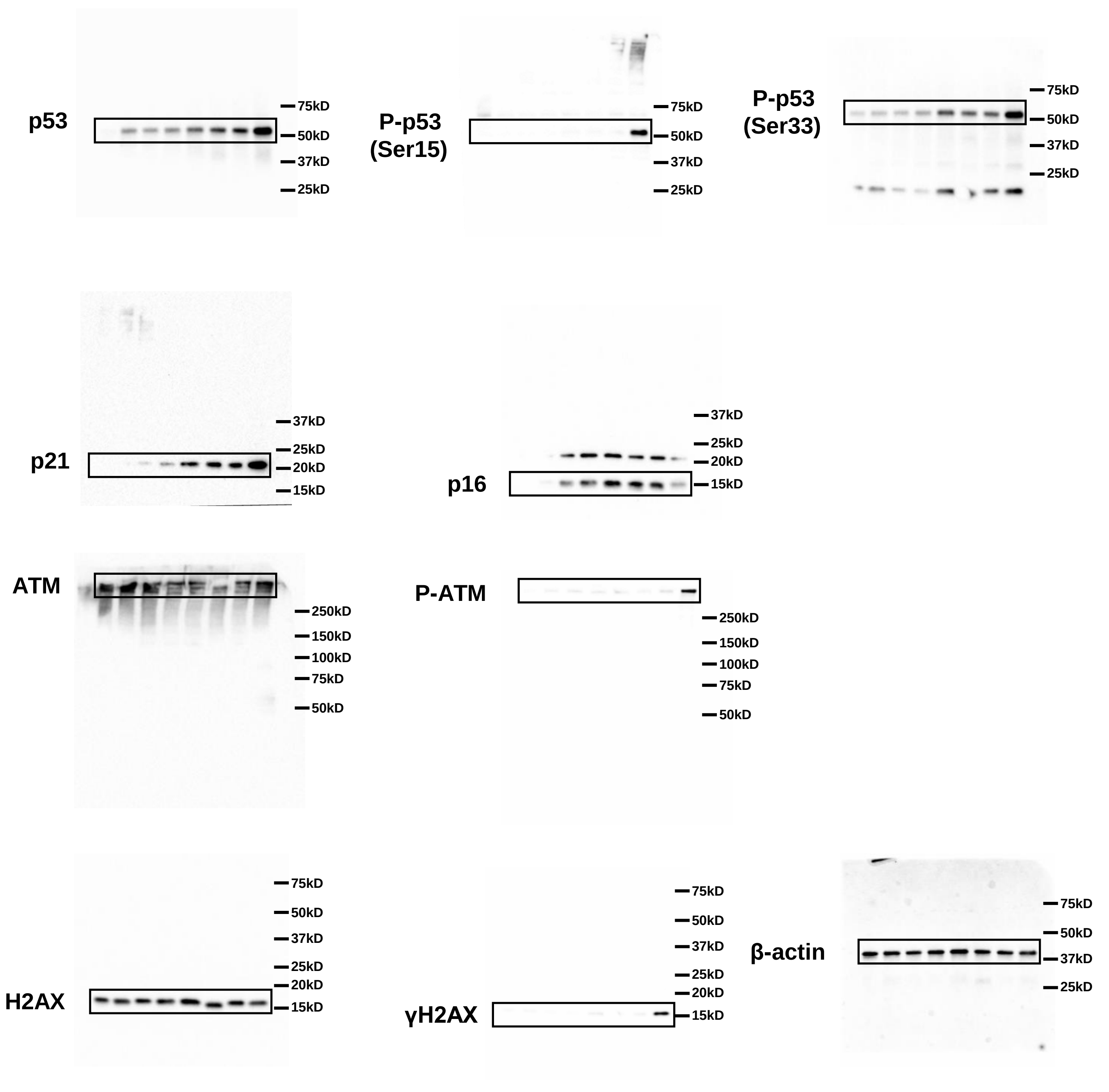

Figure 3i

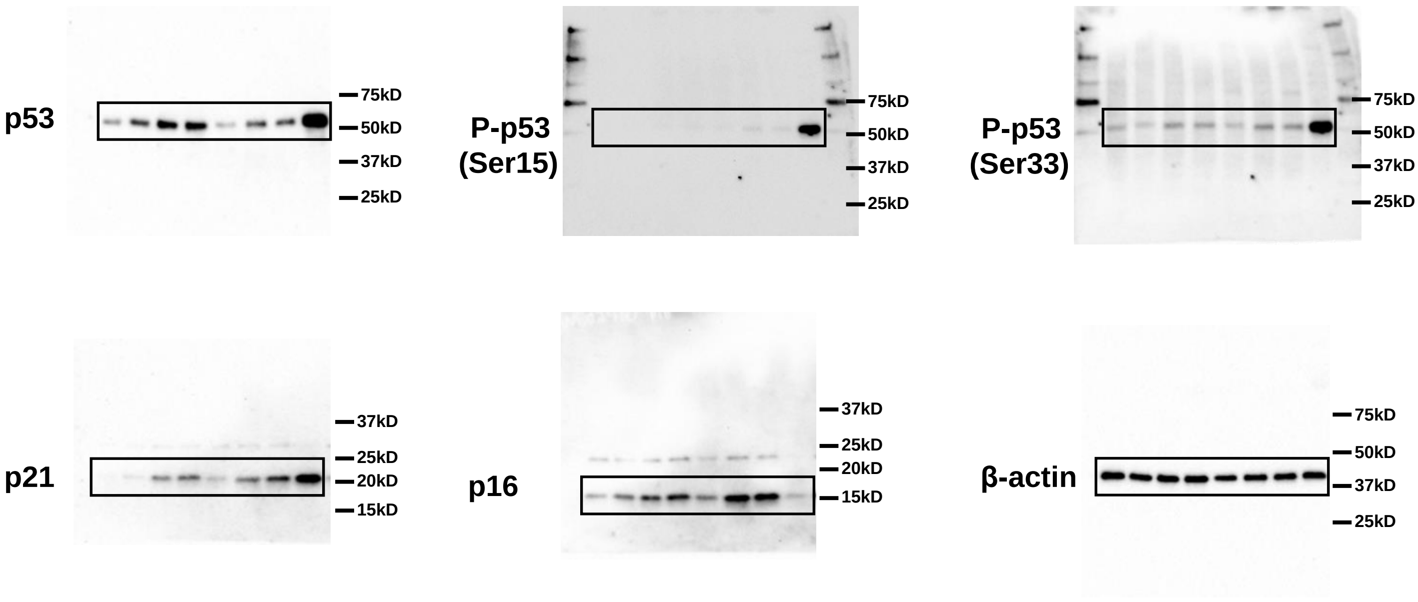

Figure 4c

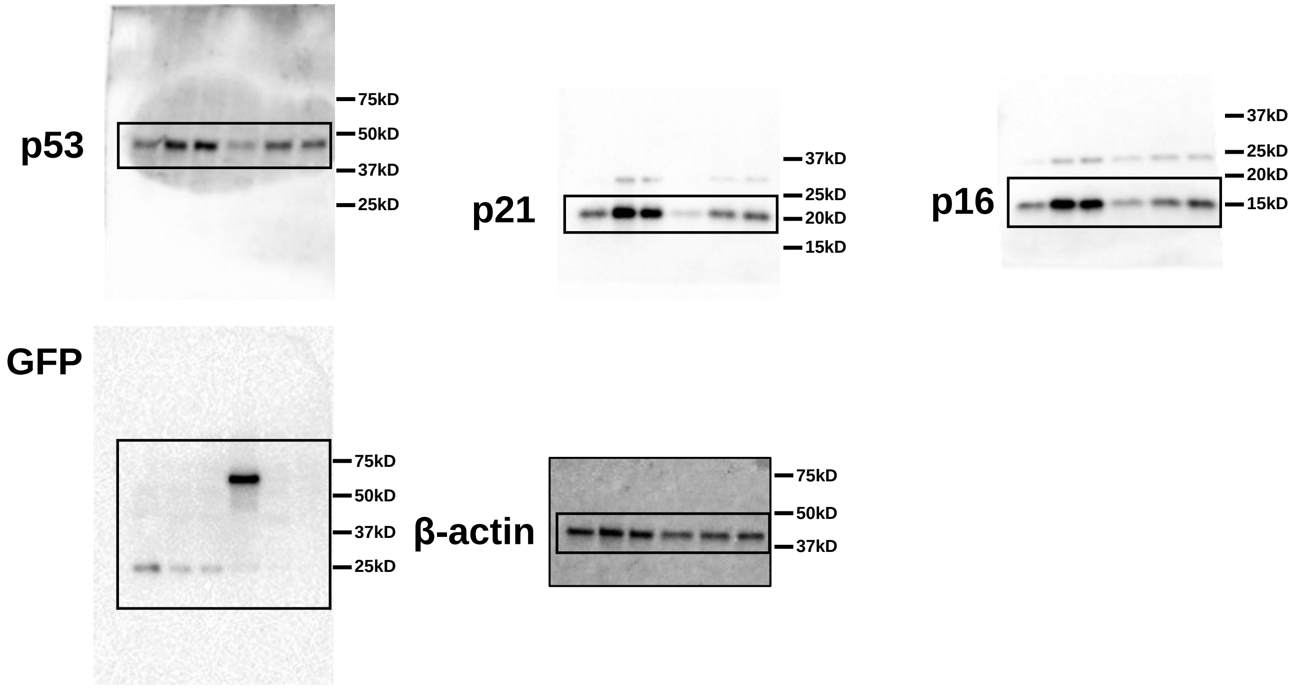

Figure 5b

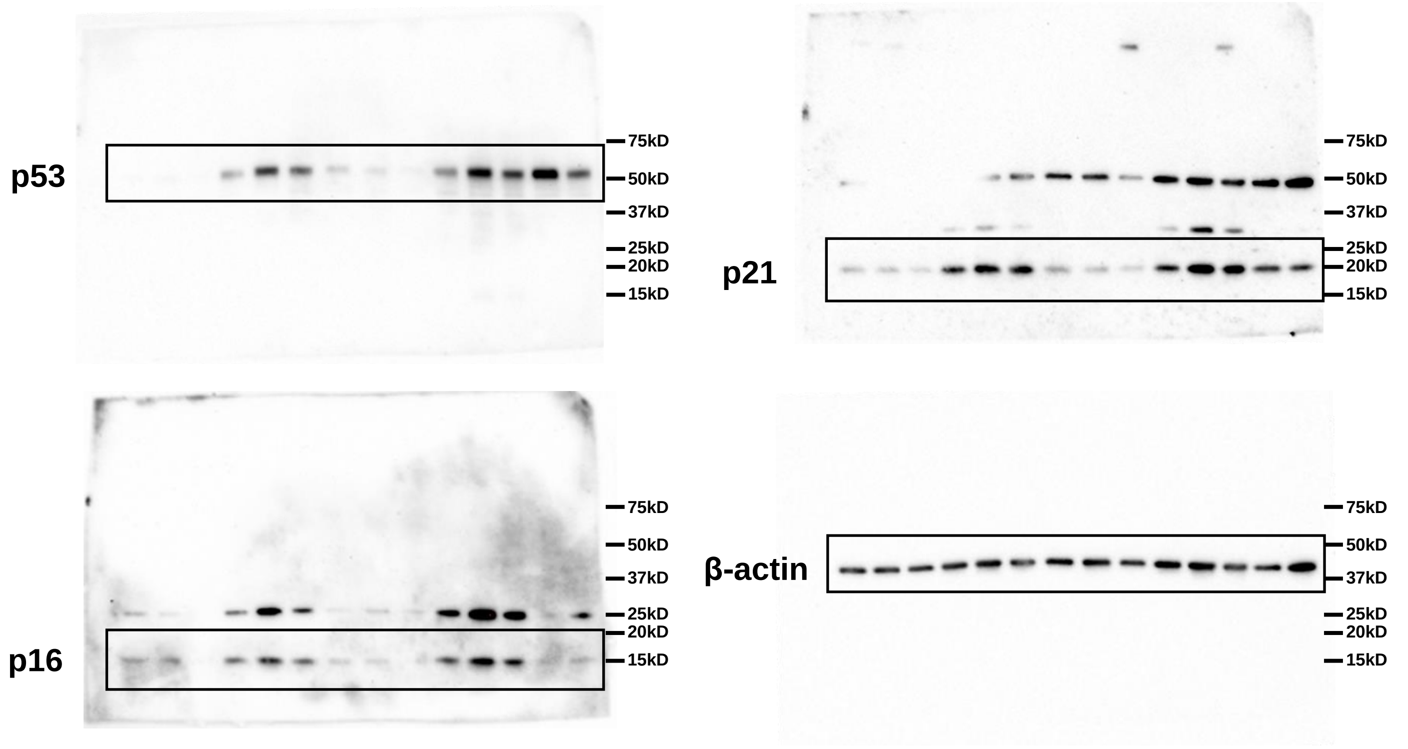

Figure 5c

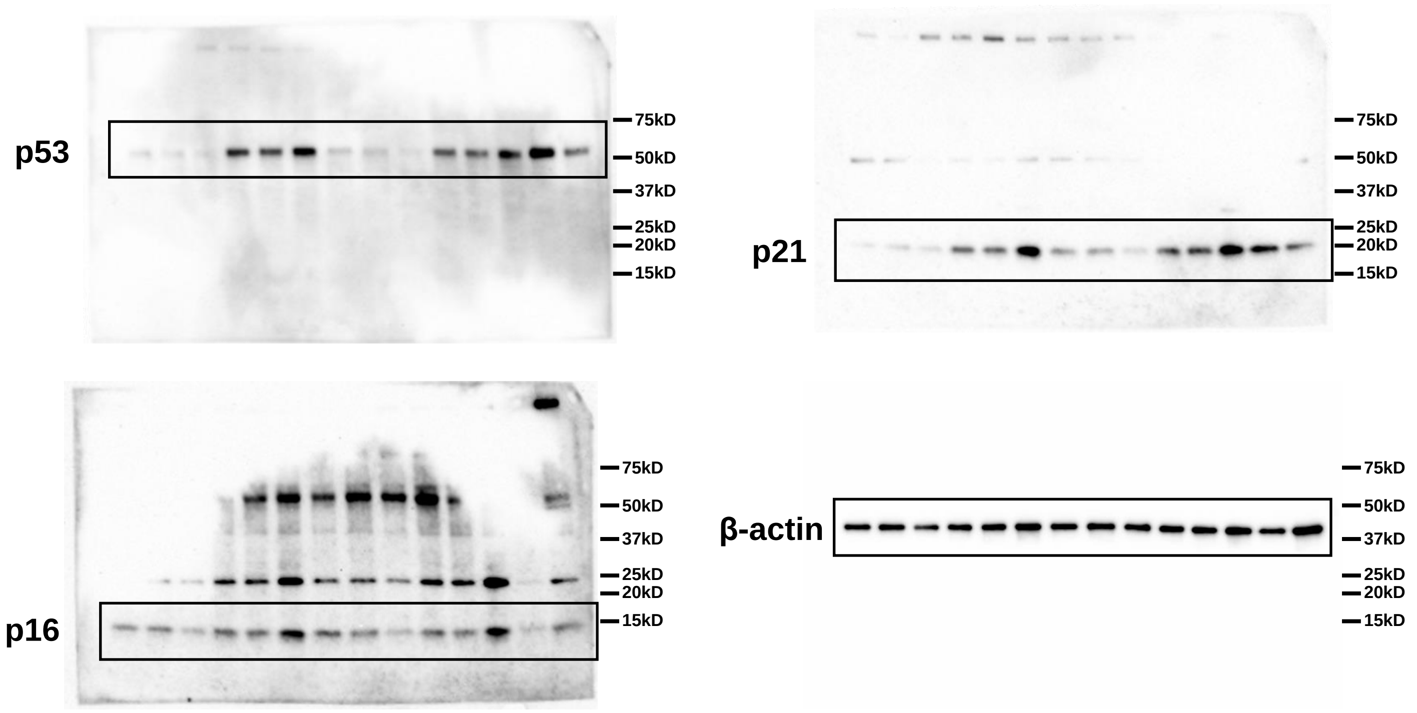

Figure 5d

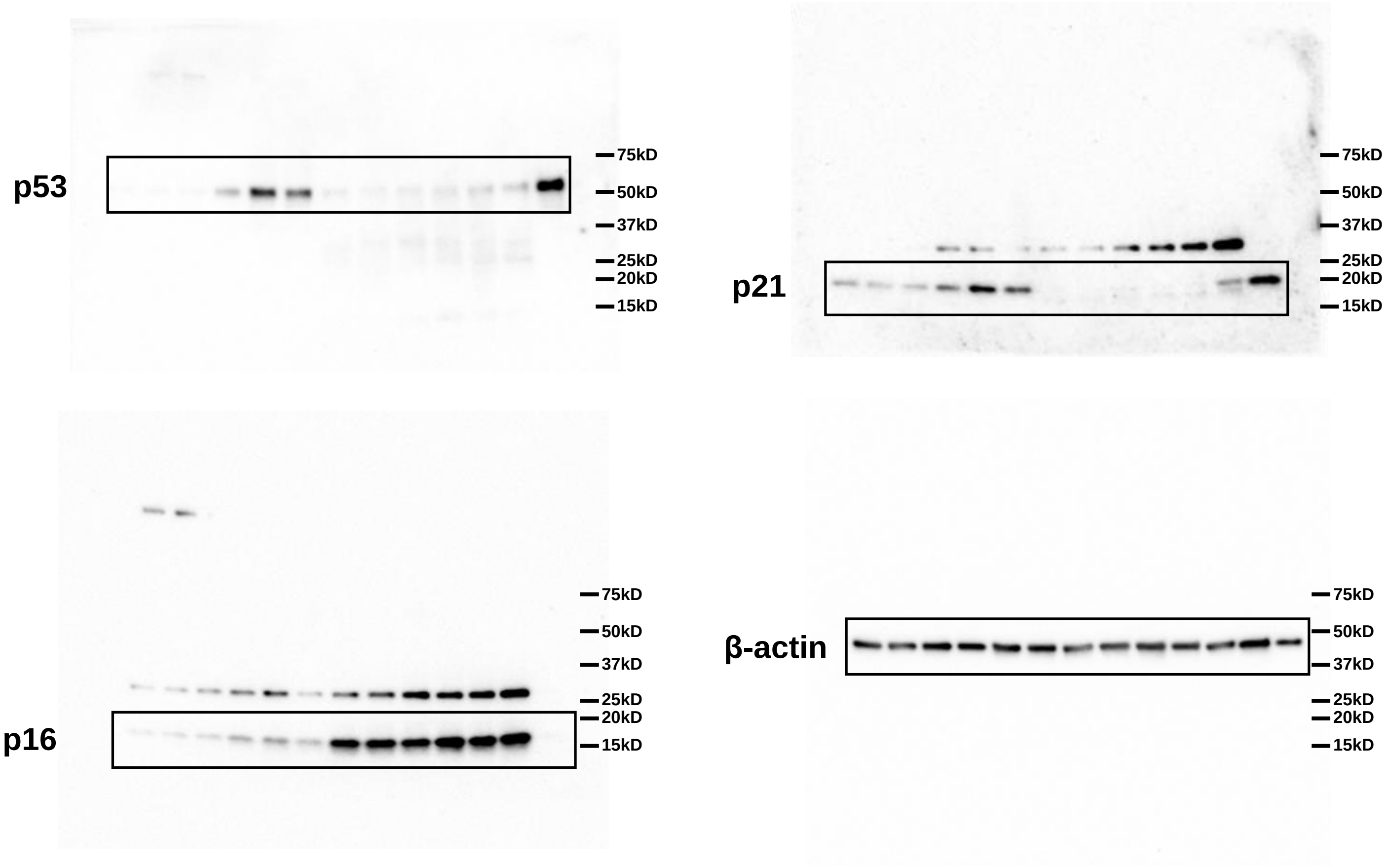

Figure 6c

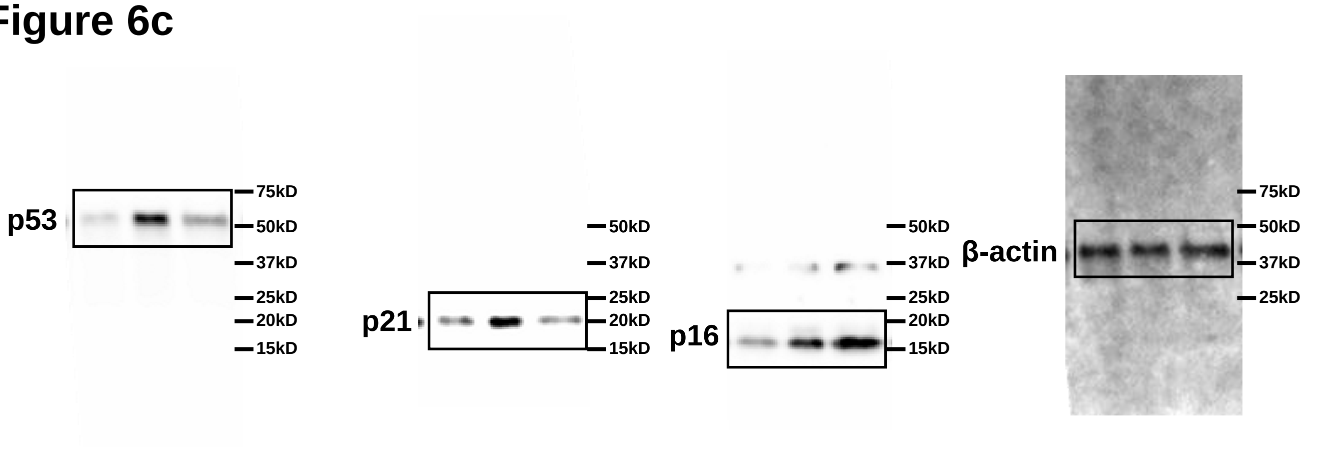

Figure 6f

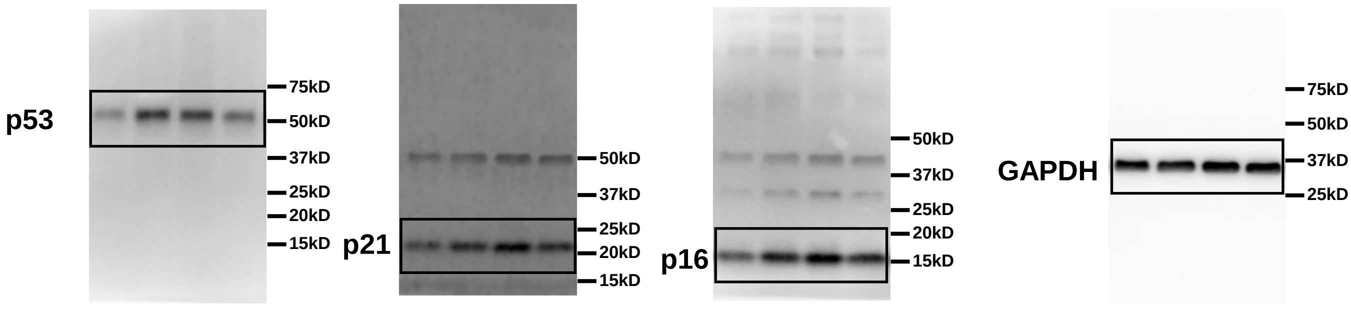

Extended data Figure 6e

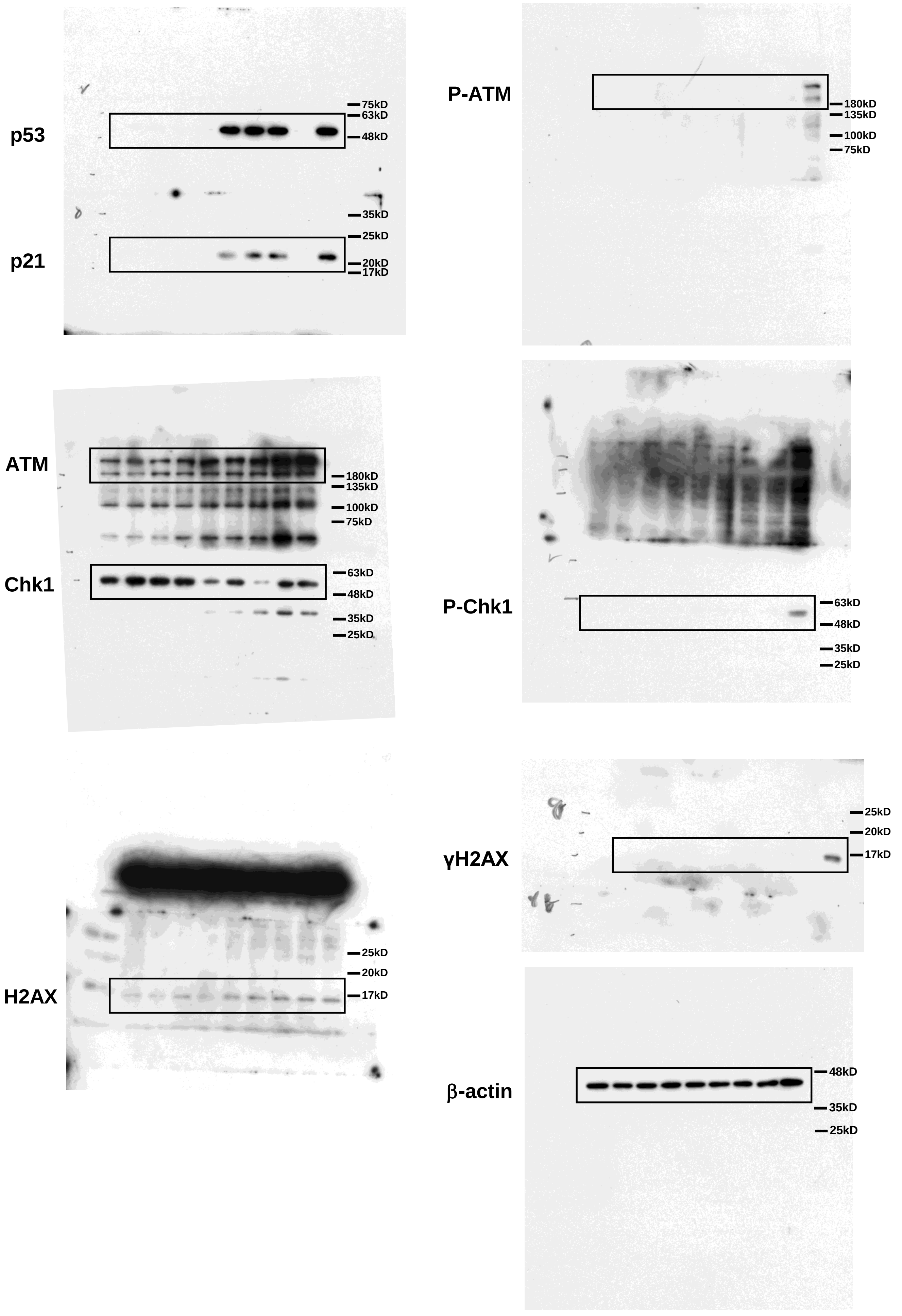

Extended data Figure 6f

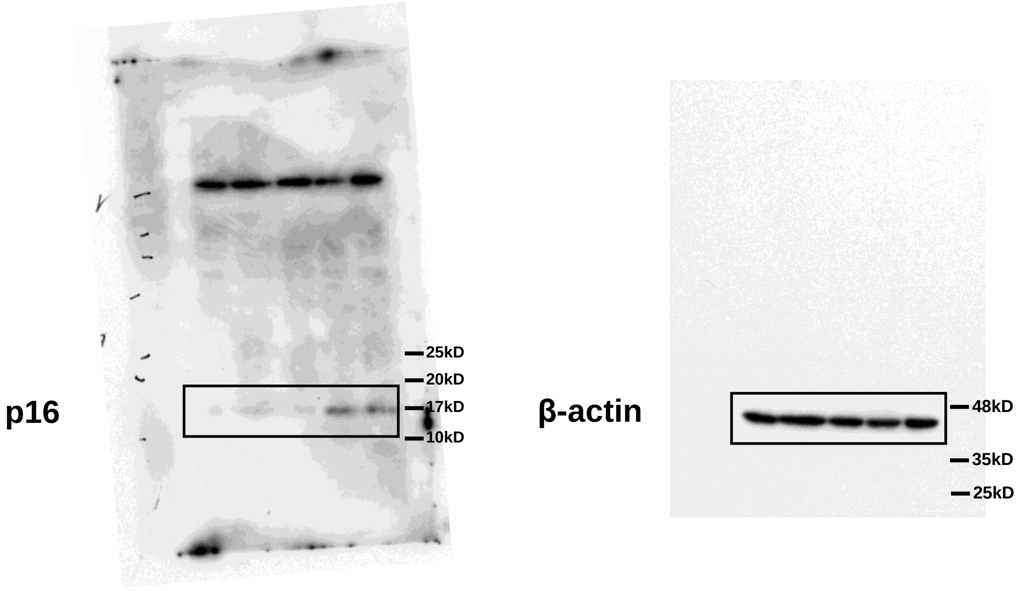

Extended data Figure 7b

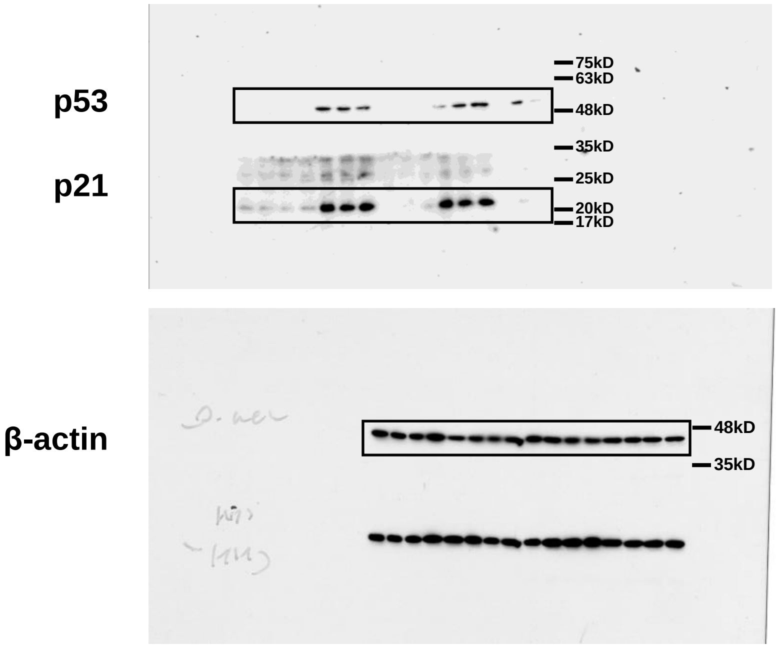

Extended data Figure 7c

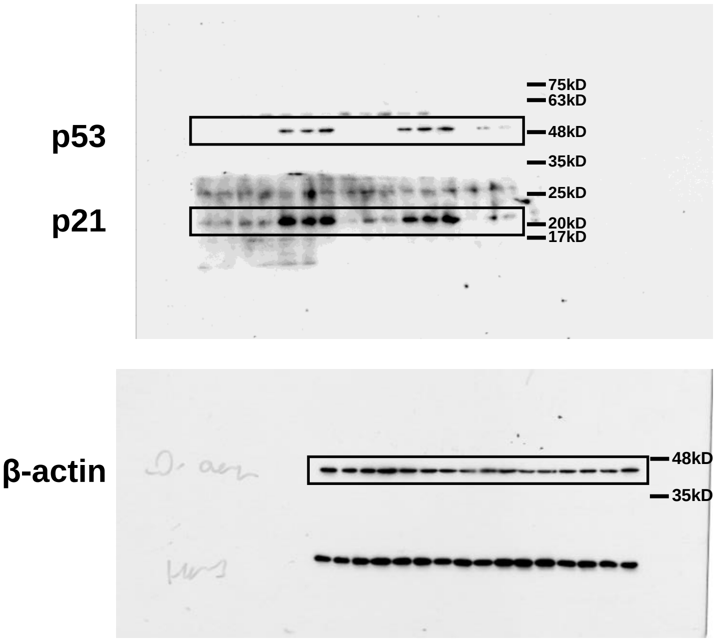

Extended data Figure 7d

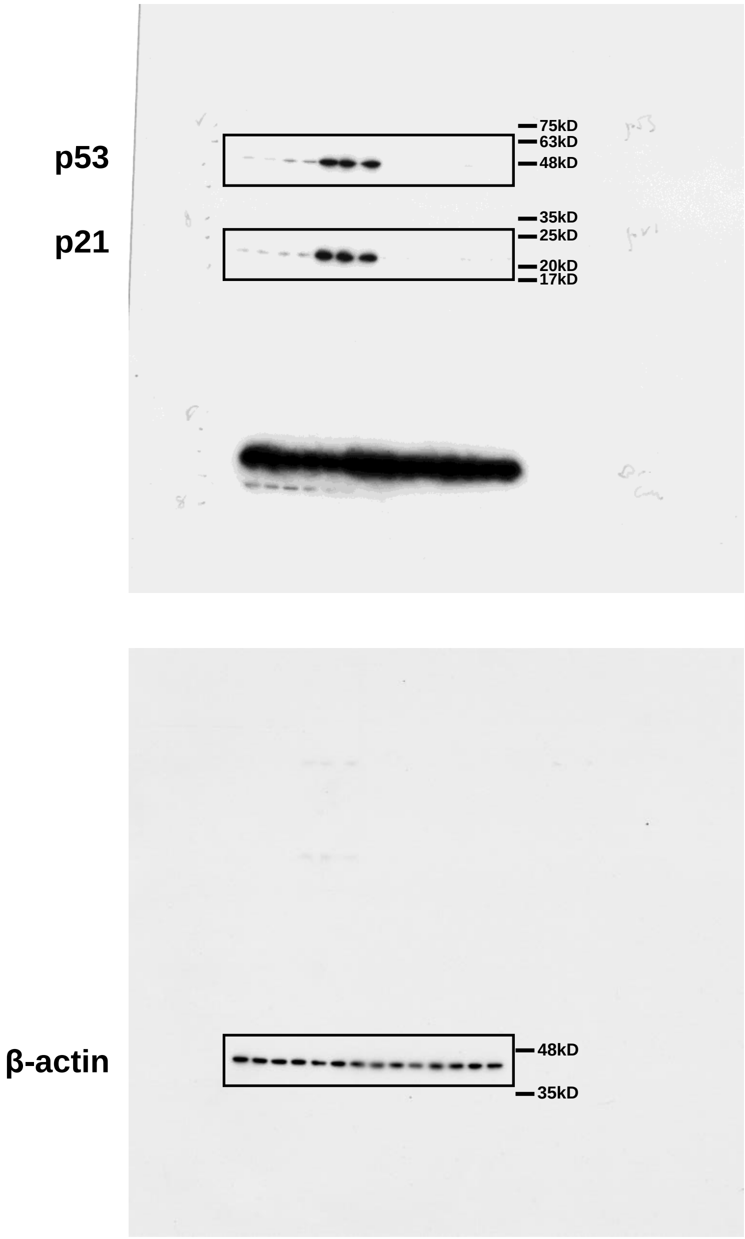

Extended data Figure 8g

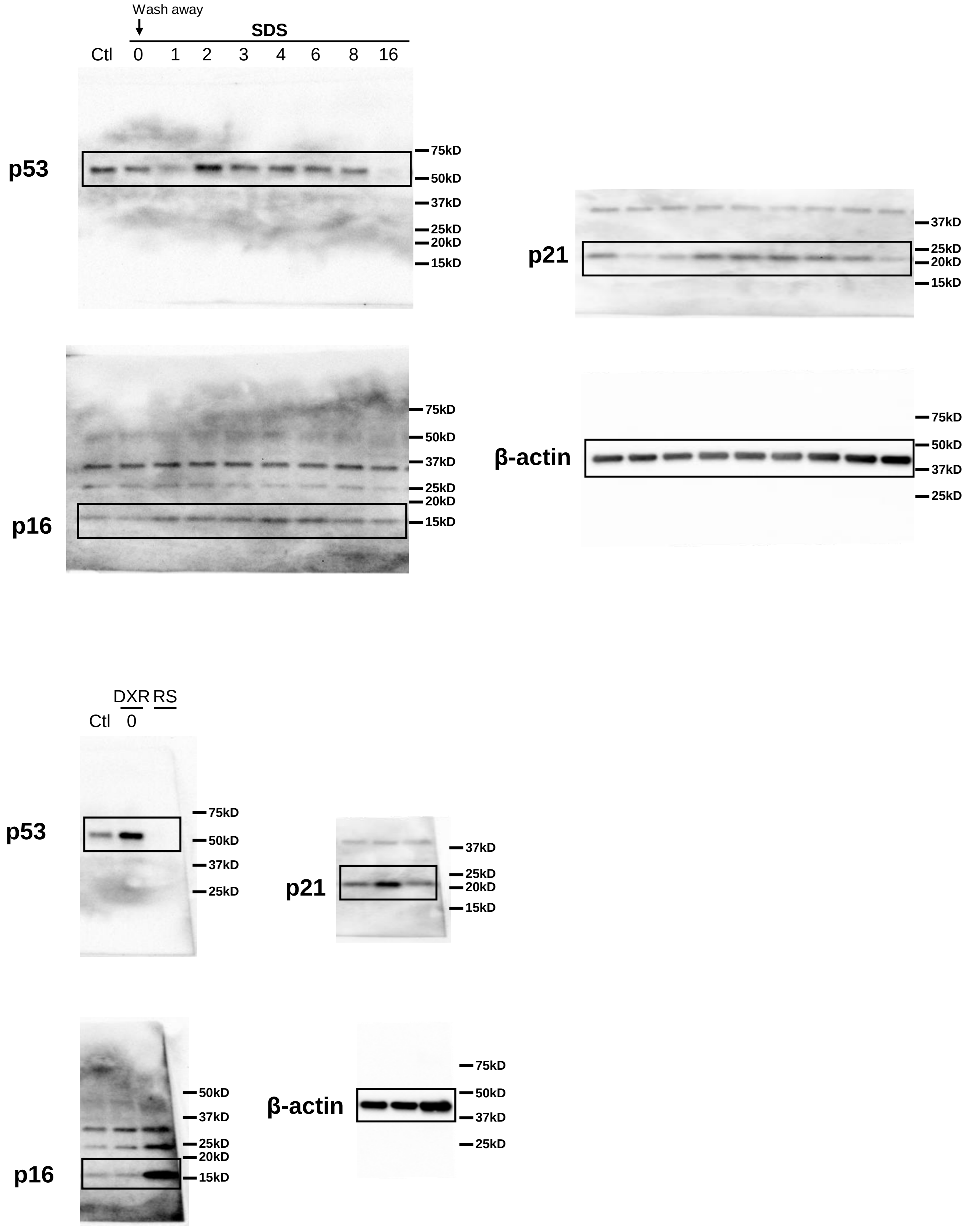

Extended data Figure 8g

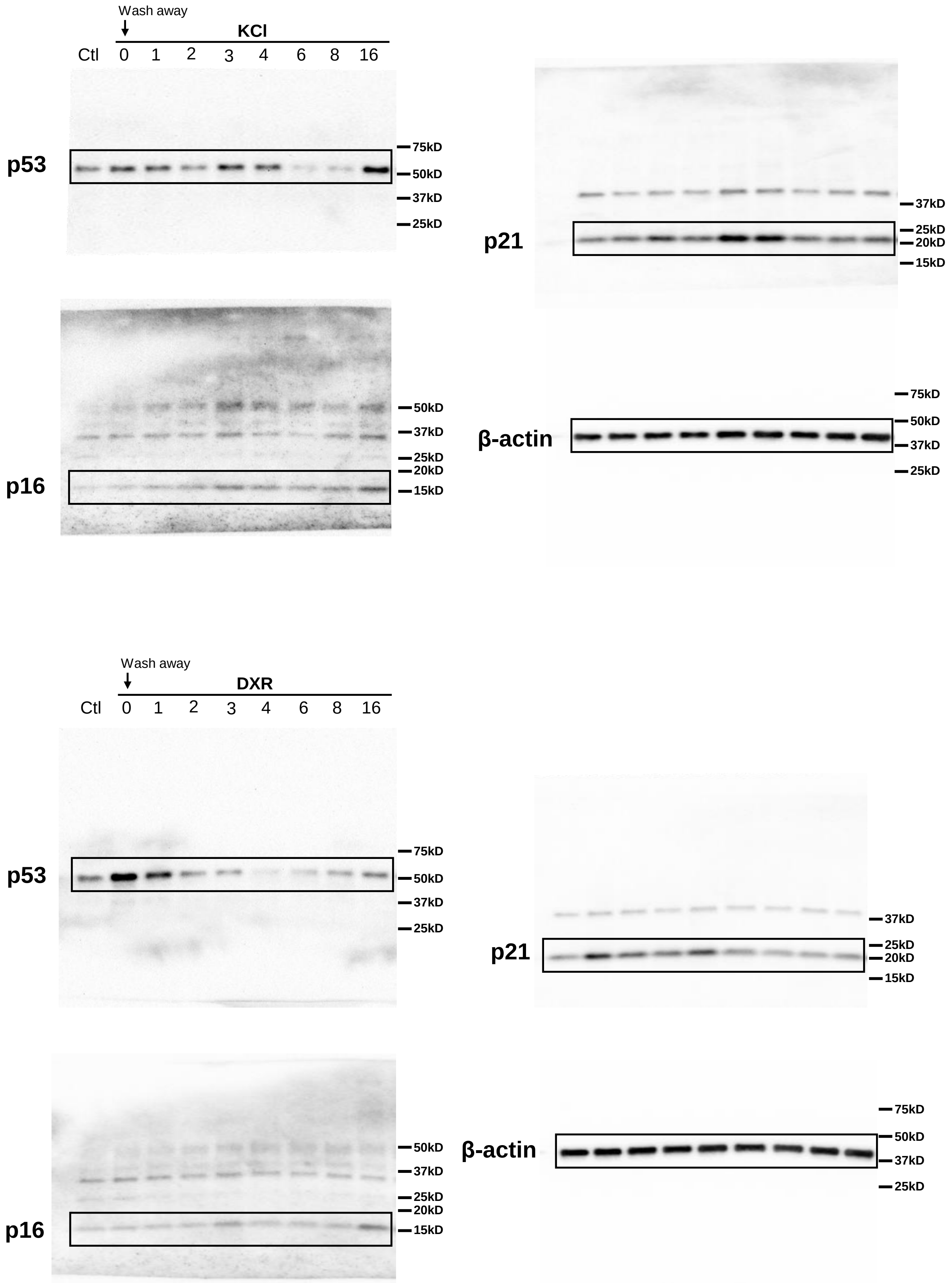

Supplement: Supplementary file 10 — Unprocessed blots for Figs. 3–6 and Extended Data Figs. 6–8. [file 43587_2024_575_MOESM10_ESM.pdf]
